# The *in vitro* antimicrobial activity of linezolid against unconventional pathogens

**DOI:** 10.7717/peerj.18825

**Published:** 2025-02-12

**Authors:** Ting Wang, Huiyue Zhang, Rui Feng, Jieru Ren, Xinping Xu, Shujuan Sun

**Affiliations:** Shandong Second Provincial General Hospital, Jinan, China

**Keywords:** Linezolid, *Mycobacterium tuberculosis*, NTM, Nocardia, Corynebacterium, Anaerobe

## Abstract

Linezolid is an oxazolidinone antibiotic that is mainly permitted to treat Gram-positive bacterial infections. Recent studies have shown that linezolid also has antibacterial effects on several other bacteria outside the package insert, including *Mycobacterium tuberculosis*, non-tuberculous mycobacteria (NTM), *Nocardia*, *Corynebacterium*, and anaerobes, *etc*. Interestingly, linezolid also has an *in vitro* inhibitory effect on fungi. This review focuses on the *in vitro* antibacterial activity of linezolid against microorganisms outside its antibacterial spectrum. We mainly listed the number of the tested strains, the minimum inhibitory concentration (MIC) range, MIC_50_, and MIC_90_ of linezolid against those pathogens outside the package insert. The results showed that among these tested pathogens, linezolid displayed strong inhibitory effects against *M. tuberculosis*, *Nocardia*, and *Corynebacterium*, with an MIC range of ≤2 μg/mL. As for NTM, linezolid exhibited moderate to potent inhibitory effects against the strains of different species with an MIC range of 0.06–128 μg/mL. Moreover, linezolid was reported to have a species-dependent inhibitory effect on anaerobes at a concentration range of 0.003–16 μg/mL. Furthermore, linezolid could enhance azoles and amphotericin B’s antifungal activity on *Candida* synergistically. It is hoped that this analysis can provide data for expanding the application of linezolid, make the off-label drug use have more compelling evidence, and provide clues for the development of new drugs.

## Introduction

Oxazolidinones represent a class of important synthetic bacterial protein synthesis inhibitors with a strong activity against Gram-positive bacteria (Liu et al., 2023). They were shown to have a distinct anti-bacterial mechanism of action, which differs from other protein synthesis inhibitors and prevents oxazolidinones from cross-resistance (Roger, Roberts & Muller, 2018). Oxazolidinones demonstrate good activity against the vast majority of Gram-positive microorganisms, including multidrug-resistant pathogens, vancomycin-resistant enterococcus, and methicillin-resistant *Staphylococcus aureus*. More excitingly, besides Gram-positive bacteria, linezolid has also been demonstrated to have potent *in vitro* activity against multiple microorganisms, such as *M. tuberculosis*, NTM, *Nocardia*, *Corynebacterium*, anaerobes, fungi, *etc*. For these findings, on one side, its activity against *M. tuberculosis* has been proven to be effective clinically and widely recommended. Linezolid-containing regimens are considered potential alternatives for the treatment of patients with multidrug-resistant tuberculosis (MDR-TB) or extensively drug-resistant tuberculosis (XDR-TB). The World Health Organization (2022) recommends that 6-month bedaquiline, pretomanid, linezolid, and moxifloxacin (BPaLM) regimen can be used for MDR-TB/rifampicin-resistant tuberculosis (RR-TB). On the other side, the activity against NTM of linezolid has been proved and recommended by the guidelines for clinical application. Clinical practice guidelines recommend that 600 mg of linezolid taken once or twice per day can be used for nontuberculous mycobacterial pulmonary disease (Haworth et al., 2017; Chinese Society for Tuberculosis CMA, 2020; Daley et al., 2020). Some studies, although not yet applied to clinical practice and not included in guidelines, have already been reported as case reports. There have been a few case reports about other antibacterial activities of linezolid against *Nocardia* and *Corynebacterium* that have been clinically proven to be effective (Davidson et al., 2020; Margalit et al., 2021; Fan et al., 2022; Tabaja et al., 2022). The expanding use of linezolid mentioned above makes us believe it has more extensive application, and as we know, the development of new drugs is a time-consuming, costly, and low-success-rate process, and it is of great importance to maximize the potential use of antimicrobial drugs. Therefore, based on those published data, we summarize the extensive antimicrobial activities of linezolid outside the traditional antimicrobial spectrum published, including MIC range, MIC_50_, MIC_90_, the time of publication, the source of the strains, *etc*. We hope that this summary will supply data for expanding the application of linezolid and provide a valuable approach to empowering drug discovery and development.

## Survey methodology

Suitable literature from 1 January 1999 to 1 January 2023 was searched from the Web of Science, Cochrane Library, PubMed, EMBASE, and MEDLINE databases. We used a broad range of keywords, including “linezolid”, “*mycobacterium tuberculosis*”, “non-tuberculous mycobacteria”, “*Nocardia*”, “*corynebacterium*”, “anaerobes”, “fungi”, “MIC”, and “*in vitro* antibacterial activity”, along with using “+”, “AND”, and “OR” for a specific search result. All the relevant articles were searched and thoroughly reviewed. Non-English papers, case reports, systematic reviews, and preprints were excluded from the search results. Detailed information, including the number of the strains, especially the MIC range, MIC_50_, and MIC_90_, is summarized in Tables S1 and S2 within the Supplemental Information. Besides that content, more details, including the data on the susceptibility of the other strains with low isolation rates and some studies that lack critical information, particularly those without MIC values, are presented in Tables S3 and S4 of the Supplemental Information.

### M. tuberculosis

*M. tuberculosis* is a kind of slender and slightly curved Gram-positive bacillus, named for its branched arrangement. *M. tuberculosis* can be transmitted through the respiratory tract, digestive tract, damaged skin, mucous membrane, genitals, and other contact infections. The most significant mode of transmission among them is through dust and droplets entering the respiratory system.

Drug-resistant tuberculosis is still a major public health issue, that has a detrimental impact on individuals, communities, and healthcare systems. According to recent estimates, there were around 500,000 new cases of MDR/RR-TB worldwide in 2018. Patients with MDR-TB, and especially those with XDR-TB strains, have extremely few treatment options. The situation is made more difficult by the growing number of immunocompromised people, which poses a great challenge to clinical treatment. Clinically, combination therapy is frequently used to treat tuberculosis that is resistant to drugs. However, there are few new drugs available on the market.

In recent years, extended studies have shown that linezolid has a good effect on *M. tuberculosis in vitro* and acts as a part of regimens that were recommended by the guide. From the published data we have collected, the sensitivity of linezolid against *M. tuberculosis* comes from many countries and regions. Numerous *in vitro* studies have demonstrated that linezolid has a strong anti-mycobacterial effect with an MIC_90_ of 0.25–2 μg/mL. The minimal MIC of linezolid observed for *M. tuberculosis* was 0.03 μg/mL (Zheng et al., 2021). Most of the data were from China, where the MIC_50_ ranged from 0.12 to 0.5 μg/mL (Huang et al., 2008; Yang et al., 2012; Zhang et al., 2014a, 2014b; Pang et al., 2017; Zong et al., 2018; Guo et al., 2021a; Yao et al., 2021; Zheng et al., 2021; Wang et al., 2022a; An et al., 2023; Guo et al., 2023), while a study from Shanghai showed that MIC_50_ values were 1 μg/mL (Guo et al., 2023). Linezolid also exhibited potent antibacterial activity against MDR-TB or XDR-TB, with MIC_90_ ranging from 0.25 to 1 μg/mL. Similarly, linezolid possessed potent activity against the strains from Japan and Korea (MIC = 0.125–2 μg/mL) (Yang et al., 2018; Aono et al., 2022). The MICs of linezolid against XDR strains and pre-XDR strains from Pakistan were both found to be 0.5 μg/mL (Ahmed et al., 2013). Linezolid showed an MIC range of 0.12–1 μg/mL against strains from Spain (Alcalá et al., 2003; Tato et al., 2006). One study from Turkey has shown that the MICs of linezolid against the strains are 0.5 μg/mL (Alcalá et al., 2003; Tato et al., 2006), whilst there were higher MIC_50_ and MIC_90_ in another study, at 4 and 8 μg/mL, respectively (Erturan & Uzun, 2005). It is worth noting that linezolid showed strong activity against *M. tuberculosis* from the United States, with MIC_50_ and MIC _90_ both at 0.25 μg/mL (*n* = 153) (Cavanaugh et al., 2017).

But gradually, linezolid-resistant tuberculosis strains were identified. According to the results of the current meta-analysis study, the total estimate of linezolid resistance among MDR-TB clinical isolates was 4.2%, while China had the highest resistance to linezolid among 14 different countries (Azimi et al., 2022). According to existing studies, linezolid exhibited excellent activity against the strains from India, with a resistance of approximately 3.1–7.5% (Nambiar et al., 2021; Bhanushali et al., 2024). Particularly, multidrug resistance is linked to Beijing strains, which account for over 50% of the strains in East Asia, and at least 13% of the strains globally (Parwati, van Crevel & van Soolingen, 2010). Zhang et al. (2014a) discovered that the Beijing genotype was substantially linked to both a new non-synonymous substitution, and linezolid resistance in MDR-TB and XDR-TB. It initially turned out that His155Asp in rplC was involved in low-level linezolid resistance (2 µg/mL) in *M. tuberculosis* (Zhang et al., 2014a). Furthermore, since *M. tuberculosis* only possesses one copy of the ribosomal RNA (rrn) operon, which codes for 23S rRNA, a single mutation would prevent the action of linezolid. Linezolid-resistance-related genes have been confirmed by identifying mutations in the rrl (encoding 23S rRNA) and rplC (encoding ribosomal protein L3) genes in both clinical and *in vitro*-selected linezolid-resistant tuberculosis isolates (Beckert et al., 2012; Zhang et al., 2014a; McNeil et al., 2017). Nevertheless, the potential role of linezolid against *M. tuberculosis* requires constant monitoring.

Linezolid is an inhibitor of bacterial protein synthesis. It binds to the 50S subunit of the ribosome of *M. tuberculosis*, inhibits the connection of mRNA to the ribosome, and prevents the 70S initiation complex from forming, which stops the synthesis of bacterial proteins during the early stages of translation. Due to the unique site and mode of action of the drug, there hasn’t yet been any evidence of cross-resistance with widely used anti-tuberculosis medications, and it is not easy to induce bacterial resistance *in vitro* (Singh et al., 2019). In 2016, the World Health Organization listed linezolid as the core treatment drug for MDR-TB. The World Health Organization (2022), suggested that patients with MDR-TB that was resistant to fluoroquinolones be treated for 6 to 9 months with regimens that included linezolid. Finally, it is critical to acknowledge that linezolid has a bright future in tuberculosis treatment, and additional research is needed to support potential mechanisms of resistance.

### NTM

NTM are environmental microbes that occur predominantly in water and soil (Honda, Virdi & Chan, 2018; Zhang et al., 2020). With a few NTM species being connected to infectious cases, the total number of NTM species known to science is over 190 (Daley et al., 2020). NTM infection has been observed in both immunocompetent and immunodeficient people. While some NTM induce extrapulmonary infections, the majority of NTM cause pulmonary infections. Individuals with compromised immunity due to HIV infection, organ transplant recipients, patients with malignant tumors, patients with long-term lung conditions, and the elderly are more vulnerable to NTM infection (Wang et al., 2014; Adjemian et al., 2017).

NTM can be classified as slow-growing *Mycobacterium* (SGM, colony-forming time greater than 7 days) or rapidly-growing *Mycobacterium* (RGM, colony-forming time less than 7 days) based on how long it takes for colonies to form in the culture medium. This classification is related to clinical diagnosis and treatment. The available data demonstrates that among all NTM, *M. intracellulare*, *M. avium*, *M. fortuitum*, *M. kansasii*, and *M. abscessus* were the most common isolates. *M. abscessus* complex, *M. chelonae*, and *M. fortuitum* are the prevalent rapidly-growing mycobacteria. *M. abscessus* complex is specifically separated into three subspecies: *M. abscessus*, *M. bolletii*, and *M. massiliense*. Common clinical pathogenic slow-growing mycobacteria include *M. avium* complex, *M. kansasii*, *M. marinum*, *M. arupense*, and so on. Multiple studies from various countries have shown that linezolid produced inhibitory effects on NTM *in vitro* and led to effective clinical results.

In general, linezolid exhibited species-dependent effects against NTM, with an MIC range of 0.0625–128 μg/mL. Linezolid showed inhibitory effects against *M. abscessus* at an MIC_90_ of 4–64 μg/mL. All of the *M. abscessus* isolates were divided into three subspecies: *M. abscessus subsp. abscessus*, *M. abscessus subsp. bolletii*, and *M. abscessus subsp. Massiliense*. Regarding the antibacterial activity of linezolid against *M. abscessus subsp. abscessus*, more data was obtained from China, with sensitivity ranging from 29.85% to 93% at an MIC_90_ of 8–32 μg/mL (Guo et al., 2021a, 2021b; Nie et al., 2014; Lee et al., 2017; Li et al., 2017; Zhang et al., 2017; Bender et al., 2018; Liu et al., 2021; Gao et al., 2023). In other countries, linezolid displayed weak *in vitro* efficacy against *M. abscessus subsp. abscessus* in over 90% of the isolates with an MIC_90_ range of 16–64 μg/mL (Vera-Cabrera et al., 2006a; Brown-Elliott & Wallace, 2017b; Lee et al., 2017; Brown-Elliott, Rubio & Wallace, 2018; Tang et al., 2018; Ruth et al., 2020; Kim et al., 2021; Senol et al., 2022; Hunkins et al., 2023). In view of one study, the MIC_50_ and MIC_90_ were both 64 μg/mL (Vera-Cabrera et al., 2006a). As for *M. abscessus subsp. massiliense*, linezolid was reported to have a moderate effect on the isolates at a MIC_90_ of 4–32 μg/mL (Zhang et al., 2017; Gao et al., 2023; Lee et al., 2017; Guo et al., 2021b; Kim et al., 2021; Brown-Elliott & Wallace, 2017b; Brown-Elliott, Rubio & Wallace, 2018; Hunkins et al., 2023; Tang et al., 2018). Equally, the resistance rate to linezolid was from 20% to 80% for *M. abscessus subsp. bolletii* with an MIC of 4–32 μg/mL (Nie et al., 2014; Lee et al., 2017; Tang et al., 2018; Hunkins et al., 2023). As for the *M. abscessus* complex, linezolid showed inhibitory effects against them, with MIC_90_ ranging from 16 to 32 μg/mL (Shen et al., 2018; Zhang et al., 2022; He et al., 2022; Marfil et al., 2022; Gao et al., 2023). With regard to the *M. chelonae*, linezolid exhibited moderate activity against the isolates with an MIC_90_ range of 8–64 μg/mL (Ruth et al., 2020; Brown-Elliott & Wallace, 2017b; Zhao et al., 2015; Zhang et al., 2022; Hunkins et al., 2023; Brown-Elliott, Rubio & Wallace, 2018; Heidarieh et al., 2016; Araj et al., 2019). In addition, the *in vitro* antibacterial activity against linezolid was also observed for *M. chelonae* from the United States (*n* = 526) with an MIC_90_ of >16 μg/mL (Hunkins et al., 2023). Zheng et al. (2017) reported the minimal MIC of *M. fortuitum* values of 0.0625 μg/mL. Among the other strains, linezolid exhibited *in vitro* antibacterial activity against *M. fortuitum*, with an MIC range of 0.0625–64 μg/mL (Zhang et al., 2022; Zhao et al., 2015; Zheng et al., 2017; Heidarieh et al., 2016; Ruth et al., 2020; Shen et al., 2018; Brown-Elliott & Wallace, 2017b; Senol et al., 2022; Hunkins et al., 2023; Araj et al., 2019). While Zheng et al. (2017) found that the MIC_50_ and MIC_90_ of linezolid against *M. fortuitum* were both 64 μg/mL (Zheng et al., 2017). The slow-growing mycobacteria with the highest isolation rate was the *M. avium* complex (MAC), including *M. avium* and *M. intracellulare*. Linezolid did not show good sensitivity to *M. avium*, with an MIC_90_ of 32–64 μg/m (Zhao et al., 2015; Zhang et al., 2015; Ruth et al., 2020; Litvinov et al., 2018; Cho et al., 2018; Kim et al., 2021; Huang et al., 2018; Senol et al., 2022; Brown-Elliott, Rubio & Wallace, 2018). The *in vitro* antibacterial activity against linezolid was also observed for *M. avium* with an MIC_90_ of 4 mg/L (Zhang et al., 2015). Compared with *M. avium*, linezolid had similar inhibitory activity against *M. intracellulare* at an MIC_90_ of 16–64 μg/mL (Zhao et al., 2015; Zhang et al., 2015; Litvinov et al., 2018; Cho et al., 2018; Kim et al., 2021; Araj et al., 2019; Brown-Elliott, Rubio & Wallace, 2018; Ruth et al., 2020; Huang et al., 2018; Senol et al., 2022). Linezolid inhibited *M. avium* complex over an MIC_90_ range of 32–128 μg/mL, which was higher than that of its separate subspecies (Vera-Cabrera et al., 2006a; Zhang et al., 2022; He et al., 2022; Brown-Elliott, Rubio & Wallace, 2018; Brown-Elliott et al., 2003; Brown-Elliott & Wallace, 2017b). From the available data, linezolid was active against *M. gordonae* with an MIC range of 0.5–16 μg/mL (Zhang et al., 2022; Senol et al., 2022; Brown-Elliott et al., 2003). The *in vitro* antibacterial activity of linezolid was also observed for *M. kansasii* isolates, with an MIC_90_ of 1–32 μg/mL (Zhao et al., 2015; Liu et al., 2021; Zhang et al., 2022; Litvinov et al., 2018; Kim et al., 2021; Brown-Elliott, Rubio & Wallace, 2018; Brown-Elliott et al., 2003; Brown-Elliott & Wallace, 2017b; He et al., 2022; Senol et al., 2022; Heidarieh et al., 2016). Linezolid also exhibited potent antibacterial activity against *M. marinum*, with an MIC of 0.125–16 μg/mL (Brown-Elliott et al., 2003; Gitti et al., 2011; Brown-Elliott & Wallace, 2017b; Brown-Elliott, Rubio & Wallace, 2018; Zhang et al., 2022).

Many species of NTM are increasingly being recognized as important human pathogens (Heidarieh et al., 2016). An invaluable addition to the arsenal of antimicrobials used in the treatment of NTM is linezolid (Brown-Elliott & Wallace, 2017b). According to the summarized data, NTM sensitivity of linezolid varies significantly worldwide, and these variations may be caused by a variety of factors, such as regional environmental conditions, antibiotic treatment regimens, and economic development levels. The prevalence of mycobacterial species varies, and the susceptibility to linezolid varies significantly by geographic region in different cities (Lai et al., 2010; Yu et al., 2016). In addition, the methodology or sample size may also affect the results of MIC. It is important to follow the Clinical and Laboratory Standards Institute (CLSI) standards when conducting drug sensitivity tests, because obtaining accurate susceptibility results is crucial for the selection of clinical antimicrobials, and the efficacy of treatment.

#### Nocardia

The *Nocardia* genus includes more than 90 species of bacteria, of which at least 54 have been shown to be clinically significant (Bender et al., 2018; Conville et al., 2018; Hamdi et al., 2020). They typically appear in immunocompromised hosts as an opportunistic infection pathogen (Wilson, 2012). Modes of entry include inhalation, ingestion of food, and direct infection through the skin. The most typical entrance method is inhalation (Duggal & Chugh, 2020). *N. nova* complex, *N. abscessus*, *N. transvalensis* complex, *N. farcinica*, *N. cyriacigeorgica*, and *N. brasiliensis* comprise the majority of isolates (Brown-Elliott et al., 2006). Different *Nocardia* species have distinct patterns of geographic prevalence, pathogenic traits, and antimicrobial susceptibility (Conville et al., 2018). According to the data gathered, *N. abscessus*, *N. beijingensis*, *N. brasiliensis*, *N. cyriacigeorgica*, *N. farcinica*, *N. novo*, *N. otitidiscaviarium*, *N. pseudobrasiliensis*, and *N. wallacei* are the *Nocardia* that are most isolated. Trimethoprim sulfamethoxazole (TMP-SMX), amikacin, imipenem, cephalosporin, minocycline, and so on are among the conventional regimens for *Nocardia*. The prognosis is still dismal because of the rise in medication resistance (Brown-Elliott et al., 2006; Peleg et al., 2007; Bender et al., 2018).

Many studies have revealed that linezolid displayed significant *in vitro* activity against nearly 100% of *Nocardia* isolates. It is worth noting that the susceptibility rate of *N. abscessus* to linezolid was nearly 100%, with an MIC of 0.19–2 μg/mL (Mazzaferri et al., 2018; Wei et al., 2021). Similarly, *N. cyriacigeorgica* was susceptible to linezolid at a MIC range of 0.094–4 μg/mL (Goodlet et al., 2021; Yi et al., 2019; Wang et al., 2022b; Brown-Elliott & Wallace, 2017a; Mazzaferri et al., 2018; Wei et al., 2021, 2017; Lao et al., 2022; Kuo et al., 2022), and only one study from Japan reported the strains with both MIC_50_ and MIC_90_ at 4 μg/mL (Toyokawa et al., 2021). Linezolid exhibited potent antibacterial activity against *N. farcinica* strains, with an MIC of 0.064–4 μg/mL (Goodlet et al., 2021; Yi et al., 2019; Kuo et al., 2022; Wang et al., 2022b; Brown-Elliott & Wallace, 2017a; Mazzaferri et al., 2018; Lao et al., 2022; Wei et al., 2021; Li et al., 2022; Mazzaferri et al., 2018; Wei et al., 2017). For *N. nova* strains, linezolid was more effective with an MIC value of 0.25–2 μg/mL (Yi et al., 2019; Lao et al., 2022; Wei et al., 2021, 2017), and *N. nova* complex also had low MIC values of 0.025–4 μg/mL for linezolid (Wang et al., 2022b; Brown-Elliott & Wallace, 2017a; Toyokawa et al., 2021). Linezolid possessed significant antibacterial activity against *N. brasiliensis*, with an MIC range of 0.12–8 μg/mL (Kuo et al., 2022; Lao et al., 2022; Toyokawa et al., 2021; Yi et al., 2019; Wang et al., 2022b; Brown-Elliott & Wallace, 2017a; Wei et al., 2021; Vera-Cabrera et al., 2006b). For the *N. otitidiscaviarum*, linezolid was the antibiotic frequently identified as being active at a MIC range of 0.5–8 μg/mL (Yi et al., 2019; Goodlet et al., 2021; Toyokawa et al., 2021; Wang et al., 2022b; Wei et al., 2021; Lao et al., 2022; Wei et al., 2017). In addition, the *Nocardia* strains with low isolation rates were all susceptible to linezolid with an MIC of ≤2 μg/mL, mostly, which were listed in Supplemental Information.

Even though most clinical infections caused by *Nocardia* can be effectively treated with sulfonamides, not all patients responded well to this therapeutic approach. When patients with central nervous system *Nocardia* infections, including brain abscesses, were treated simply with sulfonamide, the mortality rate was close to 50%, and the majority of patients needed surgery for drainage. Also, while using sulfonamides alone, individuals with non-central nervous system overwhelming or disseminated illnesses had a significant death rate (Vera-Cabrera et al., 2001; Moylett et al., 2003). According to published data, linezolid seems to be a good substitute for TMP-SMX when treating nocardiosis (Jodlowski, Melnychuk & Conry, 2007). The use of linezolid as a treatment is crucial for patients who are unable to tolerate TMP-SMX, have a history of sulfonamide allergy, or are not responding to treatment. These results proved that linezolid not only is a good alternative to sulfa antibiotics for the treatment of nocardiosis, but also is acceptable as initial empiric therapy for nocardiosis (De La Cruz, Minces & Silveira, 2015). However, it should be noted that linezolid-resistant *Nocardia* isolate has been reported. Recent research shows that strains of *N. beijingensis, N. cyriacigeorgica, N. pseudobrasiliensis*, and *N. veterana* showed drug resistance to linezolid (Yi et al., 2019; Harris et al., 2021; Lao et al., 2022). A strain of *N. cyriacigeorgica* from Yantai, China, and a strain of *N. veterana* isolated from Taiwan were reported to exhibit resistance to linezolid with an MIC of 16 μg/mL (Yi et al., 2019; Harris et al., 2021). Although linezolid-resistant strains are rare to be found, it is worthwhile to investigate the resistance mechanism of these strains.

#### Corynebacterium

*Corynebacterium* species are part of the microbiota of the skin and mucous membranes, and have often been thought to be contaminants in clinical specimens (Suwantarat et al., 2016; Barberis et al., 2018). Until now, more than 100 species have been identified, of which 54 are related to human infection (Asgin & Otlu, 2020). This species has long been underestimated due to the unreliability of conventional identification methods. Owing to the extensive use of immunosuppressants and modern diagnostic methods, the infection of *Corynebacterium* is gradually increasing. *C. amycolatum*, *C. jeikeium*, *C. striatum*, and *C. urealyticu* are reportedly the major isolates of *Corynebacterium* species (Fernandez-Roblas et al., 2009; Neemuchwala et al., 2018). In general, the coryneform bacteria collected from clinical samples typically show high and varied resistance to antimicrobials. Many of them show varying degrees of resistance to widely used antibiotics, such as fluoroquinolones, macrolides, and β-lactams (Gómez-Garcés, Alos & Tamayo, 2007). *C. jeikeium*, *C. urealyticum*, and *C. amycolatum* are examples of the above, usually exhibiting multiple drug resistance, showing resistance to β-lactams, macrolides, aminoglycosides, fluoroquinolones, and tetracycline, as well as clindamycin, and available options including vancomycin and linezolid. As a result, there are few treatment options for coryneform bacteria, particularly in the nosocomial setting, where multidrug-resistant pathogens are prevalent. Emerging data suggest that linezolid displayed potent activity against *corynebacterium in vitro*. Some case reports provide us with the evidence that treatment options for *C. striatum* could be feasible with linezolid as the drug of choice (Biscarini et al., 2021; Streifel et al., 2022).

Linezolid exhibited potent antibacterial activity against most *Corynebacterium* species reported, with MICs in the range of 0.0625–2 μg/mL, and *C. amycolatum* isolates had low MIC values of 0.064–2 μg/mL for linezolid (Biscarini et al., 2021; Streifel et al., 2022), except for five strains at an MIC range of 0.4–4 μg/mL (Johnson et al., 2003). Similar susceptibility results were shown in tests of linezolid against *C. jeikeium* with an MIC range of 0.064–2 μg/mL (Fernandez-Roblas et al., 2009; Gómez-Garcés, Alos & Tamayo, 2007; Sánchez Hernández et al., 2003; Goldstein et al., 2004; Johnson et al., 2003; Neemuchwala et al., 2018; Sun et al., 2022). Equally, all the *C. sriatum* isolates reported were susceptible to linezolid, with MICs ranging from 0.063 to 2 μg/mL (Johnson et al., 2003; Gómez-Garcés, Alos & Tamayo, 2007; Fernandez-Roblas et al., 2009; Nhan et al., 2012; Barberis et al., 2018; Neemuchwala et al., 2018; Suh et al., 2019; Abe et al., 2021; Wang et al., 2021; Sun et al., 2022). *C. urealyticum* is a cause of urinary tract infection and encrusting cystitis or pyelitis (López-Medrano et al., 2008). MIC values of linezolid against *C. urealyticum* were in the range of 0.015–1 μg/mL (Sánchez Hernández et al., 2003; Gómez-Garcés, Alos & Tamayo, 2007; Fernandez-Roblas et al., 2009; Neemuchwala et al., 2018; Chapartegui-González et al., 2020; Sun et al., 2022). Specifically, a study reported that 40 strains of *C. urealyticum* were almost all multidrug resistant, whereas linezolid exhibited excellent activity against those strains with an MIC_90_ of 1 μg/mL, and only one resistant strain at an MIC of 256 μg/mL (Chapartegui-González et al., 2020). Although linezolid has a lower renal excretion rate, several studies have already described clinical success of urinary tract infections by using it (Pontefract, Rovelsky & Madaras-Kelly, 2020; Wingler et al., 2021), indicating the potential of linezolid in the treatment of urinary tract infections induced by *C. urealyticum*.

Overall, most coryneform organisms under study had low MICs, indicating that linezolid is generally effective against these strains. The data we summarized highlights its option as a therapeutic alternative for vancomycin (Chapartegui-González et al., 2020; Milosavljevic et al., 2021). The drug may be administered orally or parenterally, and can be used in both hospitalized patients and sequential treatment regimens.

### Anaerobes

Anaerobes are opportunistic pathogens that may collaborate with aerobic bacteria to cause a variety of human illnesses, including cutaneous, pelvic, intraabdominal, and brain abscesses. Moreover, they have the ability to induce monomicrobial infections, which include bone and joint infections, deep tissue infections, and bacteremia (Reissier et al., 2023). The important compounds in the treatment of anaerobic infections include certain β-lactams, metronidazole, and clindamycin; however, a growing number of these organisms are becoming resistant to antibiotics globally.

Research revealed that linezolid was effective against some Gram-negative anaerobes as well as some Gram-positive ones, including *Bacteroides spp*., *Prevotella spp*., and *Fusobacterium nucleatum*. Linezolid was found to exhibit good action against several Gram-negative anaerobes and some Gram-positive anaerobes, such as *Fusobacterium nucleatum*, *Prevotella spp*., and *Bacteroides spp*. Anaerobic infection treatment with linezolid was proven clinically successful in a case report (Wareham et al., 2005), but no evidence-based guidelines have been proposed.

The majority of anaerobic Gram-positive bacteria that were identified from clinical samples are *Clostridium spp*., *Actinomyces spp*., *Propionibacterium spp*., *Lactobacillus spp*., and *Eubacterium group spp*. Among *Clostridium*, *Clostridium difficile* and *Clostridium perfringens* account for a relatively high proportion. Linezolid was sensitive to *Clostridium perfringens*, both with MIC_50_ and MIC_90_ of 2 μg/mL (Edlund, Oh & Nord, 1999; Ednie, Jacobs & Appelbaum, 2002; Citron et al., 2003; Goldstein et al., 2004; Yong et al., 2004; Goldstein et al., 2005; Yum et al., 2010; Goldstein, Merriam & Citron, 2020). Linezolid demonstrated inhibitory activity against *Clostridium difficile*, with an MIC_50_ of 2 μg/mL and an MIC_90_ range of 2–16 μg/mL (Ednie, Jacobs & Appelbaum, 2002; Citron et al., 2003; Phillips et al., 2003; Goldstein et al., 2004; Yong et al., 2004; Goldstein et al., 2005; Mathur et al., 2011; Rashid et al., 2014; Goldstein, Merriam & Citron, 2020). Linezolid proved to have strong activity against *Actinomyces spp*., with MIC_50_ and MIC_90_ of 0.5 μg/mL, respectively, except for *Actinomyces israelii*, with MIC_90_ of 16 μg/mL (Citron et al., 2003; Goldstein et al., 2004, 2005). Linezolid has strong antibacterial activity against *Propionibacterium spp*., especially for *Propionibacterium acnes*, which has a high isolation rate, at an MIC of 0.25–1 μg/mL (Edlund, Oh & Nord, 1999; Ednie, Jacobs & Appelbaum, 2002; Goldstein et al., 2004, 2005; Oprica & Nord, 2005; Mathur et al., 2011). Linezolid exerted inhibitory activity against *Lactobacillus spp*., with an MIC range of 0.5–16 μg/mL (Citron et al., 2003; Goldstein et al., 2004, 2005). It can be seen that most *Eubacterium lentum* of *Eubacterium group spp*. isolates were highly susceptible to linezolid at a MIC_90_ of 0.5–2 μg/mL (Citron et al., 2003; Goldstein et al., 2004, 2005). MIC values of linezolid against *Peptostreptococcus spp*. were in the range of 0.25–16 μg/mL (Goldstein, Citron & Merriam, 1999; Ednie, Jacobs & Appelbaum, 2002; Citron et al., 2003; Phillips et al., 2003; Yong et al., 2004; Yum et al., 2010; Mathur et al., 2011; Lee et al., 2015). Gram-negative anaerobic bacteria that are clinically significant include the *Bacteroides fragilis* group, *Prevotella spp*., *Fusobacterium spp*., *Porphyromonas spp*., and *Veillonella spp*. (Gajdács, Spengler & Urbán, 2017). The MIC_90_ of linezolid was found to be 4 μg/mL against *Bacteroides fragilis* (Snydman et al., 2017; Wybo et al., 2014; Goldstein, Merriam & Citron, 2020; Goldstein et al., 2017; Phillips et al., 2003; Behra-Miellet, Calvet & Dubreuil, 2003; Yum et al., 2010; Molitoris et al., 2006; Yong et al., 2004; Ednie, Jacobs & Appelbaum, 2002). Linezolid proved to have a general antibacterial activity against *Prevotella spp*., with a MIC of 0.016–8 μg/mL (Behra-Miellet, Calvet & Dubreuil, 2003; Citron et al., 2003; Mathur et al., 2011; Molitoris et al., 2006; Goldstein, Citron & Merriam, 1999). The *in vitro* antibacterial activity of linezolid was also observed for *Porphyromonas spp*. with an MIC of 0.06–4 μg/mL (Goldstein, Citron & Merriam, 1999; Citron et al., 2003; Molitoris et al., 2006; Goldstein, Merriam & Citron, 2020). Linezolid was highly sensitive to *Fusobacterium spp*. at an MIC range of 0.016–2 μg/mL (Behra-Miellet, Calvet & Dubreuil, 2003; Goldstein, Merriam & Citron, 2020; Ednie, Jacobs & Appelbaum, 2002; Phillips et al., 2003; Goldstein, Citron & Merriam, 1999; Molitoris et al., 2006), except for some strains with an MIC of 8 μg/mL (Edlund, Oh & Nord, 1999; Wybo et al., 2014). Among the strains of *Veillonella spp*., only a few MICs of linezolid were greater than 2 μg/mL (Behra-Miellet, Calvet & Dubreuil, 2003; Goldstein, Merriam & Citron, 2020).

Linezolid has shown good activity against some anaerobes overall. Because anaerobic diseases are frequently polymicrobial and associated with aerobic bacteria, normal microbiological laboratories do not routinely perform the laborious tasks of detecting, identifying, and testing for medication susceptibility of anaerobic bacteria (Reissier et al., 2023). Therefore, the treatment of these infections is mostly empirical, including antibiotics with known efficacy against anaerobic bacteria. In recent years, anaerobic bacteria have become more resistant to antibiotics, such as carbapenems and nitroimidazoles (Cobo, 2022). The anti-anaerobic activity of linezolid is an encouraging finding. Our results show that linezolid possesses good antibacterial activity against some anaerobic bacteria, which can expand its clinical application, especially Gram-positive cocci combined with anaerobic bacterial infection, without requiring additional application of anti-anaerobic drugs.

### Fungus

The high death rate linked to invasive fungal diseases is especially concerning. Linezolid is a traditional antibacterial drug. Extensive efforts have been devoted to finding new antifungal activities of linezolid, such as *Cryptococcus neoformans var*., *C. albicans*, and *Pythium insidiosum*. Rossato et al. (2015) reported that amphotericin B combined with linezolid showed synergism in some *Cryptococcus neoformans var*. strains at a fractional inhibitory concentration index of 0.493. For *C. albicans*, linezolid combined with azoles induced *in vitro* synergistic effects against resistant *C. albicans* isolates, decreasing the MIC of azoles from >512 to 1 μg/mL (Lu et al., 2018). *Pythium* is a newly discovered pathogen; three species of *Pythium* have been identified in infected mammals: *Pythium aphanidermatum*, *Pythium insidiosum*, and *Pythium periculosum* (Miraglia et al., 2022). Research has demonstrated that linezolid shows significant antibacterial properties against all *Pythium insidiosum* strains with the lowest MIC_90_ values (4 μg/ml after 48 h) using the broth microdilution (BMD) and the E-test methods (Loreto et al., 2014). Linezolid has a promising *in vitro* activity against *P. insidiosum* (Hu et al., 2024).

### Others

Foodborne pathogen *Listeria monocytogenes* causes listeriosis, an illness that can result in fetal-placental infection in expectant mothers, meningoencephalitis in the elderly and immunocompromised people, and bacteremia in humans (Disson, Moura & Lecuit, 2021). Linezolid demonstrated excellent activity against *Listeria spp*., with a MIC of 0.5–2 μg/mL (Yu et al., 2021; Callapina et al., 2001; Jones, Biedenbach & Anderegg, 2002; Mendes et al., 2015). Furthermore, infections with the opportunistic pathogen *Rhodococcus equi* have been identified in a growing number of immunocompromised individuals. This pathogen was initially identified in foals that had pneumonia (Lin et al., 2019). Linezolid exhibited potent *in vitro* activity against *Rhodococcus equi* strains, with an MIC range of 0.5–2 μg/mL (Bowersock et al., 2000; Giacometti et al., 2005; Erol et al., 2021). In addition, *Micrococcus* are thought to be opportunistic pathogens (Tizabi & Hill, 2023), and linezolid was active against *Micrococcus spp*., with MICs ranging from 0.5–1 μg/mL (Jones, Biedenbach & Anderegg, 2002; Mendes et al., 2015).

## Discussion

Linezolid is an exceptionally effective drug against Gram-positive bacteria, which is characterized by its excellent tissue distribution and high oral bioavailability. It is a good choice when switching from intravenous therapy to oral treatment in order to decrease the duration and expense of hospitalization (Thirot et al., 2021). Numerous studies have revealed that linezolid has activity against a variety of pathogens beyond the scope of its antibiotic spectrum. All the benefits offered by linezolid encourage clinicians to use it in more circumstances. However, we need to be aware of the possible adverse effects of linezolid in clinical applications, such as hematological toxicity (anemia, thrombocytopenia), peripheral and optic neuropathy risk, and, in rare cases, hyperlactatemia. Additionally, linezolid is a reversible and non-selective monoamine oxidase inhibitor, which increases the risk of 5-HT syndrome in people who take other drugs presenting the same risk (Thirot et al., 2021).

At the same time, we found that the sensitivity of linezolid to strains of different genera from different countries and regions showed high differences. This discrepancy is attributed to the strain-specific traits, variations in testing methodologies, and the extent of the sample size. Furthermore, the issue of the resistance of linezolid in some pathogens cannot be ignored, including *M. tuberculosis*, NTM, and *Nocardia*. *Kadura* identified 17 different linezolid resistance mutations in rrl and/or rplC14 in *mycobacteria* (Kadura et al., 2020). The majority of mycobacterial strains that are resistant to linezolid have ribosome or associated gene alterations, including those in the rplC, rrl, and tsnR genes. A mutation in fadD32, which codes for a protein crucial to the manufacture of mycolic acid, was linked to one such mechanism (Gan, Ng & Ngeow, 2023). In a study conducted in India, using whole-genome sequencing of 32 isolates that were phenotypically resistant to linezolid, the main resistance determinants were found to be G2814T in the rrl gene and C154R in the rplC gene (Nambiar et al., 2021). Linezolid resistance has also been linked to mycobacterial efflux proteins. As for NTM, alterations were found in a drug pump-related system, indicating that drug efflux is a mechanism of linezolid resistance in *M. abscessus* (Negatu et al., 2023). The relevant research reported that alterations in the genes encoding FadD32 and 23S rRNA could be linked to the resistance of linezolid in *M. abscessus* (Ng & Ngeow, 2023). Toyokawa et al. (2024) discovered that the linezolid-resistant *Nocardia* strains lacked the alterations in the 50S ribosomal protein and 23S RNA. These findings suggest that the mechanism of resistance to oxazolidinone in *Nocardia spp*. differs from that observed in *enterococci* and *staphylococci*.

A thorough understanding of resistance mechanisms and efficient resistance monitoring are essential for improved clinical and public health management. Further research is necessary in order to completely comprehend the resistance mechanism of linezolid. Developing novel drugs or drug combinations based on resistance mechanisms is one strategy to slow the global spread of resistance. Future studies should also look into biomarkers for tracking the emergence of resistance early on, and the synergistic impact of additional antibiotics in treating fungal or anaerobic infections. Meanwhile, highlighting the need for future research, we must not overlook the significance of i*n vivo* studies for observing direct biological effects, the necessity of clinical trials to assess real-world application, and the value of comparative studies to determine the best therapeutic strategies among various antibiotics.

## Conclusion

Antibiotic resistance is increasing worldwide; the research on the new effects of old drugs has drawn global attention in recent years. Fully exploring the antibacterial spectrum of existing antibacterial drugs is an important approach for new antibacterial agent research. In this review, we summarized the sensitivity of linezolid against pathogens outside the antimicrobial spectrum. The results show that linezolid is not only effective against Gram-positive bacteria, but also has activity against tuberculosis, NTM, *Nocardia*, *Corynebacterium*, anaerobes, and fungi, as well as other pathogens. We believe that this research will be helpful not only in expanding linezolid’s application, but also in providing ideas for new drug development. Of course, more research is needed, especially *in vivo* research and clinical trials of linezolid against the unconventional pathogens mentioned above.

## Supplemental Information

10.7717/peerj.18825/supp-1 Supplemental Information 1 *I. vitro* activity of linezolid against *M. tuberculosis*, NTM and Nocardia (MIC, μg/mL).

10.7717/peerj.18825/supp-2 Supplemental Information 2 *I. vitro* activity of linezolid against Corynebacterium, Anaerobe and other pathogens (MIC, μg/mL).

10.7717/peerj.18825/supp-3 Supplemental Information 3 *I. vitro* activity of linezolid against *M. tuberculosis*, NTM and Nocardia (MIC, μg/mL).

## Additional Information and Declarations

### Data Availability

The following information was supplied regarding data availability:

This is a literature review.
