# Supplementary material for: The in vitro antimicrobial activity of linezolid against unconventional pathogens
Source: PeerJ. 2025 Feb 12;13:e18825. doi: 10.7717/peerj.18825 (PMC11829633; doi:10.7717/peerj.18825)
Supplement: Supplemental Information 1 [file peerj-13-18825-s001.docx]

Table 1 In vitro activity of linezolid against M. tuberculosis, NTM and Nocardia (MIC, μg/mL).

| **Organism** | **N** | **MIC range** | **MIC_50_** | **MIC_90_** | **Reference** |  | **Organism** | **N** | **MIC range** | **MIC_50_** | **MIC_90_** | **Reference** |
| --- | --- | --- | --- | --- | --- | --- | --- | --- | --- | --- | --- | --- |
| **M. tuberculosis** | 84 | 0.125-4 | 0.5 | 2 | *(Huang et al., 2008)* |  | Drug-sensitive | 9 | 0.25-0.5 | 0.25 | 0.5 | *(An et al., 2023)* |
| Beijing genotype | 102 |  | 0.5 | 2 | *(Zhang et al., 2014)* |  | Drug-susceptible | 10 | 0.095-0.3 |  |  | *(Guo et al., 2021)* |
| Non-Beijing genotype | 56 |  | 0.25 | 0.5 | *(Zhang et al., 2014)* |  | MDR | 30 | 0.036-0.499 |  |  | *(Guo et al., 2021)* |
| MDR | 45 | 0.125-0.5 | 0.25 | 0.25 | *(Yang et al., 2012)* |  |  | 69 |  | 0.5 | 1 | *(Wang et al., 2022)* |
| XDR | 16 | 0.125-0.5 | 0.25 | 0.25 | *(Yang et al., 2012)* |  |  | 169 | 0.125-> 2 | 0.5 | 1 | *(Singh et al., 2022)* |
| MDR | 15 | 0.063-16 | 0.25 | 4 | *(Zhang et al., 2014)* |  | MDR | 39 | 0.25 to >16 | 0.25 | 0.5 | *(Kazemian et al., 2015)* |
| XDR | 90 | 0.125-32 | 0.5 | 0.5 | *(Pang et al., 2017)* |  | MDR | 35 |  | 0.25 | 0.5 | *(Kardan-Yamchi et al., 2020)* |
| MDR | 120 |  | 0.064 | 1 | *(Zong et al., 2018)* |  | MDR | 54 | 0.125-2 | 0.5 | 1 | *(Aono et al., 2022)* |
| XDR | 120 |  | 0.13 | 0.25 | *(Zong et al., 2018)* |  |  | 420 | ≤0.125 - 1 | 0.5 | 0.5 | *(Yang et al., 2018)* |
|  | 88 | 0.03-2 | 0.12 | 0.5 | *(Zheng et al., 2021)* |  | XDR | 59 | 0.25-2 | 0.5 | 0.5 | *(Ahmed et al., 2013)* |
| MDR | 425 | 0.12-8 |  |  | *(Yao et al., 2021)* |  | Pre-XDR | 43 | 0.25-1 | 0.5 | 0.5 | *(Ahmed et al., 2013)* |
| All | 1452 | 0.06-32 |  |  | *(Guo et al., 2023)* |  |  | 117 | ≤0.125-1 | 0.5 | 1 | *(Alcalá et al., 2003)* |
| MDR | 156 |  | 0.25 | 1 | *(Guo et al., 2023)* |  | MDR | 33 | 0.06-1 | 0.5 | 0.5 | *(Ermertcan et al., 2009)* |
| Pre XDR | 93 |  | 0.5 | 1 | *(Guo et al., 2023)* |  | All | 59 | 0.125-64 | 1 | 32 | *(Sood et al., 2005)* |
| XDR | 27 |  | 1 | 32 | *(Guo et al., 2023)* |  | MDR | 16 | 0.125-64 | 4 | 64 | *(Sood et al., 2005)* |
| Drug-resistant | 39 | 0.125-0.5 | 0.125 | 0.25 | *(An et al., 2023)* |  | Resistant to either INH or RIF | 33 | 0.125-8 | 1 | 1 | *(Sood et al., 2005)* |
| Resistant to INH, RIF | 10 | 0.12-0.5 | 0.5 | 0.5 | *(Tato et al., 2006)* |  | MDR | 153 | ＜0.06-0.5 | 0.25 | 0.25 | *(Cavanaugh et al., 2017)* |
| Resistant to INH | 3 | 0.12-0.5 |  |  | *(Tato et al., 2006)* |  | MDR | 39 |  | 4 | 8 | *(Erturan & Uzun, 2005)* |
|  | 42 | 0.12-0.5 | 0.25 | 0.5 | *(Tato et al., 2006)* |  |  | 22 |  | 0.5 | 1 | *(Shoen et al., 2018)* |
| non-MDR | 34 | 0.06-1 | 0.5 | 0.5 | *(Ermertcan et al., 2009)* |  |  | 67 | 0.25-4 | 1 | 2 | *(Vera-Cabrera et al., 2005)* |
|  |  |  |  |  |  |  |  |  |  |  |  |  |
|  |  |  |  |  |  |  |  |  |  |  |  |  |
| **Organism** | **N** | **MIC range** | **MIC_50_** | **MIC_90_** | **Reference** |  | **Organism** | **N** | **MIC range** | **MIC_50_** | **MIC_90_** | **Reference** |
| **RGM** *M. abscessus* | 21 | 16-64 | 64 | 64 | *(Zhao et al., 2015)* |  | M. abscessus subsp. bolletii | 93 |  | 16 | ＞16 | *(Hunkins et al., 2023)* |
|  | 47 |  | 8 | ＞32 | *(Araj et al., 2019)* |  |  | 25 | 4-＞128 | 8 | 16 | *(Nie et al., 2014)* |
| *M. abscessus complex* | 20 | 2-128 |  | 16 | *(Shen et al., 2018)* |  |  | 1 | 32 |  |  | *(Lee et al., 2017)* |
|  | 35 | 0.5-32 | 16 | 16 | *(Zhang et al., 2022)* |  |  | 5 | 8-＞32 | 32 | ＞32 | *(Tang et al., 2018)* |
|  | 24 |  | 16 | 32 | *(He et al., 2022)* |  | M. abscessus subsp. massiliense | 20 | 0.5-8 | 2 | 4 | *(Zhang et al., 2017)* |
|  | 37 | 1-64 | 16 | 32 | *(Marfil et al., 2022)* |  |  | 21 | 1-32 | 4 | 8 | *(Gao et al., 2023)* |
|  | 65 | 1-32 | 4 | 16 | *(Gao et al., 2023)* |  |  | 38 | 2-＞32 | 32 | ＞32 | *(Lee et al., 2017)* |
| M. abscessus subsp. abscessus | 148 | 1-64 | 8 | 32 | *(Guo et al., 2021)* |  |  | 46 | 0.5-64 | 8 | 32 | *(Guo et al., 2021)* |
|  | 45 | 1-32 | 4 | 8 | *(Nie et al., 2014)* |  |  | 50 | 0.5-16 | 4 | 8 | *(Kim et al., 2021)* |
|  | 20 | 0.5-32 | 2 | 16 | *(Zhang et al., 2017)* |  |  | 18 | 1-16 | 2 | 8 | *(Kim et al., 2021)* |
|  | 44 | 1-16 | 8 | 16 | *(Gao et al., 2023)* |  |  | 12 | 0.5-32 | 8 | 32 | *(Brown-Elliott & Wallace Jr, 2017)* |
|  | 28 | 2-＞32 | ＞32 |  | *(Lee et al., 2017)* |  |  | 10 | 2-16 | 8 | 16 | *(Brown-Elliott et al., 2018)* |
|  | 30 | 2-128 | 16 | 64 | *(Heidarieh et al., 2016)* |  |  | 754 |  | ＞16 | ＞16 | *(Hunkins et al., 2023)* |
|  | 47 |  | 8 | ＞32 | *(Ruth et al., 2020)* |  |  | 82 | 0.5-＞32 | 8 | ＞32 | *(Tang et al., 2018)* |
|  | 47 | 0.5-64 | 4 | 8 | *(Kim et al., 2021)* |  | M. chelonae complex | 17 | 1-32 | 8 | 16 | *(Vera-Cabrera et al., 2006)* |
|  | 12 | 0.25-32 | 1 | 4 | *(Kim et al., 2021)* |  | M. chelonae | 2 | 8-16 |  |  | *(Ruth et al., 2020)* |
|  | 81 | 0.12-128 | 16 | 32 | *(Brown-Elliott & Wallace Jr, 2017)* |  |  | 22 | 2-16 | 8 | 16 | *(Brown-Elliott & Wallace Jr, 2017)* |
|  | 4 | 4-16 | 4 | 16 | *(Senol et al., 2022)* |  |  | 11 | 2-16 | 4 | 8 | *(Zhao et al., 2015)* |
|  | 14 | 8-64 | 64 | 64 | *(Vera-Cabrera et al., 2006)* |  |  | 1 | 4 |  |  | *(Zhang et al., 2022)* |
|  | 33 | 2-32 | 8 | 16 | *(Brown-Elliott et al., 2018)* |  |  | 10 | 2-16 | 8 | 16 | *(Brown-Elliott et al., 2018)* |
|  | 1344 |  | ＞16 | ＞16 | *(Hunkins et al., 2023)* |  |  | 526 |  | 16 | ＞16 | *(Hunkins et al., 2023)* |
|  | 43 | 0.0625＞32 | 8 | ＞32 | *(Tang et al., 2018)* |  |  | 39 | 2-128 | 16 | 64 | *(Heidarieh et al., 2016)* |
|  | 4 | 4-16 | 4 | 16 | *(Senol et al., 2022)* |  |  | 1 | 4 |  |  | *(Zhang et al., 2022)* |
| **Organism** | **N** | **MIC range** | **MIC_50_** | **MIC_90_** | **Reference** |  | **Organism** | **N** | **MIC range** | **MIC_50_** | **MIC_90_** | **Reference** |
|  | 2 | 8-16 |  |  | *(Araj et al., 2019)* |  |  | 40 | 4-＞128 | 64 | 128 | *(Zhang et al., 2022)* |
| M. fortuitum | 21 | 1-32 | 16 | 32 | *(Zhang et al., 2022)* |  |  | 43 |  | 32 | ＞64 | *(He et al., 2022)* |
|  | 24 | 0.5-8 | 4 | 8 | *(Zhao et al., 2015)* |  |  | 10 | 8-64 | 32 | 64 | *(Brown-Elliott et al., 2018)* |
|  | 53 | 0.0625-64 | 64 | 64 | *(Zheng et al., 2017)* |  |  | 189 | ≤2-＞32 | 32 | 64 | *(Brown-Elliott et al., 2003)* |
|  | 85 | 0.25-64 | 2 | 32 | *(Heidarieh et al., 2016)* |  |  | 100 | 2-128 | 32 | 64 | *(Brown-Elliott & Wallace Jr, 2017)* |
|  | 20 | 1-8 | 2 | 4 | *(Brown-Elliott & Wallace Jr, 2017)* |  | M.intracellulare | 17 | 8-64 | 8 | 16 | *(Zhao et al., 2015)* |
|  | 17 | 8-32 |  | 16 | *(Shen et al., 2018)* |  |  | 19 | 8-＞64 | 32 | 64 | *(Brown-Elliott et al., 2018)* |
|  | 21 | 1-32 | 16 | 32 | *(Zhang et al., 2022)* |  |  | 16 |  | 16 | 32 | *(Litvinov et al., 2018)* |
|  | 2 | 2 |  |  | *(Ruth et al., 2020)* |  |  | 685 |  | 32 | 64 | *(Cho et al., 2018)* |
|  | 1 | 32 |  |  | *(Senol et al., 2022)* |  |  | 16 | ≤0.063-64 | 32 | 64 | *(Kim et al., 2021)* |
|  | 564 |  | 16 | ＞16 | *(Hunkins et al., 2023)* |  |  |  | 16-32 |  |  | *(Araj et al., 2019)* |
|  | 2 | 2 |  |  | *(Araj et al., 2019)* |  |  | 188 | 0.5-32 | 8 | 16 | *(Zhang et al., 2015)* |
| **SGM**  M. avium | 31 | 8-64 | 32 | 32 | *(Zhao et al., 2015)* |  |  | 2 | 16-32 |  |  | *(Ruth et al., 2020)* |
|  | 65 | 0.0625-64 | 0.5 | 4 | *(Zhang et al., 2015)* |  |  | 75 | 4-64 | 32 | ＞64 | *(Huang et al., 2018)* |
|  | 51 |  | 16 | 32 | *(Ruth et al., 2020)* |  |  | 45 | 2-64 | 32 | 64 | *(Kim et al., 2021)* |
|  | 161 |  | 32 | 64 | *(Litvinov et al., 2018)* |  |  | 13 | 1-32 | 4 | 16 | *(Senol et al., 2022)* |
|  | 885 |  | 16 | 64 | *(Cho et al., 2018)* |  | M. gordonae | 2 | 4-16 |  |  | *(Zhang et al., 2022)* |
|  | 12 | 2-64 | 32 | 64 | *(Brown-Elliott et al., 2018)* |  |  | 1 | 0.5 |  |  | *(Senol et al., 2022)* |
|  | 52 | 0.5-＞64 | 64 | 64 | *(Kim et al., 2021)* |  |  | 21 | ≤0.5-16 | ≤2 | 4 | *(Brown-Elliott et al., 2003)* |
|  | 8 | 16-64 | 32 | 64 | *(Huang et al., 2018)* |  | M. kansasii | 26 | 0.5-1 | 1 | 1 | *(Zhao et al., 2015)* |
|  | 6 | 2-32 | 16 | 32 | *(Senol et al., 2022)* |  |  | 31 | 1-64 | 2 | 4 | *(Liu et al., 2021)* |
|  | 10 | 2-64 | 32 | 32 | *(Kim et al., 2021)* |  |  | 18 | 2-64 | 8 | 32 | *(Zhang et al., 2022)* |
| M. avium complex | 13 | 2-32 | 32 | 32 | *(Vera-Cabrera et al., 2006)* |  |  | 112 |  | 4 | 32 | *(Litvinov et al., 2018)* |

| **Organism** | **N** | **MIC range** | **MIC_50_** | **MIC_90_** | **Reference** |  | **Organism** | **N** | **MIC range** | **MIC_50_** | **MIC_90_** | **Reference** |
| --- | --- | --- | --- | --- | --- | --- | --- | --- | --- | --- | --- | --- |
|  | 8 | ≤1-4 | ≤1 | 4 | *(Brown-Elliott et al., 2018)* |  |  | 18 | ≤1-2 | ≤1 | 2 | *(Wang et al., 2022)* |
|  | 19 | ≤0.5-≤2 | ≤2 | ≤2 | *(Brown-Elliott et al., 2003)* |  |  | 21 | 1-4 | 2 | 2 | *(Brown-Elliott & Wallace Jr, 2017)* |
|  | 7 | 0.5-2 | 2 |  | *(Brown-Elliott & Wallace Jr, 2017)* |  |  | 1 | ≤1 |  |  | *(Wei et al., 2021)* |
|  | 18 | 2-64 | 8 | 32 | *(Zhang et al., 2022)* |  |  | 31 | 0.12-2 | 0.5 | 1 | *(Vera-Cabrera et al., 2006)* |
|  | 14 |  | 2 | 2 | *(He et al., 2022)* |  | N. cyriacigeorgica | 2 | 2 |  |  | *(Goodlet et al., 2021)* |
|  | 6 | 0.5-1 | 0.5 | 1 | *(Senol et al., 2022)* |  |  | 27 | 2-4 | 4 | 4 | *(Toyokawa et al., 2021)* |
|  | 40 | 0.125-2 | 0.125 | 1 | *(Heidarieh et al., 2016)* |  |  | 6 | ≤1-16 |  |  | *(Yi et al., 2019)* |
| M. xenopi | 74 |  | 4 | 16 | *(Litvinov et al., 2018)* |  |  | 126 | ≤1-4 | ≤1 | 2 | *(Wang et al., 2022)* |
| M. marinum | 10 | <0.5-16 |  |  | *(Zhang et al., 2022)* |  |  | 29 | 0.25-2 | 2 | 4 | *(Brown-Elliott & Wallace Jr, 2017)* |
|  | 9 | ≤1-2 | 2 | 2 | *(Brown-Elliott et al., 2018)* |  |  | 2 | 0.094-1 |  |  | *(Mazzaferri et al., 2018)* |
|  | 47 | 1-2 | ≤2 | 2 | *(Brown-Elliott et al., 2003)* |  |  | 33 | ≤1-4 | 2 | 2 | *(Wei et al., 2021)* |
|  | 7 | 1-4 | 1 |  | *(Brown-Elliott & Wallace Jr, 2017)* |  |  | 13 | 1-2 |  |  | *(Wei et al., 2017)* |
|  | 10 | <0.125-2 |  |  | *(Zhang et al., 2022)* |  |  | 16 | 2-4 | 2 | 4 | *(Lao et al., 2022)* |
| **Nocardia** N. abscessus | 3 | 0.19-0.25 |  |  | *(Mazzaferri et al., 2018)* |  |  | 25 |  | 2 | 2 | *(Kuo et al., 2022)* |
|  | 5 | ≤1-2 | ≤1 | 2 | *(Wei et al., 2021)* |  | N. farcinica | 7 | 2-4 |  |  | *(Goodlet et al., 2021)* |
|  | 2 | 0.5-2 |  |  | *(Wei et al., 2017)* |  |  | 8 | ≤1-2 |  |  | *(Yi et al., 2019)* |
|  | 1 |  |  |  | *(Harris et al., 2021)* |  |  | 18 |  | 2 | 4 | *(Kuo et al., 2022)* |
| N. abscessus complex | 54 | ≤1-2 | ≤1 | ≤1 | *(Wang et al., 2022)* |  |  | 176 | ≤1-4 | 2 | 2 | *(Wang et al., 2022)* |
|  | 18 | 0.25-4 | 2 | 4 | *(Toyokawa et al., 2021)* |  |  | 17 | 1-4 | 2 | 4 | *(Brown-Elliott & Wallace Jr, 2017)* |
| N. brasiliensis | 13 |  | 2 | 4 | *(Kuo et al., 2022)* |  |  | 3 | 0.064-2 |  |  | *(Mazzaferri et al., 2018)* |
|  | 28 | 1-4 | 2 | 2 | *(Lao et al., 2022)* |  |  | 24 | 2-4 | 2 | 4 | *(Lao et al., 2022)* |
|  | 14 | 4-8 | 4 | 8 | *(Toyokawa et al., 2021)* |  |  | 20 | ≤1-4 | 2 | 4 | *(Wei et al., 2021)* |
|  | 2 | 2-4 |  |  | *(Yi et al., 2019)* |  |  | 36 |  | 2 | 2 | *(Li et al., 2022)* |
| **Organism** | **N** | **MIC range** | **MIC_50_** | **MIC_90_** | **Reference** |  | **Organism** | **N** | **MIC range** | **MIC_50_** | **MIC_90_** | **Reference** |
|  | 1 | 0.25 |  |  | *(Mazzaferri et al., 2018)* |  |  | 23 | ≤0.25-4 | 2 | 4 | *(Toyokawa et al., 2021)* |
|  | 6 | 1-4 |  |  | *(Wei et al., 2017)* |  | N. veterana | 2 | 1-16 |  |  | *(Lao et al., 2022)* |
| N. nova | 1 | ≤1 |  |  | *(Yi et al., 2019)* |  | N. otitidiscaviarium | 2 | ≤1-2 |  |  | *(Yi et al., 2019)* |
|  | 6 | 2 | 2 | 2 | *(Lao et al., 2022)* |  |  | 11 | 1-8 | 4 | 4 | *(Toyokawa et al., 2021)* |
|  | 1 | ≤1 |  |  | *(Wei et al., 2021)* |  |  | 26 | ≤1-2 | ≤1 | 2 | *(Wang et al., 2022)* |
|  | 1 | 2 |  |  | *(Wei et al., 2017)* |  |  | 7 | ≤1-4 | ≤1 | 4 | *(Wei et al., 2021)* |
| N. nova complex | 11 | ≤1-2 | ≤1 | 2 | *(Wang et al., 2022)* |  |  | 4 | 2-4 | 2 | 4 | *(Lao et al., 2022)* |
|  | 13 | 0.25-2 | 1 | 2 | *(Brown-Elliott & Wallace Jr, 2017)* |  |  | 1 | 0.5 |  |  | *(Wei et al., 2017)* |

Abbreviations: N, number of strains; MIC, minimum inhibitory concentration; MDR, multidrug-resistant; XDR, extensively drug-resistant. MIC_90_, minimum inhibitory concentration required to inhibit growth of 90% of isolates；MIC_50_, minimum inhibitory concentration required to inhibit growth of 50% of isolates；

**REFERENCES**

**Ahmed I, Jabeen K, Inayat R, Hasan R**. **2013**. Susceptibility testing of extensively drug-resistant and pre-extensively drug-resistant Mycobacterium tuberculosis against levofloxacin, linezolid, and amoxicillin-clavulanate. *Antimicrobial agents and chemotherapy* **57(6)**:2522-2525 DOI 10.1128/AAC.02020-12.

**Alcalá L, Ruiz-Serrano MJ, Pérez-Fernández Turégano C, García De Viedma D, Díaz-Infantes M, Marín-Arriaza M, Bouza E**. **2003**. In vitro activities of linezolid against clinical isolates of Mycobacterium tuberculosis that are susceptible or resistant to first-line antituberculous drugs. *Antimicrobial agents and chemotherapy* **47(1)**:416-417 DOI 10.1128/AAC.47.1.416-417.2003.

**An H, Sun W, Liu X, Wang T, Qiao J, Liang J**. **2023**. In vitro activities of contezolid (MRX-I) against drug-sensitive and drug-resistant Mycobacterium tuberculosis. *Microbiology spectrum* **11(5)**:e0462722 DOI 10.1128/spectrum.04627-22.

**Aono A, Murase Y, Chikamatsu K, Igarashi Y, Shimomura Y, Hosoya M, Osugi A, Morishige Y, Takaki A, Yamada H, Mitarai S**. **2022**. In vitro activity of tedizolid and linezolid against multidrug-resistant Mycobacterium tuberculosis: a comparative study using microdilution broth assay and genomics. *Diagnostic microbiology and infectious disease* **103(3)**:115714 DOI 10.1016/j.diagmicrobio.2022.115714.

**Araj GF, Baba OZ, Itani LY, Avedissian AZ, Sobh GM**. **2019**. Non-tuberculous mycobacteria profiles and their anti-mycobacterial resistance at a major medical center in Lebanon. *Journal of infection in developing countries* **13(7)**:612-618 DOI 10.3855/jidc.11028.

**Brown-Elliott BA, Crist CJ, Mann LB, Wilson RW, Wallace RJ Jr**. **2003**. In vitro activity of linezolid against slowly growing nontuberculous Mycobacteria. *Antimicrobial agents and chemotherapy* **47(5)**:1736-1738 DOI 10.1128/AAC.47.5.1736-1738.2003.

**Brown-Elliott BA, Rubio A, Wallace RJ Jr**. **2018**. In Vitro Susceptibility Testing of a Novel Benzimidazole, SPR719, against Nontuberculous Mycobacteria. *Antimicrobial agents and chemotherapy* **62(11)**:e01503-01518 DOI 10.1128/AAC.01503-18.

**Brown-Elliott BA, Wallace RJ Jr**. **2017**. In Vitro Susceptibility Testing of Tedizolid against Isolates of Nocardia. *Antimicrobial agents and chemotherapy* **61(12)**:e01537-01517 DOI 10.1128/AAC.01537-17.

**Brown-Elliott BA, Wallace RJ Jr**. **2017**. In Vitro Susceptibility Testing of Tedizolid against Nontuberculous Mycobacteria. *Journal of clinical microbiology* **55(6)**:1747-1754 DOI 10.1128/JCM.00274-17.

**Cavanaugh JS, Jou R, Wu MH, Dalton T, Kurbatova E, Ershova J, Cegielski JP**. **2017**. Susceptibilities of MDR Mycobacterium tuberculosis isolates to unconventional drugs compared with their reported pharmacokinetic/pharmacodynamic parameters. *The Journal of antimicrobial chemotherapy* **72(6)**:1678-1687 DOI 10.1093/jac/dkx022.

**Cho EH, Huh HJ, Song DJ, Moon SM, Lee SH, Shin SY, Kim CK, Ki CS, Koh WJ, Lee NY**. **2018**. Differences in drug susceptibility pattern between Mycobacterium avium and Mycobacterium intracellulare isolated in respiratory specimens. *Journal of infection and chemotherapy : official journal of the Japan Society of Chemotherapy* **24(4)**:315-318 DOI 10.1016/j.jiac.2017.10.022.

**Ermertcan S, Hosgor-Limoncu M, Erac B, Tasli H, Cavusoglu C, Bozkurt H**. **2009**. In vitro activity of linezolid against Mycobacterium tuberculosis strains isoalted from Western Turkey. *Japanese journal of infectious diseases* **62(5)**:384-385

**Erturan Z, Uzun M**. **2005**. In vitro activity of linezolid against multidrug-resistant Mycobacterium tuberculosis isolates. *International journal of antimicrobial agents* **26(1)**:78-80 DOI 10.1016/j.ijantimicag.2005.03.006.

**Gao T, Yao C, Shang Y, Su R, Zhang X, Ren W, Li S, Shu W, Pang Y, Li Q**. **2023**. Antimicrobial Effect of Oxazolidinones and Its Synergistic Effect with Bedaquiline Against Mycobacterium abscessus Complex. *Infection and drug resistance* **16**:279-287 DOI 10.2147/IDR.S395750.

**Goodlet KJ, Tokman S, Nasar A, Cherrier L, Walia R, Nailor MD**. **2021**. Nocardia prophylaxis, treatment, and outcomes of infection in lung transplant recipients: A matched case-control study. *Transplant Infectious Disease* **23(2)**:e13478 DOI 10.1111/tid.13478.

**Guo Q, Xu L, Tan F, Zhang Y, Fan J, Wang X, Zhang Z, Li B, Chu H**. **2021**. A Novel Oxazolidinone, Contezolid (MRX-I), Expresses Anti-Mycobacterium abscessus Activity In Vitro. *Antimicrobial agents and chemotherapy* **65(11)**:e0088921 DOI 10.1128/AAC.00889-21.

**Guo S, Wang B, Fu L, Chen X, Zhang W, Huang H, Lu Y**. **2021**. In Vitro and In Vivo Activity of Oxazolidinone Candidate OTB-658 against Mycobacterium tuberculosis. *Antimicrobial agents and chemotherapy* **65(11)**:e0097421 DOI 10.1128/AAC.00974-21.

**Guo Y, Yang J, Wang W, Wu X, Wan B, Wang H, Sha W, Yu F**. **2023**. Bedaquiline, Delamanid, Linezolid, Clofazimine, and Capreomycin MIC Distributions for Drug Resistance Mycobacterium tuberculosis in Shanghai, China. *Infection and drug resistance* **16**:7587-7595 DOI 10.2147/IDR.S440711.

**Harris DM, Dumitrascu AG, Chirila RM, Omer M, Stancampiano FF, Hata DJ, Meza Villegas DM, Heckman MG, Cochuyt JJ, Alvarez S**. **2021**. Invasive Nocardiosis in Transplant and Nontransplant Patients: 20-Year Experience in a Tertiary Care Center. *Mayo Clinic proceedings. Innovations, quality & outcomes* **5(2)**:298-307 DOI 10.1016/j.mayocpiqo.2020.10.009.

**He G, Wu L, Zheng Q, Jiang X**. **2022**. Antimicrobial susceptibility and minimum inhibitory concentration distribution of common clinically relevant non-tuberculous mycobacterial isolates from the respiratory tract. *Annals of medicine* **54(1)**:2500-2510 DOI 10.1080/07853890.2022.2121984.

**Heidarieh P, Mirsaeidi M, Hashemzadeh M, Feizabadi MM, Bostanabad SZ, Nobar MG, Hashemi Shahraki A**. **2016**. In Vitro Antimicrobial Susceptibility of Nontuberculous Mycobacteria in Iran. *Microbial drug resistance (Larchmont, N.Y.)* **22(2)**:172-178 DOI 10.1089/mdr.2015.0134.

**Huang CC, Wu MF, Chen HC, Huang WC**. **2018**. In vitro activity of aminoglycosides, clofazimine, d-cycloserine and dapsone against 83 Mycobacterium avium complex clinical isolates. *Journal of microbiology, immunology, and infection = Wei mian yu gan ran za zhi* **51(5)**:636-643 DOI 10.1016/j.jmii.2017.05.001.

**Huang TS, Liu YC, Sy CL, Chen YS, Tu HZ, Chen BC**. **2008**. In vitro activities of linezolid against clinical isolates of Mycobacterium tuberculosis complex isolated in Taiwan over 10 years. *Antimicrobial agents and chemotherapy* **52(6)**:2226-2227 DOI 10.1128/AAC.00414-07.

**Hunkins JJ, de-Moura VC, Eddy JJ, Daley CL, Khare R**. **2023**. In vitro susceptibility patterns for rapidly growing nontuberculous mycobacteria in the United States. *Diagnostic microbiology and infectious disease* **105(3)**:115882 DOI 10.1016/j.diagmicrobio.2022.115882.

**Kardan-Yamchi J, Kazemian H, Battaglia S, Abtahi H, Foroushani AR, Hamzelou G, Cirillo DM, Ghodousi A, Feizabadi MM**. **2020**. Whole Genome Sequencing Results Associated with Minimum Inhibitory Concentrations of 14 Anti-Tuberculosis Drugs among Rifampicin-Resistant Isolates of Mycobacterium Tuberculosis from Iran. *Journal of clinical medicine* **9(2)**:465 DOI 10.3390/jcm9020465.

**Kazemian H, Haeili M, Kardan Yamchi J, Rezaei F, Gizaw Feyisa S, Zahednamazi F, Mohajeri P, Zaker Bostanabd S, Hashemi Shahraki A, Imani Fooladi AA, Feizabadi MM**. **2015**. Antimycobacterial activity of linezolid against multidrug-resistant and extensively drug-resistant strains of Mycobacterium tuberculosis in Iran. *International journal of antimicrobial agents* **45(6)**:668-670 DOI 10.1016/j.ijantimicag.2015.02.004.

**Kim DH, Kim SY, Koh WJ, Jhun BW**. **2021**. In Vitro Activity of Oxazolidinone against Nontuberculous Mycobacteria, Including Macrolide-Resistant Clinical Isolates. *Antimicrobial agents and chemotherapy* **65(7)**:e0230620 DOI 10.1128/AAC.02306-20.

**Kuo SF, Chen FJ, Lan IC, Chien CC, Lee CH**. **2022**. Epidemiology of Nocardia Species at a Tertiary Hospital in Southern Taiwan, 2012 to 2020: MLSA Phylogeny and Antimicrobial Susceptibility. *Antibiotics (Basel, Switzerland)* **11(10)**:1438 DOI 10.3390/antibiotics11101438.

**Lao CK, Tseng MC, Chiu CH, Chen NY, Chen CH, Chung WH, Liu TP, Lu JJ, Lai HC, Yang LY, Lee CH, Wu TS**. **2022**. Clinical manifestations and antimicrobial susceptibility of Nocardia species at a tertiary hospital in Taiwan, 2011-2020. *Journal of the Formosan Medical Association = Taiwan yi zhi* **121(10)**:2109-2122 DOI 10.1016/j.jfma.2022.06.011.

**Lee MC, Sun PL, Wu TL, Wang LH, Yang CH, Chung WH, Kuo AJ, Liu TP, Lu JJ, Chiu CH, Lai HC, Chen NY, Yang JH, Wu TS**. **2017**. Antimicrobial resistance in Mycobacterium abscessus complex isolated from patients with skin and soft tissue infections at a tertiary teaching hospital in Taiwan. *The Journal of antimicrobial chemotherapy* **72(10)**:2782-2786 DOI 10.1093/jac/dkx212.

**Li J, Shen H, Yu T, Tao XY, Hu YM, Wang HC, Zou MX**. **2022**. Isolation and Characterization of Nocardia Species from Pulmonary Nocardiosis in a Tertiary Hospital in China. *Japanese journal of infectious diseases* **75(1)**:31-35 DOI 10.7883/yoken.JJID.2020.1096.

**Litvinov V, Makarova M, Galkina K, Khachaturiants E, Krasnova M, Guntupova L, Safonova S**. **2018**. Drug susceptibility testing of slowly growing non-tuberculous mycobacteria using slomyco test-system. *PloS one* **13(9)**:e0203108 DOI 10.1371/journal.pone.0203108.

**Liu CF, Song YM, He WC, Liu DX, He P, Bao JJ, Wang XY, Li YM, Zhao YL**. **2021**. Nontuberculous mycobacteria in China: incidence and antimicrobial resistance spectrum from a nationwide survey. *Infectious diseases of poverty* **10(1)**:59 DOI 10.1186/s40249-021-00844-1.

**Marfil E, Ruiz P, Martínez-Martínez L, Causse M**. **2022**. Comparative study of in vitro activity of tedizolid and linezolid against Mycobacterium avium complex. *Journal of global antimicrobial resistance* **30**:395-398 DOI 10.1016/j.jgar.2022.07.012.

**Mazzaferri F, Cordioli M, Segato E, Adami I, Maccacaro L, Sette P, Cazzadori A, Concia E, Azzini AM**. **2018**. Nocardia infection over 5 years (2011-2015) in an Italian tertiary care hospital. *The new microbiologica* **41(2)**:136-140

**Nie W, Duan H, Huang H, Lu Y, Bi D, Chu N**. **2014**. Species identification of Mycobacterium abscessus subsp. abscessus and Mycobacterium abscessus subsp. bolletii using rpoB and hsp65, and susceptibility testing to eight antibiotics. *International journal of infectious diseases : IJID : official publication of the International Society for Infectious Diseases* **25**:170-174 DOI 10.1016/j.ijid.2014.02.014.

**Pang Y, Zong Z, Huo F, Jing W, Ma Y, Dong L, Li Y, Zhao L, Fu Y, Huang H**. **2017**. In Vitro Drug Susceptibility of Bedaquiline, Delamanid, Linezolid, Clofazimine, Moxifloxacin, and Gatifloxacin against Extensively Drug-Resistant Tuberculosis in Beijing, China. *Antimicrobial agents and chemotherapy* **61(10)**:e00900-00917 DOI 10.1128/AAC.00900-17.

**Ruth MM, Koeken V, Pennings LJ, Svensson EM, Wertheim H, Hoefsloot W, van Ingen J**. **2020**. Is there a role for tedizolid in the treatment of non-tuberculous mycobacterial disease. *The Journal of antimicrobial chemotherapy* **75(3)**:609-617 DOI 10.1093/jac/dkz511.

**Senol G, Bicmen C, Gunduz A, Dereli S, Erbaycu A**. **2022**. Evaluation of antimicrobial susceptibilities of non-tuberculous mycobacteria against linezolid and tigecycline. *Indian journal of medical microbiology* **40(3)**:446-448 DOI 10.1016/j.ijmmb.2022.03.012.

**Shen Y, Wang X, Jin J, Wu J, Zhang X, Chen J, Zhang W**. **2018**. In Vitro Susceptibility of Mycobacterium abscessus and Mycobacterium fortuitum Isolates to 30 Antibiotics. *BioMed research international* **2018**:4902941 DOI 10.1155/2018/4902941.

**Shoen C, DeStefano M, Hafkin B, Cynamon M**. **2018**. In Vitro and In Vivo Activities of Contezolid (MRX-I) against Mycobacterium tuberculosis. *Antimicrobial agents and chemotherapy* **62(8)**:e00493-00418 DOI 10.1128/AAC.00493-18.

**Singh K, Sharma S, Banerjee T, Gupta A, Anupurba S**. **2022**. Mutation detection and minimum inhibitory concentration determination against linezolid and clofazimine in confirmed XDR-TB clinical isolates. *BMC microbiology* **22(1)**:236 DOI 10.1186/s12866-022-02622-x.

**Sood R, Rao M, Singhal S, Rattan A**. **2005**. Activity of RBx 7644 and RBx 8700, new investigational oxazolidinones, against Mycobacterium tuberculosis infected murine macrophages. *International journal of antimicrobial agents* **25(6)**:464-468 DOI 10.1016/j.ijantimicag.2005.01.021.

**Tang YW, Cheng B, Yeoh SF, Lin R, Teo J**. **2018**. Tedizolid Activity Against Clinical Mycobacterium abscessus Complex Isolates-An in vitro Characterization Study. *Frontiers in microbiology* **9**:2095 DOI 10.3389/fmicb.2018.02095.

**Tato M, de la Pedrosa EG, Cantón R, Gómez-García I, Fortún J, Martín-Davila P, Baquero F, Gomez-Mampaso E**. **2006**. In vitro activity of linezolid against Mycobacterium tuberculosis complex, including multidrug-resistant Mycobacterium bovis isolates. *International journal of antimicrobial agents* **28(1)**:75-78 DOI 10.1016/j.ijantimicag.2006.02.011.

**Toyokawa M, Ohana N, Ueda A, Imai M, Tanno D, Honda M, Takano Y, Ohashi K, Saito K, Shimura H**. **2021**. Identification and antimicrobial susceptibility profiles of Nocardia species clinically isolated in Japan. *Scientific reports* **11(1)**:16742 DOI 10.1038/s41598-021-95870-2.

**Vera-Cabrera L, Brown-Elliott BA, Wallace RJ Jr, Ocampo-Candiani J, Welsh O, Choi SH, Molina-Torres CA**. **2006**. In vitro activities of the novel oxazolidinones DA-7867 and DA-7157 against rapidly and slowly growing mycobacteria. *Antimicrobial agents and chemotherapy* **50(12)**:4027-4029 DOI 10.1128/AAC.00763-06.

**Vera-Cabrera L, Castro-Garza J, Rendon A, Ocampo-Candiani J, Welsh O, Choi SH, Blackwood K, Molina-Torres C**. **2005**. In vitro susceptibility of Mycobacterium tuberculosis clinical isolates to garenoxacin and DA-7867. *Antimicrobial agents and chemotherapy* **49(10)**:4351-4353 DOI 10.1128/AAC.49.10.4351-4353.2005.

**Vera-Cabrera L, Gonzalez E, Rendon A, Ocampo-Candiani J, Welsh O, Velazquez-Moreno VM, Choi SH, Molina-Torres C**. **2006**. In vitro activities of DA-7157 and DA-7218 against Mycobacterium tuberculosis and Nocardia brasiliensis. *Antimicrobial agents and chemotherapy* **50(9)**:3170-3172 DOI 10.1128/AAC.00571-06.

**Wang C, Wang G, Huo F, Xue Y, Jia J, Dong L, Zhao L, Wang F, Huang H, Duan H**. **2022**. Novel oxazolidinones harbor potent in vitro activity against the clinical isolates of multidrug-resistant Mycobacterium tuberculosis in China. *Frontiers in medicine* **9**:1067516 DOI 10.3389/fmed.2022.1067516.

**Wang H, Zhu Y, Cui Q, Wu W, Li G, Chen D, Xiang L, Qu J, Shi D, Lu B**. **2022**. Epidemiology and Antimicrobial Resistance Profiles of the Nocardia Species in China, 2009 to 2021. *Microbiology spectrum* **10(2)**:e0156021 DOI 10.1128/spectrum.01560-21.

**Wei M, Wang P, Qu J, Li R, Liu Y, Gu L, Yang C**. **2017**. Identification and antimicrobial susceptibility of clinical Nocardia species in a tertiary hospital in China. *Journal of global antimicrobial resistance* **11**:183-187 DOI 10.1016/j.jgar.2017.08.011.

**Wei M, Xu X, Yang J, Wang P, Liu Y, Wang S, Yang C, Gu L**. **2021**. MLSA phylogeny and antimicrobial susceptibility of clinical Nocardia isolates: a multicenter retrospective study in China. *BMC microbiology* **21(1)**:342 DOI 10.1186/s12866-021-02412-x.

**Yang C, Lei H, Wang D, Meng X, He J, Tong A, Zhu L, Jiang Y, Dong M**. **2012**. In vitro activity of linezolid against clinical isolates of Mycobacterium tuberculosis, including multidrug-resistant and extensively drug-resistant strains from Beijing, China. *Japanese journal of infectious diseases* **65(3)**:240-242 DOI 10.7883/yoken.65.240.

**Yang JS, Kim KJ, Choi H, Lee SH**. **2018**. Delamanid, Bedaquiline, and Linezolid Minimum Inhibitory Concentration Distributions and Resistance-related Gene Mutations in Multidrug-resistant and Extensively Drug-resistant Tuberculosis in Korea. *Annals of laboratory medicine* **38(6)**:563-568 DOI 10.3343/alm.2018.38.6.563.

**Yao C, Guo H, Li Q, Zhang X, Shang Y, Li T, Wang Y, Xue Z, Wang L, Li L, Pang Y**. **2021**. Prevalence of extensively drug-resistant tuberculosis in a Chinese multidrug-resistant TB cohort after redefinition. *Antimicrobial resistance and infection control* **10(1)**:126 DOI 10.1186/s13756-021-00995-8.

**Yi M, Wang L, Xu W, Sheng L, Jiang L, Yang F, Cao Q, Wu J**. **2019**. Species Distribution And Antibiotic Susceptibility Of Nocardia Isolates From Yantai, China. *Infection and drug resistance* **12**:3653-3661 DOI 10.2147/IDR.S232098.

**Zhang H, Hua W, Lin S, Zhang Y, Chen X, Wang S, Chen J, Zhang W**. **2022**. In vitro Susceptibility of Nontuberculous Mycobacteria to Tedizolid. *Infection and drug resistance* **15**:4845-4852 DOI 10.2147/IDR.S362583.

**Zhang L, Pang Y, Yu X, Wang Y, Gao M, Huang H, Zhao Y**. **2014**. Linezolid in the treatment of extensively drug-resistant tuberculosis. *Infection* **42(4)**:705-711 DOI 10.1007/s15010-014-0632-2.

**Zhang Z, Lu J, Liu M, Wang Y, Zhao Y, Pang Y**. **2017**. In vitro activity of clarithromycin in combination with other antimicrobial agents against Mycobacterium abscessus and Mycobacterium massiliense. *International journal of antimicrobial agents* **49(3)**:383-386 DOI 10.1016/j.ijantimicag.2016.12.003.

**Zhang Z, Pang Y, Wang Y, Cohen C, Zhao Y, Liu C**. **2015**. Differences in risk factors and drug susceptibility between Mycobacterium avium and Mycobacterium intracellulare lung diseases in China. *International journal of antimicrobial agents* **45(5)**:491-495 DOI 10.1016/j.ijantimicag.2015.01.012.

**Zhang Z, Pang Y, Wang Y, Liu C, Zhao Y**. **2014**. Beijing genotype of Mycobacterium tuberculosis is significantly associated with linezolid resistance in multidrug-resistant and extensively drug-resistant tuberculosis in China. *International journal of antimicrobial agents* **43(3)**:231-235 DOI 10.1016/j.ijantimicag.2013.12.007.

**Zhao W, Jiang Y, Bao P, Li Y, Tang L, Zhou Y, Zhao Y**. **2015**. Evaluation of the Efficacy of Novel Oxazolidinone Analogues against Nontuberculous Mycobacteria In Vitro. *Japanese journal of infectious diseases* **68(6)**:520-522 DOI 10.7883/yoken.JJID.2014.498.

**Zheng H, He W, Jiao W, Xia H, Sun L, Wang S, Xiao J, Ou X, Zhao Y, Shen A**. **2021**. Molecular characterization of multidrug-resistant tuberculosis against levofloxacin, moxifloxacin, bedaquiline, linezolid, clofazimine, and delamanid in southwest of China. *BMC infectious diseases* **21(1)**:330 DOI 10.1186/s12879-021-06024-8.

**Zheng HW, Pang Y, He GX, Song YY, Zhao YL**. **2017**. Antimicrobial Susceptibility Testing and Molecular Characterization of Mycobacterium fortuitum Isolates in China. *Biomedical and environmental sciences : BES* **30(5)**:376-379 DOI 10.3967/bes2017.049.

**Zong Z, Jing W, Shi J, Wen S, Zhang T, Huo F, Shang Y, Liang Q, Huang H, Pang Y**. **2018**. Comparison of In Vitro Activity and MIC Distributions between the Novel Oxazolidinone Delpazolid and Linezolid against Multidrug-Resistant and Extensively Drug-Resistant Mycobacterium tuberculosis in China. *Antimicrobial agents and chemotherapy* **62(8)**:e00165-00118 DOI 10.1128/AAC.00165-18.
