# Supplementary material for: The in vitro antimicrobial activity of linezolid against unconventional pathogens
Source: PeerJ. 2025 Feb 12;13:e18825. doi: 10.7717/peerj.18825 (PMC11829633; doi:10.7717/peerj.18825)
Supplement: Supplemental Information 2 [file peerj-13-18825-s002.docx]

Table 2 In vitro activity of linezolid against Corynebacterium, Anaerobe and other pathogens (MIC, μg/mL).

| **Organism** | **N** | **MIC range** | **MIC_50_** | **MIC_90_** | **Reference** |  | **Organism** | **N** | **MIC range** | **MIC_50_** | **MIC_90_** | **Reference** |
| --- | --- | --- | --- | --- | --- | --- | --- | --- | --- | --- | --- | --- |
| ***Corynebacterium***  *Corynebacterium spp.* | 48 | 0.12-1 | 0.25 | 0.5 | *(Jones et al., 2002)* |  |  | 19 | ≤ 0.5 | ≤ 0.5 | ≤ 0.5 | *(Johnson et al., 2003)* |
|  | 34 |  | 0.25 | 0.5 | *(Mendes et al., 2015)* |  |  | 76 | ≤ 1 | ≤ 1 | ≤ 1 | *(Neemuchwala et al., 2018)* |
|  | 10 | 0.125-0.5 | 0.25 | 0.5 | *(Goldstein et al., 2004)* |  |  | 4 |  | 1 | 2 | *(Sun et al., 2022)* |
|  | 50 | 0.12-2 | 0.5 | 1 | *(Ract et al., 2017)* |  | *C. striatum* | 55 | ≤ 0.063-2 | 0.125 | 0.25 | *(Barberis et al., 2018)* |
|  | 20 | 1-8 | 2 | 8 | *(Goldstein et al., 2006)* |  |  | 30 | ≤ 0.12-1 | 0.25 | 0.5 | *(Gómez-Garcés et al., 2007)* |
| *C. amycolatum* | 35 | 0.063-1 | 0.25 | 1 | *(Barberis et al., 2018)* |  |  | 11 | 0.125-0.75 | 0.38 | 0.5 | *(Fernandez-Roblas et al., 2009)* |
|  | 33 | 0.064-0.25 | 0.19 | 0.25 | *(Fernandez-Roblas et al., 2009)* |  |  | 5 | 0.5 |  |  | *(Johnson et al., 2003)* |
|  | 58 | ≤ 0.1-2 | 0.2 | 0.2 | *(Sánchez Hernández et al., 2003)* |  |  | 124 | ≤ 0.5-0.5 | ≤ 0.5 | ≤ 0.5 | *(Abe et al., 2021)* |
|  | 60 | ≤ 0.12-0.5 | 0.25 | 0.25 | *(Gómez-Garcés et al., 2007)* |  |  | 931 | ≤ 1 | ≤ 1 | ≤ 1 | *(Neemuchwala et al., 2018)* |
|  | 20 | 0.25-0.5 | 0.5 | 0.5 | *(Goldstein et al., 2006)* |  |  | 410 | ≤ 2 | <0.5 | <0.5 | *(Wang et al., 2021)* |
|  | 10 | 0.25-0.5 | 0.5 | 0.5 | *(Goldstein et al., 2004)* |  |  | 15 |  | 0.5 | 0.5 | *(Sun et al., 2022)* |
|  | 5 | 0.4-4 |  |  | *(Johnson et al., 2003)* |  |  | 7 |  |  | 0.12-0.25 | *(Nhan et al., 2012)* |
|  | 1 | ≤ 0.5 |  |  | *(Abe et al., 2021)* |  | *C. urealyticum* | 40 | 0.015-256 | 0.75 | 1 | *(Chapartegui-González et al., 2020)* |
|  | 190 | ≤ 1 | ≤ 1 | ≤ 1 | *(Neemuchwala et al., 2018)* |  |  | 34 | 0.064-0.5 | 0.19 | 0.38 | *(Fernandez-Roblas et al., 2009)* |
|  | 5 |  | 1 | 1 | *(Sun et al., 2022)* |  |  | 10 | 0.25-1 | 0.5 | 0.5 | *(Gómez-Garcés et al., 2007)* |
| *C. jeikeium* | 25 | 0.064-0.38 | 0.25 | 0.5 | *(Fernandez-Roblas et al., 2009)* |  |  | 64 | 0.5-1 | 0.5 | 0.5 | *(Sánchez Hernández et al., 2003)* |
|  | 30 | ≤ 0.12-1 | 0.5 | 0.5 | *(Gómez-Garcés et al., 2007)* |  |  | 52 | ≤ 1 | ≤ 1 | ≤ 1 | *(Neemuchwala et al., 2018)* |
|  | 31 | 0.2-1 | 1 | 1 | *(Sánchez Hernández et al., 2003)* |  |  | 8 |  | 0.12 | 1 | *(Sun et al., 2022)* |
|  | 11 | 0.5-0.5 | 0.5 | 0.5 | *(Goldstein et al., 2004)* |  |  |  |  |  |  |  |
|  | 72 | 0.5-2 |  |  | *(Johnson et al., 2003)* |  |  |  |  |  |  |  |
| **Organism** | **N** | **MIC range** | **MIC_50_** | **MIC_90_** | **Reference** |  | **Organism** | **N** | **MIC range** | **MIC_50_** | **MIC_90_** | **Reference** |
| ***Anaerobes***  *Clostridium spp.* | 20 | ≤0.06-4 | 2 | 4 | *(Behra-Miellet et al., 2003)* |  | *Actinomyces spp.* | 22 | 0.5-8 | 0.5 | 0.5 | *(Citron et al., 2003)* |
|  | 38 | 0.25 -16 | 2 | 4 | *(Wybo et al., 2014)* |  | *Actinomyces israelii* | 13 | 0.25-16 | 0.5 | 16 | *(Goldstein et al., 2005)* |
|  | 25 | 0.25-4 | 1 | 4 | *(Citron et al., 2003)* |  |  | 13 | 0.25-16 | 0.5 | 16 | *(Goldstein et al., 2004)* |
|  | 10 | 1-4 | 2 | 4 | *(Mathur et al., 2011)* |  | *Propionibacterium spp.* | 52 | 0.12-2 | 0.5 | 1 | *(Ract et al., 2017)* |
|  | 4 | 2-4 |  |  | *(Lee et al., 2015)* |  |  | 13 | 0.25-0.5 | 0.5 | 0.5 | *(Behra-Miellet et al., 2003)* |
|  | 20 | 1-8 | 2 | 8 | *(Goldstein et al., 2006)* |  |  | 15 | 0.25-1 | 5 | 1 | *(Citron et al., 2003)* |
| *Clostridium difficile* | 114 | 0.125-8 | 2 | 4 | *(Rashid et al., 2014)* |  | *Propionibacterium acnes* | 10 | 0.25-0.5 | 0.5 | 0.5 | *(Mathur et al., 2011)* |
|  | 94 | 0.5-4 | 2 | 2 | *(Phillips et al., 2003)* |  |  | 10 | 0.25-0.5 | 0.5 | 0.5 | *(Goldstein et al., 2005)* |
|  | 15 | 0.5-4 | 2 | 2 | *(Yong et al., 2004)* |  |  | 16 | 0.25-0.5 | 0.5 | 0.5 | *(Ednie et al., 2002)* |
|  | 12 | 1-2 | 2 | 2 | *(Ednie et al., 2002)* |  |  | 12 | 0.25-0.5 | 0.5 | 0.5 | *(Goldstein et al., 2004)* |
|  | 18 | 2-16 | 2 | 16 | *(Citron et al., 2003)* |  |  | 30 | 0.25-1 | 0.5 | 0.5 | *(Edlund et al., 1999)* |
|  | 50 | 2-16 | 4 | 8 | *(Mathur et al., 2011)* |  |  | 304 | 0.25-2 | 0.5 | 1 | *(Oprica & Nord, 2005)* |
|  | 10 | 2-2 | 2 | 2 | *(Goldstein et al., 2020)* |  | *Lactobacillus spp.* | 37 | 0.5-16 | 4 | 8 | *(Citron et al., 2003)* |
|  | 14 | 2-8 | 2 | 8 | *(Goldstein et al., 2005)* |  |  | 16 | 1-8 | 4 | 8 | *(Goldstein et al., 2004)* |
|  | 14 | 2-8 | 2 | 8 | *(Goldstein et al., 2004)* |  |  | 16 | 1-8 | 4 | 8 | *(Goldstein et al., 2005)* |
| *Clostridium perfringens* | 20 | 1-2 | 2 | 2 | *(Ednie et al., 2002)* |  | *Eubacterium group* | 13 | 0.5-2 | 2 | 2 | *(Goldstein et al., 2005)* |
|  | 15 | 1-2 | 2 | 2 | *(Yum et al., 2010)* |  |  | 13 | 0.5-2 | 2 | 2 | *(Goldstein et al., 2004)* |
|  | 50 | 1-4 | 2 | 2 | *(Edlund et al., 1999)* |  |  | 31 | 0.06-8 | 1 | 8 | *(Citron et al., 2003)* |
|  | 11 | 1-4 | 2 | 2 | *(Citron et al., 2003)* |  | *Eubacterium lentum* | 10 | 0.5-2 | 2 | 2 | *(Goldstein et al., 2004)* |
|  | 10 | 1-16 | 2 | 2 | *(Goldstein et al., 2020)* |  |  | 9 | 1-2 | 2 |  | *(Goldstein et al., 2005)* |
|  | 12 | 2 | 2 | 2 | *(Goldstein et al., 2005)* |  |  | 17 | 1-2 | 1 | 2 | *(Citron et al., 2003)* |
|  | 17 | 2 | 2 | 2 | *(Yong et al., 2004)* |  | *Peptostreptococcus spp.* | 59 | 0.25-2 | 0.5 | 1 | *(Yum et al., 2010)* |
|  | 12 | 2-2 | 2 | 2 | *(Goldstein et al., 2004)* |  |  | 13 | 0.5-16 | 1 | 2 | *(Citron et al., 2003)* |
| **Organism** | **N** | **MIC range** | **MIC_50_** | **MIC_90_** | **Reference** |  | **Organism** | **N** | **MIC range** | **MIC_50_** | **MIC_90_** | **Reference** |
|  | 27 | 0.5-2 | 1 | 2 | *(Lee et al., 2015)* |  | *Porphyromonas spp.* | 6 | ≤0.06-1 |  |  | *(Behra-Miellet et al., 2003)* |
|  | 10 | 0.5-2 | 1 | 2 | *(Goldstein et al., 1999)* |  |  | 10 | 0.5-0.4 | 1 | 2 | *(Goldstein et al., 2020)* |
|  | 75 | 0.5-2.0 | 1 | 2 | *(Ednie et al., 2002)* |  |  | 25 | 0.25-2 | 1 | 2 | *(Goldstein et al., 1999)* |
|  | 27 | 0.5-4 | 1 | 4 | *(Phillips et al., 2003)* |  |  | 23 | 0.5-2 | 1 | 2 | *(Molitoris et al., 2006)* |
|  | 56 | 0.5-8 | 1 | 2 | *(Yong et al., 2004)* |  | *Fusobacterium spp.* | 21 | ≤0.06-2 | 0.5 | 1 | *(Behra-Miellet et al., 2003)* |
|  | 15 | 0.5-8 | 2 | 4 | *(Mathur et al., 2011)* |  |  | 21 | 0.064-8 | 0.25 | 1 | *(Wybo et al., 2014)* |
| *Bacteroides spp.* | 30 | 2-16 | 4 | 8 | *(Mathur et al., 2011)* |  |  | 10 | 0.25-0.5 | 0.5 | 0.5 | *(Goldstein et al., 2020)* |
| *Bacteroides fragilis* | 455 | ≤0.5-8 | 2 | 4 | *(Snydman et al., 2017)* |  |  | 24 | 0.25-2 | 0.5 | 2 | *(Ednie et al., 2002)* |
|  | 69 | 1-16 | 2 | 4 | *(Wybo et al., 2014)* |  |  | 6 | 2 | 2 | 2 | *(Phillips et al., 2003)* |
|  | 46 | 2-16 | 4 | 4 | *(Goldstein et al., 2020)* |  |  | 10 | 0.125-1 | 0.5 | 0.5 | *(Goldstein et al., 1999)* |
|  | 57 | 2-16 | 4 | 4 | *(Goldstein et al., 2017)* |  |  | 35 | 0.25-2 | 0.5 | 1 | *(Molitoris et al., 2006)* |
|  | 57 | 2-4 | 4 | 4 | *(Phillips et al., 2003)* |  | *Veillonella spp.* | 5 | ≤0.06-0.5 |  |  | *(Behra-Miellet et al., 2003)* |
|  | 52 | 2-4 | 4 | 4 | *(Behra-Miellet et al., 2003)* |  |  | 10 | 2-8 | 2 | 4 | *(Goldstein et al., 2020)* |
|  | 30 | 2-4 | 4 | 4 | *(Yum et al., 2010)* |  | ***Others***  *Listeria monocytogenes* | 6 | 0.5-2 | 1 | 2 | *(Yu et al., 2021)* |
|  | 41 | 2-8 | 4 | 4 | *(Molitoris et al., 2006)* |  |  | 60 | 0.75-1.5 |  | 1 | *(Callapina et al., 2001)* |
|  | 34 | 4 | 4 | 4 | *(Yong et al., 2004)* |  | *Listeria spp.* | 27 | 2 | 2 | 2 | *(Jones et al., 2002)* |
|  | 10 | 4-8 | 4 | 4 | *(Ednie et al., 2002)* |  |  | 24 |  | 1 | 2 | *(Mendes et al., 2015)* |
| *Prevotella spp.* | 44 | ≤0.06-8 | 2 | 4 | *(Behra-Miellet et al., 2003)* |  | *Rhodococcus equi* | 103 | 0.5-2 | 2 | 2 | *(Bowersock et al., 2000)* |
|  | 12 | 0.25-2 | 0.5 | 1 | *(Citron et al., 2003)* |  |  | 12 | 0.5-2 | 0.5 |  | *(Giacometti et al., 2005)* |
|  | 10 | 1-8 | 4 | 8 | *(Mathur et al., 2011)* |  |  | 70 | ≤1 | ≤1 | ≤1 | *(Erol et al., 2021)* |
|  | 28 | 0.25-4 | 1 | 4 | *(Molitoris et al., 2006)* |  | *Micrococcus spp.* | 11 |  | 0.5 | 0.5 | *(Mendes et al., 2015)* |
|  | 15 | 1-2 | 2 | 2 | *(Goldstein et al., 1999)* |  |  | 11 | 1 | 1 | 0.5-1 | *(Jones et al., 2002)* |

Abbreviations: N, number of strains; MIC, minimum inhibitory concentration

**REFERENCES**

**Abe M, Kimura M, Maruyama H, Watari T, Ogura S, Takagi S, Uchida N, Otsuka Y, Taniguchi S, Araoka H**. **2021**. Clinical characteristics and drug susceptibility patterns of Corynebacterium species in bacteremic patients with hematological disorders. *European journal of clinical microbiology & infectious diseases : official publication of the European Society of Clinical Microbiology* **40(10)**:2095-2104 DOI 10.1007/s10096-021-04257-8.

**Barberis CM, Sandoval E, Rodriguez CH, Ramírez MS, Famiglietti A, Almuzara M, Vay C**. **2018**. Comparison between disk diffusion and agar dilution methods to determine in vitro susceptibility of Corynebacterium spp. clinical isolates and update of their susceptibility. *Journal of global antimicrobial resistance* **14**:246-252 DOI 10.1016/j.jgar.2018.05.009.

**Behra-Miellet J, Calvet L, Dubreuil L**. **2003**. Activity of linezolid against anaerobic bacteria. *International journal of antimicrobial agents* **22(1)**:28-34 DOI 10.1016/s0924-8579(03)00087-6.

**Bowersock TL, Salmon SA, Portis ES, Prescott JF, Robison DA, Ford CW, Watts JL**. **2000**. MICs of oxazolidinones for Rhodococcus equi strains isolated from humans and animals. *Antimicrobial agents and chemotherapy* **44(5)**:1367-1369 DOI 10.1128/AAC.44.5.1367-1369.2000.

**Callapina M, Kretschmar M, Dietz A, Mosbach C, Hof H, Nichterlein T**. **2001**. Systemic and intracerebral infections of mice with Listeria monocytogenes successfully treated with linezolid. *Journal of chemotherapy* **13(3)**:265-269 DOI 10.1179/joc.2001.13.3.265.

**Chapartegui-González I, Fernández-Martínez M, Rodríguez-Fernández A, Rocha D, Aguiar E, Pacheco L, Ramos-Vivas J, Calvo J, Martínez-Martínez L, Navas J**. **2020**. Antimicrobial Susceptibility and Characterization of Resistance Mechanisms of Corynebacterium urealyticum Clinical Isolates. *Antibiotics (Basel, Switzerland)* **9(7)**:404 DOI 10.3390/antibiotics9070404.

**Citron DM, Merriam CV, Tyrrell KL, Warren YA, Fernandez H, Goldstein EJ**. **2003**. In vitro activities of ramoplanin, teicoplanin, vancomycin, linezolid, bacitracin, and four other antimicrobials against intestinal anaerobic bacteria. *Antimicrobial agents and chemotherapy* **47(7)**:2334-2338 DOI 10.1128/AAC.47.7.2334-2338.2003.

**Edlund C, Oh H, Nord CE**. **1999**. In vitro activity of linezolid and eperezolid against anaerobic bacteria. *Clinical microbiology and infection : the official publication of the European Society of Clinical Microbiology and Infectious Diseases* **5(1)**:51-53 DOI 10.1111/j.1469-0691.1999.tb00099.x.

**Ednie LM, Jacobs MR, Appelbaum PC**. **2002**. Anti-anaerobic activity of AZD2563, a new oxazolidinone, compared with eight other agents. *The Journal of antimicrobial chemotherapy* **50(1)**:101-105 DOI 10.1093/jac/dkf088.

**Erol E, Scortti M, Fortner J, Patel M, Vázquez-Boland JA**. **2021**. Antimicrobial Resistance Spectrum Conferred by pRErm46 of Emerging Macrolide (Multidrug)-Resistant Rhodococcus equi. *Journal of clinical microbiology* **59(10)**:e0114921 DOI 10.1128/JCM.01149-21.

**Fernandez-Roblas R, Adames H, Martín-de-Hijas NZ, Almeida DG, Gadea I, Esteban J**. **2009**. In vitro activity of tigecycline and 10 other antimicrobials against clinical isolates of the genus Corynebacterium. *International journal of antimicrobial agents* **33(5)**:453-455 DOI 10.1016/j.ijantimicag.2008.11.001.

**Giacometti A, Cirioni O, Kamysz W, Silvestri C, Del Prete MS, Licci A, D'Amato G, Lukasiak J, Scalise G**. **2005**. In vitro activity of citropin 1.1 alone and in combination with clinically used antimicrobial agents against Rhodococcus equi. *The Journal of antimicrobial chemotherapy* **56(2)**:410-412 DOI 10.1093/jac/dki236.

**Goldstein E, Merriam CV, Citron DM**. **2020**. In Vitro Activity of Tedizolid Compared to Linezolid and Five Other Antimicrobial Agents against 332 Anaerobic Isolates, Including Bacteroides fragilis Group, Prevotella, Porphyromonas, and Veillonella Species. *Antimicrobial agents and chemotherapy* **64(9)**:e01088-01020 DOI 10.1128/AAC.01088-20.

**Goldstein EJ, Citron DM, Merriam CV**. **1999**. Linezolid activity compared to those of selected macrolides and other agents against aerobic and anaerobic pathogens isolated from soft tissue bite infections in humans. *Antimicrobial agents and chemotherapy* **43(6)**:1469-1474 DOI 10.1128/AAC.43.6.1469.

**Goldstein EJ, Citron DM, Merriam CV, Warren YA, Tyrrell KL, Fernandez HT**. **2004**. In vitro activities of the new semisynthetic glycopeptide telavancin (TD-6424), vancomycin, daptomycin, linezolid, and four comparator agents against anaerobic gram-positive species and Corynebacterium spp. *Antimicrobial agents and chemotherapy* **48(6)**:2149-2152 DOI 10.1128/AAC.48.6.2149-2152.2004.

**Goldstein EJ, Citron DM, Merriam CV, Warren YA, Tyrrell KL, Fernandez HT, Bryskier A**. **2005**. Comparative in vitro activities of XRP 2868, pristinamycin, quinupristin-dalfopristin, vancomycin, daptomycin, linezolid, clarithromycin, telithromycin, clindamycin, and ampicillin against anaerobic gram-positive species, actinomycetes, and lactobacilli. *Antimicrobial agents and chemotherapy* **49(1)**:408-413 DOI 10.1128/AAC.49.1.408-413.2005.

**Goldstein EJ, Citron DM, Tyrrell KL, Leoncio ES, Merriam CV**. **2017**. The underappreciated in vitro activity of tedizolid against Bacteroides fragilis species, including strains resistant to metronidazole and carbapenems. *Anaerobe* **43**:1-3 DOI 10.1016/j.anaerobe.2016.09.008.

**Goldstein EJ, Citron DM, Warren YA, Tyrrell KL, Merriam CV, Fernandez HT**. **2006**. In vitro activities of dalbavancin and 12 other agents against 329 aerobic and anaerobic gram-positive isolates recovered from diabetic foot infections. *Antimicrobial agents and chemotherapy* **50(8)**:2875-2879 DOI 10.1128/AAC.00286-06.

**Gómez-Garcés JL, Alos JI, Tamayo J**. **2007**. In vitro activity of linezolid and 12 other antimicrobials against coryneform bacteria. *International journal of antimicrobial agents* **29(6)**:688-692 DOI 10.1016/j.ijantimicag.2006.11.032.

**Johnson AP, Warner M, Malnick H, Livermore DM**. **2003**. Activity of the oxazolidinones AZD2563 and linezolid against Corynebacterium jeikeium and other Corynebacterium spp. *The Journal of antimicrobial chemotherapy* **51(3)**:745-747 DOI 10.1093/jac/dkg129.

**Jones RN, Biedenbach DJ, Anderegg TR**. **2002**. In vitro evaluation of AZD2563, a new oxazolidinone, tested against unusual gram-positive species. *Diagnostic microbiology and infectious disease* **42(2)**:119-122 DOI 10.1016/s0732-8893(01)00329-7.

**Lee Y, Hong SK, Choi S, Im W, Yong D, Lee K**. **2015**. In vitro activity of tedizolid against gram-positive bacteria in patients with skin and skin structure infections and hospital-acquired pneumonia: a Korean multicenter study. *Annals of laboratory medicine* **35(5)**:523-530 DOI 10.3343/alm.2015.35.5.523.

**Mathur T, Kumar M, Barman TK, Kumar GR, Kalia V, Singhal S, Raj VS, Upadhyay DJ, Das B, Bhatnagar PK**. **2011**. Activity of RBx 11760, a novel biaryl oxazolidinone, against Clostridium difficile. *The Journal of antimicrobial chemotherapy* **66(5)**:1087-1095 DOI 10.1093/jac/dkr033.

**Mendes RE, Sader HS, Flamm RK, Farrell DJ, Jones RN**. **2015**. Telavancin activity when tested by a revised susceptibility testing method against uncommonly isolated Gram-positive pathogens responsible for documented infections in hospitals worldwide (2011-2013). *Journal of global antimicrobial resistance* **3(1)**:36-39 DOI 10.1016/j.jgar.2014.12.003.

**Molitoris D, Väisänen ML, Bolaños M, Finegold SM**. **2006**. In vitro activities of DX-619 and four comparator agents against 376 anaerobic bacterial isolates. *Antimicrobial agents and chemotherapy* **50(5)**:1887-1889 DOI 10.1128/AAC.50.5.1887-1889.2006.

**Neemuchwala A, Soares D, Ravirajan V, Marchand-Austin A, Kus JV, Patel SN**. **2018**. In Vitro Antibiotic Susceptibility Pattern of Non-diphtheriae Corynebacterium Isolates in Ontario, Canada, from 2011 to 2016. *Antimicrobial agents and chemotherapy* **62(4)**:e01776-01717 DOI 10.1128/AAC.01776-17.

**Nhan TX, Parienti JJ, Badiou G, Leclercq R, Cattoir V**. **2012**. Microbiological investigation and clinical significance of Corynebacterium spp. in respiratory specimens. *Diagnostic microbiology and infectious disease* **74(3)**:236-241 DOI 10.1016/j.diagmicrobio.2012.07.001.

**Oprica C, Nord CE**. **2005**. European surveillance study on the antibiotic susceptibility of Propionibacterium acnes. *Clinical microbiology and infection : the official publication of the European Society of Clinical Microbiology and Infectious Diseases* **11(3)**:204-213 DOI 10.1111/j.1469-0691.2004.01055.x.

**Phillips OA, Rotimi VO, Jamal WY, Shahin M, Verghese TL**. **2003**. Comparative in vitro activity of PH-027 versus linezolid and other anti-anaerobic antimicrobials against clinical isolates of Clostridium difficile and other anaerobic bacteria. *Journal of chemotherapy* **15(2)**:113-117 DOI 10.1179/joc.2003.15.2.113.

**Ract P, Piau-Couapel C, Compain F, Auzou M, Michon J, Cattoir V**. **2017**. In vitro activity of tedizolid and comparator agents against Gram-positive pathogens responsible for bone and joint infections. *Journal of medical microbiology* **66(10)**:1374-1378 DOI 10.1099/jmm.0.000595.

**Rashid MU, Dalhoff A, Weintraub A, Nord CE**. **2014**. In vitro activity of MCB3681 against Clostridium difficile strains. *Anaerobe* **28**:216-219 DOI 10.1016/j.anaerobe.2014.07.001.

**Snydman DR, Jacobus NV, McDermott LA, Goldstein EJ, Harrell L, Jenkins SG, Newton D, Patel R, Hecht DW**. **2017**. Trends in antimicrobial resistance among Bacteroides species and Parabacteroides species in the United States from 2010-2012 with comparison to 2008-2009. *Anaerobe* **43**:21-26 DOI 10.1016/j.anaerobe.2016.11.003.

**Sun W, Ma L, Li Y, Xu Y, Wei J, Sa L, Chen X, Su J**. **2022**. In vitro Studies of Non-Diphtheriae Corynebacterium Isolates on Antimicrobial Susceptibilities, Drug Resistance Mechanisms, and Biofilm Formation Capabilities. *Infection and drug resistance* **15**:4347-4359 DOI 10.2147/IDR.S376328.

**Sánchez Hernández J, Mora Peris B, Yagüe Guirao G, Gutiérrez Zufiaurre N, Muñoz Bellido JL, Segovia Hernández M, García Rodríguez JA**. **2003**. In vitro activity of newer antibiotics against Corynebacterium jeikeium, Corynebacterium amycolatum and Corynebacterium urealyticum. *International journal of antimicrobial agents* **22(5)**:492-496 DOI 10.1016/s0924-8579(03)00121-3.

**Wang Y, Shi X, Zhang J, Wang Y, Lv Y, Du X, ChaoLuMen Q, Wang J**. **2021**. Wide spread and diversity of mutation in the gyrA gene of quinolone-resistant Corynebacterium striatum strains isolated from three tertiary hospitals in China. *Annals of clinical microbiology and antimicrobials* **20(1)**:71 DOI 10.1186/s12941-021-00477-0.

**Wybo I, Van den Bossche D, Soetens O, Vekens E, Vandoorslaer K, Claeys G, Glupczynski Y, Ieven M, Melin P, Nonhoff C, Rodriguez-Villalobos H, Verhaegen J, Piérard D**. **2014**. Fourth Belgian multicentre survey of antibiotic susceptibility of anaerobic bacteria. *The Journal of antimicrobial chemotherapy* **69(1)**:155-161 DOI 10.1093/jac/dkt344.

**Yong D, Yum JH, Lee K, Chong Y, Choi SH, Rhee JK**. **2004**. In vitro activities of DA-7867, a novel oxazolidinone, against recent clinical isolates of aerobic and anaerobic bacteria. *Antimicrobial agents and chemotherapy* **48(1)**:352-357 DOI 10.1128/AAC.48.1.352-357.2004.

**Yu W, Huang Y, Ying C, Zhou Y, Zhang L, Zhang J, Chen Y, Qiu Y**. **2021**. Analysis of Genetic Diversity and Antibiotic Options for Clinical Listeria monocytogenes Infections in China. *Open forum infectious diseases* **8(6)**:ofab177 DOI 10.1093/ofid/ofab177.

**Yum JH, Choi SH, Yong D, Chong Y, Im WB, Rhee DK, Lee K**. **2010**. Comparative in vitro activities of torezolid (DA-7157) against clinical isolates of aerobic and anaerobic bacteria in South Korea. *Antimicrobial agents and chemotherapy* **54(12)**:5381-5386 DOI 10.1128/AAC.00728-10.
