# Supplementary material for: The in vitro antimicrobial activity of linezolid against unconventional pathogens
Source: PeerJ. 2025 Feb 12;13:e18825. doi: 10.7717/peerj.18825 (PMC11829633; doi:10.7717/peerj.18825)
Supplement: Supplemental Information 3 [file peerj-13-18825-s003.docx]

**Table 3 In vitro activity of linezolid against M. tuberculosis, NTM and Nocardia (MIC, μg/mL).**

| **Organism** | **N** | **Method** | **MIC range** | **MIC_50_** | **MIC_90_** | **Country** | **Time of study** | **Reference** |
| --- | --- | --- | --- | --- | --- | --- | --- | --- |
| ***M. tuberculosis*** | 84 |  | 0.125-4 | 0.5 | 2 | China |  | *(Huang et al., 2008)* |
| Beijing genotype | 102 |  |  | 0.5 | 2 | China |  | *(Zhang et al., 2014)* |
| Non-Beijing genotype | 56 |  |  | 0.25 | 0.5 | China |  | *(Zhang et al., 2014)* |
| MDR | 45 |  | 0.125-0.5 | 0.25 | 0.25 | China | 2007-2009 | *(Yang et al., 2012)* |
| XDR | 16 |  | 0.125-0.5 | 0.25 | 0.25 | China | 2007-2009 | *(Yang et al., 2012)* |
| MDR | 15 |  | 0.063-16 | 0.25 | 4 | China | 2012-2013 | *(Zhang et al., 2014)* |
| XDR | 90 | MABA | 0.125-32 | 0.5 | 0.5 | China | 2016 | *(Pang et al., 2017)* |
| MDR | 120 | MABA |  | 0.064 | 1 | China | 2017-2017 | *(Zong et al., 2018)* |
| XDR | 120 | MABA |  | 0.13 | 0.25 | China | 2017-2017 | *(Zong et al., 2018)* |
|  | 88 |  | 0.03-2 | 0.12 | 0.5 | China | 2014-2016 | *(Zheng et al., 2021)* |
| MDR | 425 | BMD | 0.12-8 |  |  | China | 2018-2019 | *(Yao et al., 2021)* |
| All | 1452 | BMD | 0.06-32 |  |  | China | 2020-2021 | *(Guo et al., 2023)* |
| MDR | 156 | BMD |  | 0.25 | 1 | China | 2020-2021 | *(Guo et al., 2023)* |
| Non-MDR | 1296 | BMD |  |  |  | China | 2020-2021 | *(Guo et al., 2023)* |
| Pre XDR | 93 | BMD |  | 0.5 | 1 | China | 2020-2021 | *(Guo et al., 2023)* |
| XDR | 27 | BMD |  | 1 | 32 | China | 2020-2021 | *(Guo et al., 2023)* |
| Drug-resistant | 39 |  | 0.125-0.5 | 0.125 | 0.25 | China |  | *(An et al., 2023)* |
| Drug-sensitive | 9 |  | 0.25-0.5 | 0.25 | 0.5 | China |  | *(An et al., 2023)* |
| Drug-susceptible | 10 | MABA | 0.095-0.3 |  |  | China |  | *(Guo et al., 2021)* |
| MDR | 30 | MABA | 0.036-0.499 |  |  | China |  | *(Guo et al., 2021)* |
|  | 69 | MABA |  | 0.5 | 1 | China |  | *(Wang et al., 2022)* |
|  | 169 | ADM | 0.125-> 2 | 0.5 | 1 | India | 2019 | *(Singh et al., 2022)* |
| MDR | 39 | ADM | 0.25 to >16 | 0.25 | 0.5 | Iran | 2013-2014 | *(Kazemian et al., 2015)* |
| MDR | 35 | BMD |  | 0.25 | 0.5 | Iran | 2014-2018 | *(Kardan-Yamchi et al., 2020)* |
| MDR | 54 | BMD | 0.125-2 | 0.5 | 1 | Japan |  | *(Aono et al., 2022)* |
|  | 420 | BMD | ≤0.125 - 1 | 0.5 | 0.5 | Korea |  | *(Yang et al., 2018)* |
| XDR | 59 | APM | 0.25-2 | 0.5 | 0.5 | Pakistan | 2010-2011 | *(Ahmed et al., 2013)* |
| Pre-XDR | 43 | APM | 0.25-1 | 0.5 | 0.5 | Pakistan | 2010-2011 | *(Ahmed et al., 2013)* |
|  | 117 | APM | ≤0.125-1 | 0.5 | 1 | Spain | 1988-2000 | *(Alcalá et al., 2003)* |
|  | 42 | APM | 0.12-0.5 | 0.25 | 0.5 | Spain | 1997-2004 | *(Tato et al., 2006)* |
| Resistant to INH | 3 | APM | 0.12-0.5 |  |  | Spain | 1997-2004 | *(Tato et al., 2006)* |
| Resistant to INH, RIF | 10 | APM | 0.12-0.5 | 0.25 | 0.5 | Spain | 1997-2004 | *(Tato et al., 2006)* |
|  | 42 |  | 0.12-0.5 | 0.5 | 0.5 | Spain | 1997-2004 | *(Tato et al., 2006)* |
| Resistant to INH | 3 |  | 0.12-0.5 |  |  | Spain | 1997-2004 | *(Tato et al., 2006)* |
| Resistant to INH, RIF | 10 |  | 0.12-0.5 | 0.5 | 0.5 | Spain | 1997-2004 | *(Tato et al., 2006)* |
| MDR | 39 | RPM |  | 4 | 8 | Turkey |  | *(Erturan & Uzun, 2005)* |
| non-MDR | 34 | APM | 0.06-1 | 0.5 | 0.5 | Turkey |  | *(Ermertcan et al., 2009)* |
| MDR | 33 | APM | 0.06-1 | 0.5 | 0.5 | Turkey |  | *(Ermertcan et al., 2009)* |
| All | 59 | ADM | 0.125-64 | 1 | 32 |  |  | *(Sood et al., 2005)* |
| MDR (Resistant to both INH and RIF) | 16 | ADM | 0.125-64 | 4 | 64 |  |  | *(Sood et al., 2005)* |
| Resistant to either INH or RIF | 33 | ADM | 0.125-8 | 1 | 1 |  |  | *(Sood et al., 2005)* |
|  | 22 | BMD |  | 0.5 | 1 |  |  | *(Shoen et al., 2018)* |
| MDR | 153 | BMD | ＜0.06-0.5 | 0.25 | 0.25 | USA |  | *(Cavanaugh et al., 2017)* |
|  | 67 |  | 0.25-4 | 1 | 2 | Mexico |  | *(Vera-Cabrera et al., 2005)* |
| **RGM**  *M. abscessus* | 21 | BMD | 16-64 | 64 | 64 | China |  | *(Zhao et al., 2015)* |
|  | 47 | BMD |  | 8 | ＞32 | Netherlands |  | *(Araj et al., 2019)* |
|  | 53 | BMD |  |  |  | China | 2005-2012 | *(Li et al., 2017)* |
| *M. abscessus subsp. abscessus* | 148 | BMD | 1-64 | 8 | 32 | China |  | *(Guo et al., 2021)* |
|  | 45 | BMD | 1-32 | 4 | 8 | China |  | *(Nie et al., 2014)* |
|  | 20 | BMD | 0.5-32 | 2 | 16 | China | 2011-2012 | *(Zhang et al., 2017)* |
|  | 67 | BMD |  |  |  | China | 2013 | *(Liu et al., 2021)* |
|  | 44 | BMD | 1-16 | 8 | 16 | China |  | *(Gao et al., 2023)* |
|  | 28 | BMD | 2-＞32 | ＞32 |  | China | 2012-2016 | *(Lee et al., 2017)* |
|  | 30 | BMD | 2-128 | 16 | 64 | Iran | 2011-2014 | *(Heidarieh et al., 2016)* |
|  | 47 | BMD |  | 8 | ＞32 | Netherlands |  | *(Ruth et al., 2020)* |
|  | 47 | BMD | 0.5-64 | 4 | 8 | Korea |  | *(Kim et al., 2021)* |
|  | 12 | BMD | 0.25-32 | 1 | 4 | Korea |  | *(Kim et al., 2021)* |
|  | 81 | BMD | 0.12-128 | 16 | 32 | USA | 2014-2015 | *(Brown-Elliott & Wallace Jr, 2017)* |
|  | 4 | BMD | 4-16 | 4 | 16 | Turkey | 2010-2013 | *(Senol et al., 2022)* |
|  | 14 | BMD | 8-64 | 64 | 64 | USA |  | *(Vera-Cabrera et al., 2006)* |
|  | 33 | BMD | 2-32 | 8 | 16 | USA | 2016-2018 | *(Brown-Elliott et al., 2018)* |
|  | 1344 | BMD |  | ＞16 | ＞16 | USA | 2018-2020 | *(Hunkins et al., 2023)* |
|  | 43 | BMD | 0.0625-＞32 | 8 | ＞32 |  |  | *(Tang et al., 2018)* |
| *M. abscessus subsp. massiliense/M. abscessus subsp. abscessus hybrid* | 6 | BMD | 2-＞128 | 16 |  | USA | 2014-2015 | *(Brown-Elliott & Wallace Jr, 2017)* |
| *M. abscessus/massiliense hybrid* | 10 | BMD | 2-16 | 8 | 16 | USA | 2016-2018 | *(Brown-Elliott et al., 2018)* |
| *M. abscessus complex* | 20 | BMD | 2-128 |  | 16 | China | 2009-2013 | *(Shen et al., 2018)* |
|  | 53 | BMD |  |  |  | China | 2016-2019 | *(Zhang et al., 2020)* |
|  | 35 | BMD | 0.5-32 | 16 | 16 | China | 2016-2021 | *(Zhang et al., 2022)* |
|  | 114 | BMD |  |  |  | China | 2013 | *(Liu et al., 2021)* |
|  | 24 | BMD |  | 16 | 32 | China | 2019-2021 | *(He et al., 2022)* |
|  | 37 | BMD | 1-64 | 16 | 32 | Spain |  | *(Marfil et al., 2022)* |
|  | 65 | BMD | 1-32 | 4 | 16 | China |  | *(Gao et al., 2023)* |
| *M. abscessus subsp. bolletii* | 93 | BMD |  | 16 | ＞16 | USA | 2018-2020 | *(Hunkins et al., 2023)* |
|  | 25 | BMD | 4-＞128 | 8 | 16 | China |  | *(Nie et al., 2014)* |
|  | 1 | BMD | 32 |  |  | China | 2012-2018 | *(Lee et al., 2017)* |
|  | 5 | BMD | 8-＞32 | 32 | ＞32 |  |  | *(Tang et al., 2018)* |
| *M. abscessus subsp. massiliense* | 45 | BMD |  |  |  | China | 2013 | *(Liu et al., 2021)* |
|  | 9 | BMD |  |  |  | China | 2005-2012 | *(Li et al., 2017)* |
|  | 20 | BMD | 0.5-8 | 2 | 4 | China | 2011-2013 | *(Zhang et al., 2017)* |
|  | 21 | BMD | 1-32 | 4 | 8 | China |  | *(Gao et al., 2023)* |
|  | 38 | BMD | 2-＞32 | 32 | ＞32 | China | 2012-2017 | *(Lee et al., 2017)* |
|  | 46 | BMD | 0.5-64 | 8 | 32 | China |  | *(Guo et al., 2021)* |
|  | 50 | BMD | 0.5-16 | 4 | 8 | Korea |  | *(Kim et al., 2021)* |
|  | 18 | BMD | 1-16 | 2 | 8 | Korea |  | *(Kim et al., 2021)* |
|  | 12 | BMD | 0.5-32 | 8 | 32 | USA | 2014-2015 | *(Brown-Elliott & Wallace Jr, 2017)* |
|  | 10 | BMD | 2-16 | 8 | 16 | USA | 2016-2018 | *(Brown-Elliott et al., 2018)* |
|  | 754 | BMD |  | ＞16 | ＞16 | USA | 2018-2020 | *(Hunkins et al., 2023)* |
|  | 82 | BMD | 0.5-＞32 | 8 | ＞32 |  |  | *(Tang et al., 2018)* |
| *M. chelonae complex* | 17 | BMD | 1-32 | 8 | 16 | USA |  | *(Vera-Cabrera et al., 2006)* |
| *M. chelonae* | 2 | BMD | 8-16 |  |  | Netherlands |  | *(Ruth et al., 2020)* |
|  | 22 | BMD | 2-16 | 8 | 16 | USA | 2014-2015 | *(Brown-Elliott & Wallace Jr, 2017)* |
|  | 11 | BMD | 2-16 | 4 | 8 | China |  | *(Zhao et al., 2015)* |
|  | 1 | BMD | 4 |  |  | China | 2016-2021 | *(Zhang et al., 2022)* |
|  | 526 | BMD |  | 16 | ＞16 | USA | 2018-2020 | *(Hunkins et al., 2023)* |
|  | 10 | BMD | 2-16 | 8 | 16 | USA | 2016-2018 | *(Brown-Elliott et al., 2018)* |
|  | 39 | BMD | 2-128 | 16 | 64 | Iran | 2012-2014 | *(Heidarieh et al., 2016)* |
|  | 1 | BMD | 4 |  |  | China | 2016-2021 | *(Zhang et al., 2022)* |
|  | 2 | BMD | 8-16 |  |  |  |  | *(Araj et al., 2019)* |
| *M. fortuitum* | 21 | BMD | 1-32 | 16 | 32 | China | 2016-2021 | *(Zhang et al., 2022)* |
|  | 24 | BMD | 0.5-8 | 4 | 8 | China |  | *(Zhao et al., 2015)* |
|  | 9 | BMD |  |  |  | China | 2005-2012 | *(Li et al., 2017)* |
|  | 53 | BMD | 0.0625-64 | 64 | 64 | China | 2012-2014 | *(Zheng et al., 2017)* |
|  | 17 | BMD |  |  |  | China | 2016-2020 | *(Zhang et al., 2020)* |
|  | 85 | BMD | 0.25-64 | 2 | 32 | Iran | 2010-2014 | *(Heidarieh et al., 2016)* |
|  | 2 | BMD | 2 |  |  | Netherlands |  | *(Ruth et al., 2020)* |
|  | 17 | BMD | 8-32 |  | 16 | China | 2009-2014 | *(Shen et al., 2018)* |
|  | 21 | BMD | 1-32 | 16 | 32 | China | 2016-2021 | *(Zhang et al., 2022)* |
|  | 20 | BMD | 1-8 | 2 | 4 | USA | 2014-2015 | *(Brown-Elliott & Wallace Jr, 2017)* |
|  | 1 | BMD | 32 |  |  | Turkey | 2010-2013 | *(Senol et al., 2022)* |
|  | 564 | BMD |  | 16 | ＞16 | USA | 2018-2020 | *(Hunkins et al., 2023)* |
|  | 2 | BMD | 2 |  |  |  |  | *(Araj et al., 2019)* |
| *M. fortuitum 3rd biovariant complex* | 24 | BMD | 1-8 | 8 | 8 | USA |  | *(Vera-Cabrera et al., 2006)* |
| *M. fortuitum group* | 10 | BMD | ≤1-8 | 2 | 4 | USA | 2016-2018 | *(Brown-Elliott et al., 2018)* |
|  | 33 | BMD | 0.5-≥64 | 4 | 16 | USA |  | *(Vera-Cabrera et al., 2006)* |
| *M. mucogenicum* | 4 | BMD | <0.5-2 |  |  | China | 2016-2021 | *(Zhang et al., 2022)* |
|  | 4 | BMD | <0.5-2 |  |  | China | 2016-2021 | *(Zhang et al., 2022)* |
| *M. mucogenicum group* | 10 | BMD | ≤1-4 | ≤1 | 4 | USA | 2016-2018 | *(Brown-Elliott et al., 2018)* |
|  | 9 | BMD | 0.5-8 | 1 |  | USA | 2014-2015 | *(Brown-Elliott & Wallace Jr, 2017)* |
| *M. mucogenicum* | 163 | BMD |  | 2 | 8 | USA | 2018-2020 | *(Hunkins et al., 2023)* |
| *M. ltetiense* | 1 | BMD | 16 |  |  | China | 2016-2021 | *(Zhang et al., 2022)* |
|  | 1 | BMD | 16 |  |  | China | 2016-2021 | *(Zhang et al., 2022)* |
| *M. immunogenum* | 29 | BMD |  | 16 | ＞16 | USA | 2018-2020 | *(Hunkins et al., 2023)* |
|  | 10 | BMD | 4-32 | 16 | 32 | USA | 2016-2018 | *(Brown-Elliott et al., 2018)* |
|  | 9 | BMD | 0.12-16 | 8 |  | USA | 2014-2015 | *(Brown-Elliott & Wallace Jr, 2017)* |
| *M. goodii* | 21 | BMD |  | 2 | 8 | USA | 2018-2020 | *(Hunkins et al., 2023)* |
| *M. mageritense* | 30 | BMD |  | 4 | 16 | USA | 2018-2020 | *(Hunkins et al., 2023)* |
| *M. neoaurum* | 29 | BMD |  | ≤1 | ≤1 | USA | 2018-2020 | *(Hunkins et al., 2023)* |
| *M. peregrinum* | 46 | BMD |  | 4 | 16 | USA | 2018-2020 | *(Hunkins et al., 2023)* |
| *M. porcinum* | 93 | BMD |  | 8 | 16 | USA | 2018-2020 | *(Hunkins et al., 2023)* |
| *M. senegalense* | 69 | BMD |  | 8 | ＞16 | USA | 2018-2020 | *(Hunkins et al., 2023)* |
| *M. septicum* | 12 |  |  |  |  | USA | 2014-2020 | *(Go et al., 2020)* |
| *M. smegmatis* | 1 | BMD | ＜0.5 |  |  | Turkey | 2010- 2013 | *(Senol et al., 2022)* |
| *M. szulgai* | 10 | BMD | ≤2-4 | ≤2 | 4 | USA |  | *(Brown-Elliott et al., 2003)* |
| **SGM** *M. avium* | 20 | E-test |  |  |  | Greece | 2000-2009 | *(Gitti et al., 2011)* |
|  | 31 | BMD | 8-64 | 32 | 32 | China |  | *(Zhao et al., 2015)* |
|  | 65 | BMD | 0.0625-64 | 0.5 | 4 | China | 2011-2012 | *(Zhang et al., 2015)* |
|  | 97 | BMD |  |  |  | China | 2005-2012 | *(Li et al., 2017)* |
|  | 41 | BMD |  |  |  | China | 2016-2020 | *(Yu et al., 2021)* |
|  | 8 | BMD |  |  |  | China | 2016-2022 | *(Zhang et al., 2020)* |
|  | 8 | BMD |  |  |  | Korea | 2000-2021 | *(Lee et al., 2022)* |
|  | 51 | BMD |  | 16 | 32 | Netherlands |  | *(Ruth et al., 2020)* |
|  | 161 |  |  | 32 | 64 | Russian | 2010-2016 | *(Litvinov et al., 2018)* |
|  | 885 | BMD |  | 16 | 64 | Korea | 2011-2016 | *(Cho et al., 2018)* |
|  | 52 | BMD | 0.5-＞64 | 64 | 64 | Korea |  | *(Kim et al., 2021)* |
|  | 10 | BMD | 2-64 | 32 | 32 | Korea |  | *(Kim et al., 2021)* |
|  | 8 | BMD | 16-64 | 32 | 64 | China | 2011-2014 | *(Huang et al., 2018)* |
|  | 6 | BMD | 2-32 | 16 | 32 | Turkey | 2010-2013 | *(Senol et al., 2022)* |
|  | 12 | BMD | 2-64 | 32 | 64 | USA | 2016-2018 | *(Brown-Elliott et al., 2018)* |
|  |  | BMD |  | 16 | 32 |  |  | *(Araj et al., 2019)* |
| *M. avium complex* | 13 | BMD | 2-32 | 32 | 32 | USA |  | *(Vera-Cabrera et al., 2006)* |
|  | 40 | BMD | 4-＞128 | 64 | 128 | China | 2016-2021 | *(Zhang et al., 2022)* |
|  | 43 | BMD |  | 32 | ＞64 | China | 2019-2022 | *(He et al., 2022)* |
|  | 10 | BMD | 8-64 | 32 | 64 | USA | 2016-2018 | *(Brown-Elliott et al., 2018)* |
|  | 189 | BMD | ≤2-＞32 | 32 | 64 | USA |  | *(Brown-Elliott et al., 2003)* |
|  | 100 | BMD | 2-128 | 32 | 64 | USA | 2014-2015 | *(Brown-Elliott & Wallace Jr, 2017)* |
| *M. avium‒intracellulare complex* | 108 | BMD | 1-64 | 32 | 64 | China | 2013 | *(Liu et al., 2021)* |
|  | 31 | ADM | 0.125-64 | 16 | 64 | USA and India |  | *(Sood et al., 2005)* |
| *M. intracellulare* | 48 | BMD |  |  |  | China | 2016-2019 | *(Yu et al., 2021)* |
|  | 17 | BMD | 8-64 | 8 | 16 | China |  | *(Zhao et al., 2015)* |
|  | 188 | BMD | 0.5-32 | 8 | 16 | China | 2011-2013 | *(Zhang et al., 2015)* |
|  | 172 | BMD |  |  |  | China | 2005-2012 | *(Li et al., 2017)* |
|  | 16 |  |  | 16 | 32 | Russian | 2010-2016 | *(Litvinov et al., 2018)* |
|  | 685 | BMD |  | 32 | 64 | Korea | 2011-2017 | *(Cho et al., 2018)* |
|  | 16 | BMD | ≤0.0625-64 | 32 | 64 | Korea |  | *(Kim et al., 2021)* |
|  | 6 | BMD |  |  |  | Korea | 2000-2022 | *(Lee et al., 2022)* |
|  | 5 | E-test |  |  |  | Greece | 2000-2010 | *(Gitti et al., 2011)* |
|  |  | BMD | 16-32 |  |  |  |  | *(Araj et al., 2019)* |
|  | 19 | BMD | 8-＞64 | 32 | 64 | USA | 2016-2018 | *(Brown-Elliott et al., 2018)* |
|  | 2 | BMD | 16-32 |  |  | Netherlands |  | *(Ruth et al., 2020)* |
|  | 27 | BMD |  |  |  | China | 2016-2021 | *(Zhang et al., 2020)* |
|  | 75 | BMD | 4-64 | 32 | ＞64 | China | 2011-2013 | *(Huang et al., 2018)* |
|  | 45 | BMD | 2-64 | 32 | 64 | Korea |  | *(Kim et al., 2021)* |
|  | 13 | BMD | 1-32 | 4 | 16 | Turkey | 2010-2013 | *(Senol et al., 2022)* |
| *M. gordonae* | 24 | BMD |  |  |  | China | 2005-2012 | *(Li et al., 2017)* |
|  | 2 | BMD | 4-16 |  |  | China | 2016-2021 | *(Zhang et al., 2022)* |
|  | 1 | BMD | 0.5 |  |  | Turkey | 2010- 2013 | *(Senol et al., 2022)* |
|  | 2 | E-test |  |  |  | Greece | 2000-2014 | *(Gitti et al., 2011)* |
|  | 21 | BMD | ≤0.5-16 | ≤2 | 4 | USA |  | *(Brown-Elliott et al., 2003)* |
|  | 2 | BMD | 4-16 |  |  | China | 2016-2021 | *(Zhang et al., 2022)* |
| *M. kansasii* | 26 | BMD | 0.5-1 | 1 | 1 | China |  | *(Zhao et al., 2015)* |
|  | 3 | BMD |  |  |  | China | 2016-2023 | *(Zhang et al., 2020)* |
|  | 31 | BMD | 1-64 | 2 | 4 | China | 2013 | *(Liu et al., 2021)* |
|  | 18 | BMD | 2-64 | 8 | 32 | China | 2016-2021 | *(Zhang et al., 2022)* |
|  | 112 |  |  | 4 | 32 | Russian | 2010-2016 | *(Litvinov et al., 2018)* |
|  | 45 | BMD | 0.5-＞64 | 2 | 2 | Korea |  | *(Kim et al., 2021)* |
|  | 1 | BMD |  |  |  | Korea | 2000-2023 | *(Lee et al., 2022)* |
|  | 10 | E-test |  |  |  | Greece | 2000-2011 | *(Gitti et al., 2011)* |
|  | 8 | BMD | ≤1-4 | ≤1 | 4 | USA | 2016- 2018 | *(Brown-Elliott et al., 2018)* |
|  | 19 | BMD | ≤0.5-≤2 | ≤2 | ≤2 | USA |  | *(Brown-Elliott et al., 2003)* |
|  | 7 | BMD | 0.5-2 | 2 |  | USA | 2014-2015 | *(Brown-Elliott & Wallace Jr, 2017)* |
|  | 42 | BMD |  |  |  | China | 2016-2021 | *(Yu et al., 2021)* |
|  | 18 | BMD | 2-64 | 8 | 32 | China | 2016-2021 | *(Zhang et al., 2022)* |
|  | 14 | BMD |  | 2 | 2 | China | 2019-2023 | *(He et al., 2022)* |
|  | 6 | BMD | 0.5-1 | 0.5 | 1 | Turkey | 2010- 2013 | *(Senol et al., 2022)* |
|  | 40 | BMD | 0.125-2 | 0.125 | 1 | Iran | 2013-2014 | *(Heidarieh et al., 2016)* |
| *M. xenopi* | 74 |  |  | 4 | 16 | Russian | 2010-2016 | *(Litvinov et al., 2018)* |
|  | 1 | E-test |  |  |  | Greece | 2000-2015 | *(Gitti et al., 2011)* |
| *M. marinum* | 10 | BMD | <0.5-16 |  |  | China | 2016-2021 | *(Zhang et al., 2022)* |
|  | 2 | E-test |  |  |  | Greece | 2000-2013 | *(Gitti et al., 2011)* |
|  | 9 | BMD | ≤1-2 | 2 | 2 | USA | 2016- 2018 | *(Brown-Elliott et al., 2018)* |
|  | 47 | BMD | 1-2 | ≤2 | 2 | USA |  | *(Brown-Elliott et al., 2003)* |
|  | 7 | BMD | 1-4 | 1 |  | USA | 2014-2015 | *(Brown-Elliott & Wallace Jr, 2017)* |
|  | 10 | BMD | <0.125-2 |  |  | China | 2016-2021 | *(Zhang et al., 2022)* |
| *M. chimaera* |  | BMD | 8-16 |  |  |  |  | *(Araj et al., 2019)* |
|  | 2 | BMD | 8-16 |  |  | Netherlands |  | *(Ruth et al., 2020)* |
| *M. malmoense* | 1 | BMD | 0.5 |  |  | Turkey | 2010- 2013 | *(Senol et al., 2022)* |
| *M. scrofulaceum* | 2 | E-test |  |  |  | Greece | 2000-2012 | *(Gitti et al., 2011)* |
| *M. simiae* | 10 | BMD | 16-＞64 | 32 | 64 | USA | 2016- 2018 | *(Brown-Elliott et al., 2018)* |
|  | 15 | BMD | 8-＞32 | 32 | ＞32 | USA |  | *(Brown-Elliott et al., 2003)* |
|  | 8 | BMD | 2-128 | 64 |  | USA | 2014-2015 | *(Brown-Elliott & Wallace Jr, 2017)* |
|  | 53 | BMD | 0.5-128 | 32 | 64 | Iran | 2019-2020 | *(Daneshfar et al., 2022)* |
|  | 1 | BMD | 16 |  |  | Turkey | 2010- 2013 | *(Senol et al., 2022)* |
|  | 48 | BMD | 1-64 | 16 | 32 | Iran | 2014-2014 | *(Heidarieh et al., 2016)* |
| *M. virginiense* | 1 | BMD | 64 |  |  | China | 2016-2021 | *(Zhang et al., 2022)* |
|  | 1 | BMD | 4 |  |  | China | 2016-2021 | *(Zhang et al., 2022)* |
|  | 1 | BMD | 64 |  |  | China | 2016-2021 | *(Zhang et al., 2022)* |
| *M. phocaicum* | 85 | BMD |  | 2 | 8 | USA | 2018-2020 | *(Hunkins et al., 2023)* |
| *M. terrae or M. nonchromogenicum* | 11 | BMD | ≤2-＞32 | 16 | 32 | USA |  | *(Brown-Elliott et al., 2003)* |
| *M. triplex* | 10 | BMD | 2-16 | ≤4 | 8 | USA |  | *(Brown-Elliott et al., 2003)* |
| ***Nocardia*** *N. abscessus* | 5 | BMD |  |  |  | Spain | 2006-2018 | *(Galar et al., 2021)* |
|  | 1 | BMD |  |  |  | China | 2017-2019 | *(Lu et al., 2020)* |
|  | 3 | BMD | 0.19-0.25 |  |  | Italy | 2011-2015 | *(Mazzaferri et al., 2018)* |
|  | 5 | BMD | ≤1-2 | ≤1 | 2 | China |  | *(Wei et al., 2021)* |
|  | 2 | BMD | 0.5-2 |  |  | China | 2011-2017 | *(Wei et al., 2017)* |
|  | 1 |  |  |  |  | USA | 1998-2018 | *(Harris et al., 2021)* |
| *N. abscessus complex* | 152 | DD |  |  |  | France | 2010-2015 | *(Lebeaux et al., 2019)* |
|  | 54 | BMD | ≤1-2 | ≤1 | ≤1 | China | 2009-2021 | *(Wang et al., 2022)* |
|  | 18 | BMD | 0.25-4 | 2 | 4 | Japan |  | *(Toyokawa et al., 2021)* |
|  | 2 |  |  |  |  | USA | 1998-2018 | *(Harris et al., 2021)* |
| *N. beijingensis* | 7 |  |  |  |  | USA | 1998-2018 | *(Harris et al., 2021)* |
|  | 1 | BMD |  |  |  | Spain | 2006-2018 | *(Galar et al., 2021)* |
|  | 3 | BMD | 1 |  |  | China | 2011-2017 | *(Wei et al., 2017)* |
|  | 6 | BMD |  |  |  | Australia | 2014-2018 | *(Davidson et al., 2020)* |
|  | 9 |  |  | 1 | 2 | China | 2012-2020 | *(Kuo et al., 2022)* |
| *N. arthritidis* | 2 | BMD | 1-2 |  |  | China | 2011-2020 | *(Lao et al., 2022)* |
|  | 1 | BMD |  |  |  | Spain | 2006-2018 | *(Galar et al., 2021)* |
| *N. asiatica* | 4 | BMD | 1 | 1 | 1 | China | 2011-2020 | *(Lao et al., 2022)* |
|  | 3 |  |  | 1 | 1 | China | 2012-2020 | *(Kuo et al., 2022)* |
|  | 4 | BMD | ≤1-2 | ≤1 | 2 | China |  | *(Wei et al., 2021)* |
| *N. pneumoniae* | 1 | BMD |  |  |  | Spain | 2006-2018 | *(Galar et al., 2021)* |
| *N. brasiliensis* | 15 |  |  |  |  | USA | 1998-2018 | *(Harris et al., 2021)* |
|  | 13 |  |  | 2 | 4 | China | 2012-2020 | *(Kuo et al., 2022)* |
|  | 28 | BMD | 1-4 | 2 | 2 | China | 2011-2020 | *(Lao et al., 2022)* |
|  | 48 | DD |  |  |  | France | 2010-2015 | *(Lebeaux et al., 2019)* |
|  | 1 | BMD |  |  |  | Spain | 2006-2018 | *(Galar et al., 2021)* |
|  | 2 | BMD |  |  |  | Australia | 2014-2018 | *(Davidson et al., 2020)* |
|  | 14 | BMD | 4-8 | 4 | 8 | Japan |  | *(Toyokawa et al., 2021)* |
|  | 2 | BMD | 2-4 |  |  | China | 2017-2019 | *(Yi et al., 2019)* |
|  | 3 | BMD |  |  |  | China | 2017-2019 | *(Lu et al., 2020)* |
|  | 18 | BMD | ≤1-2 | ≤1 | 2 | China | 2009-2021 | *(Wang et al., 2022)* |
|  | 21 | BMD | 1-4 | 2 | 2 | USA | 2014-2015 | *(Brown-Elliott & Wallace Jr, 2017)* |
|  | 1 | BMD | ≤1 |  |  | China |  | *(Wei et al., 2021)* |
|  | 31 | BMD | 0.12-2 | 0.5 | 1 |  |  | *(Vera-Cabrera et al., 2006)* |
| *N. cyriacigeorgica* | 15 |  |  |  |  | USA | 1998-2018 | *(Harris et al., 2021)* |
|  | 2 | BMD | 2 |  |  | USA | 2012-2018 | *(Goodlet et al., 2021)* |
|  | 95 | DD |  |  |  | France | 2010-2015 | *(Lebeaux et al., 2019)* |
|  | 17 | BMD |  |  |  | Spain | 2006-2018 | *(Galar et al., 2021)* |
|  | 5 | BMD |  |  |  | Australia | 2014-2018 | *(Davidson et al., 2020)* |
|  | 27 | BMD | 2-4 | 4 | 4 | Japan |  | *(Toyokawa et al., 2021)* |
|  | 6 | BMD | ≤1-16 |  |  | China | 2017-2019 | *(Yi et al., 2019)* |
|  | 7 | BMD |  |  |  | China | 2017-2019 | *(Lu et al., 2020)* |
|  | 126 | BMD | ≤1-4 | ≤1 | 2 | China | 2009-2021 | *(Wang et al., 2022)* |
|  | 29 | BMD | 0.25-2 | 2 | 4 | USA | 2014-2015 | *(Brown-Elliott & Wallace Jr, 2017)* |
|  | 2 | BMD | 0.094-1 |  |  | Italy | 2011-2015 | *(Mazzaferri et al., 2018)* |
|  | 33 | BMD | ≤1-4 | 2 | 2 | China |  | *(Wei et al., 2021)* |
|  | 13 | BMD | 1-2 |  |  | China | 2011-2017 | *(Wei et al., 2017)* |
|  | 16 | BMD | 2-4 | 2 | 4 | China | 2011-2020 | *(Lao et al., 2022)* |
|  | 25 |  |  | 2 | 2 | China | 2012-2020 | *(Kuo et al., 2022)* |
| *N. farcinica* | 11 |  |  |  |  | USA | 1998-2018 | *(Harris et al., 2021)* |
|  | 7 | BMD | 2-4 |  |  | USA | 2012-2018 | *(Goodlet et al., 2021)* |
|  | 149 | DD |  |  |  | France | 2010-2015 | *(Lebeaux et al., 2019)* |
|  | 4 | BMD |  |  |  | Australia | 2014-2018 | *(Davidson et al., 2020)* |
|  | 8 | BMD | ≤1-2 |  |  | China | 2017-2019 | *(Yi et al., 2019)* |
|  | 18 |  |  | 2 | 4 | China | 2012-2020 | *(Kuo et al., 2022)* |
|  | 1 | BMD |  |  |  | China | 2017-2019 | *(Lu et al., 2020)* |
|  | 176 | BMD | ≤1-4 | 2 | 2 | China | 2009-2021 | *(Wang et al., 2022)* |
|  | 17 | BMD | 1-4 | 2 | 4 | USA | 2014-2015 | *(Brown-Elliott & Wallace Jr, 2017)* |
|  | 3 | BMD | 0.064-2 |  |  | Italy | 2011-2015 | *(Mazzaferri et al., 2018)* |
|  | 6 | BMD |  |  |  | Spain | 2006-2018 | *(Galar et al., 2021)* |
|  | 24 | BMD | 2-4 | 2 | 4 | China | 2011-2020 | *(Lao et al., 2022)* |
|  | 20 | BMD | ≤1-4 | 2 | 4 | China |  | *(Wei et al., 2021)* |
|  | 36 | BMD |  | 2 | 2 | China | 2018-2019 | *(Li et al., 2022)* |
|  | 1 | BMD | 0.25 |  |  | Italy | 2011-2015 | *(Mazzaferri et al., 2018)* |
|  | 6 | BMD | 1-4 |  |  | China | 2011-2017 | *(Wei et al., 2017)* |
| *N. farcinica complex* | 37 | BMD | 0.5-4 | 4 | 4 | Japan |  | *(Toyokawa et al., 2021)* |
| *N. nova* | 11 |  |  |  |  | USA | 1998-2018 | *(Harris et al., 2021)* |
|  | 1 | BMD | ≤1 |  |  | China | 2017-2019 | *(Yi et al., 2019)* |
|  | 6 | BMD | 2 | 2 | 2 | China | 2011-2020 | *(Lao et al., 2022)* |
|  | 1 | BMD | ≤1 |  |  | China |  | *(Wei et al., 2021)* |
|  | 1 | BMD | 2 |  |  | China | 2011-2017 | *(Wei et al., 2017)* |
| *N. nova complex* | 11 | BMD | ≤1-2 | ≤1 | 2 | China | 2009-2021 | *(Wang et al., 2022)* |
|  | 13 | BMD | 0.25-2 | 1 | 2 | USA | 2014-2015 | *(Brown-Elliott & Wallace Jr, 2017)* |
|  | 145 | DD |  |  |  | France | 2010-2015 | *(Lebeaux et al., 2019)* |
|  | 23 | BMD | ≤0.25-4 | 2 | 4 | Japan |  | *(Toyokawa et al., 2021)* |
| *N. veterana* | 2 | BMD | 1-16 |  |  | China | 2011-2020 | *(Lao et al., 2022)* |
|  | 1 | BMD |  |  |  | Spain | 2006-2018 | *(Galar et al., 2021)* |
|  | 2 |  |  |  |  | USA | 1998-2018 | *(Harris et al., 2021)* |
| *N. veteran/elegans* | 4 | BMD |  |  |  | Australia | 2014-2018 | *(Davidson et al., 2020)* |
| *N. elegans* | 1 | BMD | 2 |  |  | China | 2011-2020 | *(Lao et al., 2022)* |
| *N. aobensis* | 1 | BMD | 2 |  |  | China |  | *(Wei et al., 2021)* |
| *N. cerradoensis* | 1 |  |  |  |  | China | 2012-2020 | *(Kuo et al., 2022)* |
| *N. kruczakiae* | 1 |  |  |  |  | USA | 1998-2018 | *(Harris et al., 2021)* |
| *N. otitidiscaviarium* | 2 | BMD | ≤1-2 |  |  | China | 2017-2019 | *(Yi et al., 2019)* |
|  | 1 |  |  |  |  | USA | 1998-2018 | *(Harris et al., 2021)* |
|  | 1 | BMD | 2 |  |  | USA | 2012-2018 | *(Goodlet et al., 2021)* |
|  | 2 | BMD |  |  |  | Spain | 2006-2018 | *(Galar et al., 2021)* |
|  | 11 | BMD | 1-8 | 4 | 4 | Japan |  | *(Toyokawa et al., 2021)* |
|  | 11 | BMD |  |  |  | China | 2017-2019 | *(Lu et al., 2020)* |
|  | 26 | BMD | ≤1-2 | ≤1 | 2 | China | 2009-2021 | *(Wang et al., 2022)* |
|  | 7 | BMD | ≤1-4 | ≤1 | 4 | China |  | *(Wei et al., 2021)* |
|  | 1 |  |  |  |  | China | 2012-2020 | *(Kuo et al., 2022)* |
|  | 4 | BMD | 2-4 | 2 | 4 | China | 2011-2020 | *(Lao et al., 2022)* |
|  | 1 | BMD | 0.5 |  |  | China | 2011-2017 | *(Wei et al., 2017)* |
| *N. pseudobrasiliensis* | 2 | BMD |  |  |  | Spain | 2006-2018 | *(Galar et al., 2021)* |
|  | 2 | BMD |  |  |  | China | 2009-2021 | *(Wang et al., 2022)* |
|  | 5 | BMD | 0.25-2 | 0.5 |  | USA | 2014-2015 | *(Brown-Elliott & Wallace Jr, 2017)* |
|  | 5 |  |  |  |  | USA | 1998-2018 | *(Harris et al., 2021)* |
| *N. transvalensis* | 2 | BMD | ≤1 |  |  | USA | 2012-2018 | *(Goodlet et al., 2021)* |
| *N. transvalensis complex* | 49 | DD |  |  |  | France | 2010-2015 | *(Lebeaux et al., 2019)* |
|  | 11 | BMD | ≤1-2 | ≤1 | ≤1 | China | 2009-2021 | *(Wang et al., 2022)* |
|  | 6 | BMD | 1-4 | 2 | 4 | Japan |  | *(Toyokawa et al., 2021)* |
| *N. transvalensis/ wallacei* | 1 |  |  |  |  | USA | 1998-2018 | *(Harris et al., 2021)* |
| *N. wallacei* | 1 |  |  |  |  | USA | 1998-2018 | *(Harris et al., 2021)* |
|  | 3 | BMD | ≤1-2 |  |  | USA | 2012-2018 | *(Goodlet et al., 2021)* |
|  | 1 | BMD |  |  |  | Spain | 2006-2018 | *(Galar et al., 2021)* |
|  | 5 | BMD | 0.5-2 | 1 |  | USA | 2014-2015 | *(Brown-Elliott & Wallace Jr, 2017)* |
|  | 2 | BMD |  |  |  | China | 2017-2019 | *(Lu et al., 2020)* |
|  | 4 | BMD | ≤1-2 | ≤1 | 2 | China |  | *(Wei et al., 2021)* |
|  | 2 | BMD | 0.5-1 |  |  | China | 2011-2017 | *(Wei et al., 2017)* |
| *N. asteroides* | 1 | BMD | 4 |  |  | Japan |  | *(Toyokawa et al., 2021)* |
|  | 1 | BMD |  |  |  | Spain | 2006-2018 | *(Galar et al., 2021)* |
|  | 2 |  |  |  |  | China | 2012-2020 | *(Kuo et al., 2022)* |
| *N. asteroides (probably cyriacigeorgica)* | 4 |  |  |  |  | USA | 1998-2018 | *(Harris et al., 2021)* |
| *N. asteroides complex* | 2 |  |  |  |  | USA | 1998-2018 | *(Harris et al., 2021)* |
| *N. yamanashiensis* | 1 |  |  |  |  | USA | 1998-2018 | *(Harris et al., 2021)* |
|  | 1 | BMD |  |  |  | Australia | 2014-2018 | *(Davidson et al., 2020)* |
|  | 1 | BMD | 1 |  |  | Japan |  | *(Toyokawa et al., 2021)* |
| *N. puris* | 7 | BMD | ≤1-2 | ≤1 | 2 | China | 2009-2021 | *(Wang et al., 2022)* |
|  | 1 | BMD | 1 |  |  | China | 2011-2020 | *(Lao et al., 2022)* |
|  | 2 | BMD | ≤1-2 |  |  | China |  | *(Wei et al., 2021)* |
| *N. amikacinitolerans* | 2 |  |  |  |  | USA | 1998-2018 | *(Harris et al., 2021)* |
|  | 1 |  |  |  |  | China | 2012-2020 | *(Kuo et al., 2022)* |
| *N. niwae* | 2 |  |  |  |  | USA | 1998-2018 | *(Harris et al., 2021)* |
| *N. thailandica* | 2 | BMD | 4 |  |  | Japan |  | *(Toyokawa et al., 2021)* |
| *N. asteroldes* | 2 | BMD |  |  |  | China | 2017-2019 | *(Lu et al., 2020)* |
| *N. concava* | 2 |  |  |  |  | China | 2012-2020 | *(Kuo et al., 2022)* |
| *N. brevicatena* | 1 | BMD |  |  |  | Australia | 2014-2018 | *(Davidson et al., 2020)* |
| *N. crassostreae* | 1 |  |  |  |  | China | 2012-2020 | *(Kuo et al., 2022)* |
| *N. vinacea* | 1 | BMD | 2 |  |  | Japan |  | *(Toyokawa et al., 2021)* |
| *N. higoensis/shimofusensis* | 1 |  |  |  |  | USA | 1998-2018 | *(Harris et al., 2021)* |
| *N. mexicana* | 1 | BMD | 4 |  |  | Japan |  | *(Toyokawa et al., 2021)* |
| *N. niigatensis* | 1 |  |  |  |  | China | 2012-2020 | *(Kuo et al., 2022)* |
| *N. takedensis* | 1 | BMD | 2 |  |  | Japan |  | *(Toyokawa et al., 2021)* |
| *N. neocaledoniensis* | 1 | BMD | 2 |  |  | China | 2011-2020 | *(Lao et al., 2022)* |

Abbreviations: N, number of strains; MIC, minimum inhibitory concentration; DD, disk diffusion; BMD, broth microdilution; ADM, agar dilution method; RPM, radiometric proportion method; MABA, microplate alamar Blue assay; MDR, multidrug-resistant; XDR, extensively drug-resistant.

**Table 4 In vitro activity of linezolid against Corynebacterium, Anaerobe and other pathogens (MIC, μg/mL).**

| **Organism** | **N** | **Method** | **MIC range** | **MIC_50_** | **MIC_90_** | **Country** | **Time of study** | **Reference** |
| --- | --- | --- | --- | --- | --- | --- | --- | --- |
| ***Corynebacterium*** *Corynebacterium spp.* | 48 | BMD | 0.12-1 | 0.25 | 0.5 | USA |  | *(Jones et al., 2002)* |
|  | 34 | BMD |  | 0.25 | 0.5 | whole world | 2011-2013 | *(Mendes et al., 2015)* |
| *Corynebacterium group* | 10 | ADM | 0.125-0.5 | 0.25 | 0.5 | USA | 1996-2002 | *(Goldstein et al., 2004)* |
| *Corynebacterium spp* | 50 | BMD | 0.12-2 | 0.5 | 1 | France | 2013-2016 | *(Ract et al., 2017)* |
| *Corynebacterium spp.* | 20 | ADM | 1-8 | 2 | 8 | USA |  | *(Goldstein et al., 2006)* |
| *C. amycolatum* | 35 | ADM | 0.063-1 | 0.25 | 1 | Argentina |  | *(Barberis et al., 2018)* |
|  | 33 | E-test | 0.064-0.25 | 0.19 | 0.25 | Spain |  | *(Fernandez-Roblas et al., 2009)* |
|  | 58 | ADM | ≤ 0.1-2 | 0.2 | 0.2 | Spain |  | *(Sánchez Hernández et al., 2003)* |
|  | 60 | ADM | ≤ 0.12-0.5 | 0.25 | 0.25 |  | 2001-2004 | *(Gómez-Garcés et al., 2007)* |
|  | 20 | ADM | 0.25-0.5 | 0.5 | 0.5 | USA |  | *(Goldstein et al., 2006)* |
|  | 10 | ADM | 0.25-0.5 | 0.5 | 0.5 | USA | 1996-2002 | *(Goldstein et al., 2004)* |
|  | 5 |  | 0.4-4 |  |  | UK | 1999-2002 | *(Johnson et al., 2003)* |
|  | 1 | BMD | ≤ 0.5 | ≤ 0.5 | ≤ 0.5 |  | 2013- 2022 | *(Abe et al., 2021)* |
|  | 190 | BMD | ≤ 1 | ≤ 1 | ≤ 1 | Canada | 2011-2016 | *(Neemuchwala et al., 2018)* |
|  | 5 | BMD |  | 1 | 1 | China | 2018-2020 | *(Sun et al., 2022)* |
| *C. jeikeium* | 25 | E-test | 0.064-0.38 | 0.25 | 0.5 | Spain |  | *(Fernandez-Roblas et al., 2009)* |
|  | 30 | ADM | ≤ 0.12-1 | 0.5 | 0.5 |  | 2001-2004 | *(Gómez-Garcés et al., 2007)* |
|  | 31 | ADM | 0.2-1 | 1 | 1 | Spain |  | *(Sánchez Hernández et al., 2003)* |
|  | 11 | ADM | 0.5-0.5 | 0.5 | 0.5 | USA | 1996-2002 | *(Goldstein et al., 2004)* |
|  | 72 |  | 0.5-2 |  |  | UK | 1999-2002 | *(Johnson et al., 2003)* |
|  | 19 | BMD | ≤ 0.5 | ≤ 0.5 | ≤ 0.5 | Japan | 2013- 2019 | *(Johnson et al., 2003)* |
|  | 76 | BMD | ≤ 1 | ≤ 1 | ≤ 1 | Canada | 2011-2016 | *(Neemuchwala et al., 2018)* |
|  | 4 | BMD |  | 1 | 2 | China | 2018-2020 | *(Sun et al., 2022)* |
| *C. striatum* | 55 | ADM | ≤ 0.063-2 | 0.125 | 0.25 | Argentina |  | *(Barberis et al., 2018)* |
|  | 30 | ADM | ≤ 0.12-1 | 0.25 | 0.5 |  | 2001-2004 | *(Gómez-Garcés et al., 2007)* |
|  | 11 | E-test | 0.125-0.75 | 0.38 | 0.5 | Spain |  | *(Fernandez-Roblas et al., 2009)* |
|  | 5 |  | 0.5 |  |  | UK | 1999-2002 | *(Johnson et al., 2003)* |
|  | 124 | BMD | ≤ 0.5-0.5 | ≤ 0.5 | ≤ 0.5 | Japan | 2013-2018 | *(Abe et al., 2021)* |
|  |  |  | ≤ 1 | ≤ 1 | ≤ 1 | Korea | 2016 | *(Suh et al., 2019)* |
|  | 931 | BMD | ≤ 1 | ≤ 1 | ≤ 1 | Canada | 2011-2016 | *(Neemuchwala et al., 2018)* |
|  | 410 | BMD | ≤ 2 | <0.5 | <0.5 | China | 2013-2019 | *(Wang et al., 2021)* |
|  | 15 | BMD |  | 0.5 | 0.5 | China | 2018-2020 | *(Sun et al., 2022)* |
|  | 7 | ADM | 0.12-0.25 |  |  | France | 2007-2010 | *(Nhan et al., 2012)* |
| *C. urealyticum* | 40 | E-test | 0.015-256 | 0.75 | 1 | Spain | 2005-2017 | *(Chapartegui-González et al., 2020)* |
|  | 34 | E-test | 0.064-0.5 | 0.19 | 0.38 | Spain |  | *(Fernandez-Roblas et al., 2009)* |
|  | 10 | ADM | 0.25-1 | 0.5 | 0.5 |  | 2001-2004 | *(Gómez-Garcés et al., 2007)* |
|  | 64 | ADM | 0.5-1 | 0.5 | 0.5 | Spain |  | *(Sánchez Hernández et al., 2003)* |
|  | 52 | BMD | ≤ 1 | ≤ 1 | ≤ 1 | Canada | 2011-2016 | *(Neemuchwala et al., 2018)* |
|  | 8 | BMD |  | 0.12 | 1 | China | 2018-2020 | *(Sun et al., 2022)* |
| *C. coyleae* | 13 | E-test | 0.047-0.25 | 0.19 | 0.19 | Spain |  | *(Fernandez-Roblas et al., 2009)* |
| *C. aurimucosum* | 11 | E-test | 0.064-0.75 | 0.38 | 0.5 | Spain |  | *(Fernandez-Roblas et al., 2009)* |
| *C. pseudodiphtheriticum* | 13 | ADM | 0.063-0.25 | 0.063 | 0.125 | Argentina |  | *(Barberis et al., 2018)* |
| *C. glucuronolyticum* | 10 | ADM | 0.063-1 | 0.5 | 1 | Argentina |  | *(Barberis et al., 2018)* |
| *C. afermentans* | 8 | E-test | 0.094-0.19 | 0.094 | 0.19 | Spain |  | *(Fernandez-Roblas et al., 2009)* |
| *C. kroppenstedtii* | 90 | BMD | 0.25-2 | 0.5 | 1 | China | 2018-2019 | *(Zhang et al., 2023)* |
| *C. pseudodiphtheriticum* | 17 | ADM | 0.25 | 0.25 | 0.12-0.5 | France | 2007-2009 | *(Nhan et al., 2012)* |
| *C. accolens* | 1 |  | 0.5 |  |  | UK | 1999-2002 | *(Johnson et al., 2003)* |
| *C. aurimucosum* | 52 | DD | 0.5 | 1 | 0.12-2 | France | 2010-2019 | *(Lefèvre et al., 2021)* |
| *C. gluculonolyticum* | 1 | BMD | ≤ 0.5 | ≤ 0.5 | ≤ 0.5 |  | 2013- 2021 | *(Abe et al., 2021)* |
| *C. resistens* | 2 | BMD | ≤ 0.5 | ≤ 0.5 | ≤ 0.5 |  | 2013- 2020 | *(Abe et al., 2021)* |
| *C. aurimucosum* | 51 | BMD | ≤ 1 | ≤ 1 | ≤ 1 | Canada | 2011-2016 | *(Neemuchwala et al., 2018)* |
| *C. minutissimum* | 76 | BMD | ≤ 1-＞8 | ≤ 1 | ≤ 1 | Canada | 2011-2016 | *(Neemuchwala et al., 2018)* |
| *C. afermentans subsp afermentans* | 89 | BMD | ≤ 1-2 | ≤ 1 | ≤ 1 | Canada | 2011-2016 | *(Neemuchwala et al., 2018)* |
| *C. coyleae* | 58 | BMD | ≤ 1-2 | ≤ 1 | ≤ 1 | Canada | 2011-2016 | *(Neemuchwala et al., 2018)* |
| *C. pseudodiphtheriticum /propinquum* | 216 | BMD | ≤ 1-2 | ≤ 1 | ≤ 1 | Canada | 2011-2016 | *(Neemuchwala et al., 2018)* |
| *C. afermentans* | 1 |  | 1 |  |  | UK | 1999-2002 | *(Johnson et al., 2003)* |
| *C. macginleyi* | 1 |  | 1 |  |  | UK | 1999-2002 | *(Johnson et al., 2003)* |
| *C. aquaticum* | 2 |  | 2 |  |  | UK | 1999-2002 | *(Johnson et al., 2003)* |
| *C. accolens* | 3 | ADM |  |  | 0.25 | France | 2007-2011 | *(Nhan et al., 2012)* |
| *C. glucuronolyticum* | 9 | BMD |  | 0.5 | 2 | China | 2018-2020 | *(Sun et al., 2022)* |
| *C. tuberculostearicum* | 4 | BMD |  | 2 | 2 | China | 2018-2020 | *(Sun et al., 2022)* |
| **Anaerobe** *Clostridium spp.* | 20 | ADM | ≤0.06-4 | 2 | 4 | France | 1999-2000 | *(Behra-Miellet et al., 2003)* |
|  | 38 | E-test | 0.25 -16 | 2 | 4 | Belgian | 2011-2012 | *(Wybo et al., 2014)* |
|  | 25 | ADM | 0.25-4 | 1 | 4 |  | 2000-2003 | *(Citron et al., 2003)* |
|  | 10 | ADM | 1-4 | 2 | 4 | India |  | *(Mathur et al., 2011)* |
|  | 4 | ADM | 2-4 |  |  | Korea | 2011-2014 | *(Lee et al., 2015)* |
| *Clostridium spp.* | 20 | ADM | 1-8 | 2 | 8 | USA |  | *(Goldstein et al., 2006)* |
| *Clostridium difficile* | 114 | ADM | 0.125-8 | 2 | 4 | Sweden | 2008-2011 | *(Rashid et al., 2014)* |
|  | 94 | ADM and E-test | 0.5-4 | 2 | 2 | Kuwait |  | *(Phillips et al., 2003)* |
|  | 15 |  | 0.5-4 | 2 | 2 | Korea | 2000-2001 | *(Yong et al., 2004)* |
|  | 12 | ADM | 1-2 | 2 | 2 | USA | 1999-2002 | *(Ednie et al., 2002)* |
|  | 18 | ADM | 2-16 | 2 | 16 |  | 2000-2003 | *(Citron et al., 2003)* |
|  | 50 | ADM | 2-16 | 4 | 8 | India |  | *(Mathur et al., 2011)* |
|  | 10 | ADM | 2-2 | 2 | 2 | USA | 2015-2017 | *(Goldstein et al., 2020)* |
|  | 14 | ADM | 2-8 | 2 | 8 |  | 1996-2002 | *(Goldstein et al., 2005)* |
|  | 14 | ADM | 2-8 | 2 | 8 | USA | 1996-2002 | *(Goldstein et al., 2004)* |
| *Clostridium perfringens* | 20 | ADM | 1-2 | 2 | 2 | USA | 1999-2002 | *(Ednie et al., 2002)* |
|  | 15 | ADM | 1-2 | 2 | 2 | Korea | 2002-2004 | *(Yum et al., 2010)* |
|  | 50 | ADM | 1-4 | 2 | 2 | Swedenwere |  | *(Edlund et al., 1999)* |
|  | 11 | ADM | 1-4 | 2 | 2 |  | 2000-2003 | *(Citron et al., 2003)* |
|  | 10 | ADM | 1-16 | 2 | 2 | USA | 2015-2017 | *(Goldstein et al., 2020)* |
|  | 12 | ADM | 2 | 2 | 2 |  | 1996-2002 | *(Goldstein et al., 2005)* |
|  | 17 |  | 2 | 2 | 2 | Korea | 2000-2001 | *(Yong et al., 2004)* |
|  | 12 | ADM | 2-2 | 2 | 2 | USA | 1996-2002 | *(Goldstein et al., 2004)* |
| *Clostridium clostridioforme* | 15 | ADM | 2-4 | 2 | 4 |  | 1996-2002 | *(Goldstein et al., 2005)* |
|  | 15 | ADM | 2-4 | 2 | 4 | USA | 1996-2002 | *(Goldstein et al., 2004)* |
|  | 10 | ADM | 2-4 | 2 | 4 |  | 2000-2003 | *(Citron et al., 2003)* |
| *Clostridium clostridioforme grp.* | 10 | ADM | 2-16 | 4 | 16 | USA | 2015-2017 | *(Goldstein et al., 2020)* |
| *Clostridium innocuum* | 15 | ADM | 2-4 | 2 | 2 | USA | 1996-2002 | *(Goldstein et al., 2004)* |
|  | 15 | ADM | 2-4 | 2 | 4 |  | 1996-2002 | *(Goldstein et al., 2005)* |
|  | 19 | ADM | 2-4 | 4 | 4 |  | 2000-2003 | *(Citron et al., 2003)* |
|  | 10 | ADM | 2-＞16 | 4 | 4 | USA | 2015-2017 | *(Goldstein et al., 2020)* |
| *Clostridium ramosum* | 15 | ADM | 4-8 | 8 | 8 |  | 2000-2003 | *(Citron et al., 2003)* |
|  | 16 | ADM | 4-8 | 8 | 8 |  | 1996-2002 | *(Goldstein et al., 2005)* |
|  | 16 | ADM | 4-8 | 8 | 8 | USA | 1996-2002 | *(Goldstein et al., 2004)* |
|  | 10 | ADM | 8-16 | 8 | 8 | USA | 2015-2017 | *(Goldstein et al., 2020)* |
| *Clostridium species* | 39 |  | 0.5-8 | 2 | 4 | USA |  | *(Molitoris et al., 2006)* |
| *Clostridium bifermentans Clostridium sordellii group* | 10 | ADM | 1-1 | 1 | 1 |  | 2000-2003 | *(Citron et al., 2003)* |
| *Clostridium dijiicile* | 50 | ADM | 1-2 | 1 | 2 | Swedenwere |  | *(Edlund et al., 1999)* |
| *Clostridium paraputrificum Clostridium tertium group* | 10 | ADM | 1-8 | 4 | 4 |  | 2000-2003 | *(Citron et al., 2003)* |
| *Clostridium cadaveris* | 10 | ADM | 2-4 | 4 | 4 |  | 2000-2003 | *(Citron et al., 2003)* |
| *Actinomyces spp.* | 22 | ADM | 0.5-8 | 0.5 | 0.5 |  | 2000-2003 | *(Citron et al., 2003)* |
| *Actinomyces israelii* | 13 | ADM | 0.25-16 | 0.5 | 16 |  | 1996-2002 | *(Goldstein et al., 2005)* |
|  | 13 | ADM | 0.25-16 | 0.5 | 16 | USA | 1996-2002 | *(Goldstein et al., 2004)* |
| *Actinomyces meyeri-A. turicensis group* | 12 | ADM | 0.125-1 | 0.5 | 0.5 | USA | 1996-2002 | *(Goldstein et al., 2004)* |
| *Actinomyces meyeri-Actinomyces turicensis group* | 12 | ADM | 0.12-1 | 0.5 | 0.5 |  | 1996-2002 | *(Goldstein et al., 2005)* |
| *Actinomyces odontolyticus* | 10 | ADM | 0.5-1 | 0.5 | 0.5 |  | 1996-2002 | *(Goldstein et al., 2005)* |
|  | 10 | ADM | 0.5-1 | 0.5 | 0.5 | USA | 1996-2002 | *(Goldstein et al., 2004)* |
| *Actinomyces viscosus* | 10 | ADM | 0.5 | 0.5 | 0.5 |  | 1996-2002 | *(Goldstein et al., 2005)* |
|  | 10 | ADM | 0.5-0.5 | 0.5 | 0.5 | USA | 1996-2002 | *(Goldstein et al., 2004)* |
| *Bifidobacterium spp.* | 13 | ADM | 0.25-2 | 1 | 1 |  | 2000-2003 | *(Citron et al., 2003)* |
| *Propionibacterium spp.* | 52 | BMD | 0.12-2 | 0.5 | 1 | France | 2013-2016 | *(Ract et al., 2017)* |
| *Propionibacterium spp.* | 13 | ADM | 0.25-0.5 | 0.5 | 0.5 | France | 1999-2000 | *(Behra-Miellet et al., 2003)* |
|  | 15 | ADM | 0.25-1 | 5 | 1 |  | 2000-2003 | *(Citron et al., 2003)* |
| *Propionibacterium acnes* | 10 | ADM | 0.25-0.5 | 0.5 | 0.5 | India |  | *(Mathur et al., 2011)* |
|  | 10 | ADM | 0.25-0.5 | 0.5 | 0.5 |  | 1996-2002 | *(Goldstein et al., 2005)* |
|  | 16 | ADM | 0.25-0.5 | 0.5 | 0.5 | USA | 1999-2002 | *(Ednie et al., 2002)* |
|  | 12 | ADM | 0.25-0.5 | 0.5 | 0.5 | USA | 1996-2002 | *(Goldstein et al., 2004)* |
|  | 30 | ADM | 0.25-1 | 0.5 | 0.5 | Swedenwere |  | *(Edlund et al., 1999)* |
|  | 304 | ADM | 0.25-2 | 0.5 | 1 | Europe |  | *(Oprica & Nord, 2005)* |
| *Propionibacterium avidum* | 12 | ADM | ≤0.5 | ≤0.5 | ≤0.5 |  | 1996-2002 | *(Goldstein et al., 2005)* |
|  | 12 | ADM | 0.5-1 | 0.5 | 0.5 | USA | 1996-2002 | *(Goldstein et al., 2004)* |
| *Propionibacterium granulosum* | 10 | ADM | 0.25-0.5 | 0.25 | 0.25 |  | 1996-2002 | *(Goldstein et al., 2005)* |
|  | 10 | ADM | 0.25-0.5 | 0.25 | 0.25 | USA | 1996-2002 | *(Goldstein et al., 2004)* |
| *Lactobacillus spp.* | 37 | ADM | 0.5-16 | 4 | 8 |  | 2000-2003 | *(Citron et al., 2003)* |
|  | 16 | ADM | 1-8 | 4 | 8 | USA | 1996-2002 | *(Goldstein et al., 2004)* |
|  | 16 | ADM | 1-8 | 4 | 8 |  | 1996-2002 | *(Goldstein et al., 2005)* |
| *Lactobacillus casei* | 6 | ADM | 4 | 4 |  |  | 1996-2002 | *(Goldstein et al., 2005)* |
|  | 6 | ADM | 4 | 4 |  | USA | 1996-2002 | *(Goldstein et al., 2004)* |
| *Lactobacillus plantarum* | 10 | ADM | 4-8 | 8 | 8 |  | 1996-2002 | *(Goldstein et al., 2005)* |
|  | 10 | ADM | 4-8 | 8 | 8 | USA | 1996-2002 | *(Goldstein et al., 2004)* |
| *Eubacterium group* | 13 | ADM | 0.5-2 | 2 | 2 |  | 1996-2002 | *(Goldstein et al., 2005)* |
|  | 13 | ADM | 0.5-2 | 2 | 2 | USA | 1996-2002 | *(Goldstein et al., 2004)* |
|  | 31 | ADM | 0.06-8 | 1 | 8 |  | 2000-2003 | *(Citron et al., 2003)* |
| *Eubacterium lentum* | 10 | ADM | 0.5-2 | 2 | 2 | USA | 1996-2002 | *(Goldstein et al., 2004)* |
|  | 9 | ADM | 1-2 | 2 |  |  | 1996-2002 | *(Goldstein et al., 2005)* |
|  | 17 | ADM | 1-2 | 1 | 2 |  | 2000-2003 | *(Citron et al., 2003)* |
| *Eubacterium limosum* | 10 | ADM | 1-4 | 2 | 4 |  | 1996-2002 | *(Goldstein et al., 2005)* |
|  | 10 | ADM | 1-4 | 2 | 4 | USA | 1996-2002 | *(Goldstein et al., 2004)* |
| *Peptostreptococcus spp.* | 59 | ADM | 0.25-2 | 0.5 | 1 | Korea | 2002-2004 | *(Yum et al., 2010)* |
|  | 13 | ADM | 0.5-16 | 1 | 2 |  | 2000-2003 | *(Citron et al., 2003)* |
|  | 27 | ADM | 0.5-2 | 1 | 2 | Korea | 2011-2014 | *(Lee et al., 2015)* |
|  | 10 |  | 0.5-2 | 1 | 2 | USA | 1990-1997 | *(Goldstein et al., 1999)* |
|  | 75 | ADM | 0.5-2.0 | 1 | 2 | USA | 1999-2002 | *(Ednie et al., 2002)* |
|  | 27 |  | 0.5-4 | 1 | 4 | Kuwait |  | *(Phillips et al., 2003)* |
|  | 56 |  | 0.5-8 | 1 | 2 | Korea | 2000-2001 | *(Yong et al., 2004)* |
|  | 15 | ADM | 0.5-8 | 2 | 4 | India |  | *(Mathur et al., 2011)* |
| *Peptostreptococcus anaerobius* | 10 | ADM | 0.5-1 | 0.5 | 0.5 | USA | 2015-2017 | *(Goldstein et al., 2020)* |
|  | 10 | ADM | 0.5-8 | 0.5 | 8 |  | 1996-2002 | *(Goldstein et al., 2005)* |
|  | 10 | ADM | 0.5-8 | 0.5 | 8 | USA | 1996-2002 | *(Goldstein et al., 2004)* |
| *Peptostreptococcus asaccharolyticus* | 10 | ADM | 0.5-1 | 1 | 1 |  | 2000-2003 | *(Citron et al., 2003)* |
| *Finegoldia magna* | 49 | ADM | 0.25-4 | 2 | 2 | French | 2016-2020 | *(Guérin et al., 2021)* |
|  | 11 | ADM | 0.5-2 | 1 | 2 |  | 1996-2002 | *(Goldstein et al., 2005)* |
|  | 11 | ADM | 0.5-2 | 1 | 2 | USA | 1996-2002 | *(Goldstein et al., 2004)* |
|  | 21 | ADM | 0.5-2 | 2 | 2 | Korea | 2011-2014 | *(Lee et al., 2015)* |
|  | 29 | ADM | 0.5-2 | 2 | 2 | USA |  | *(Goldstein et al., 2006)* |
|  | 10 | ADM | 1-2 | 2 | 2 | USA | 2015-2017 | *(Goldstein et al., 2020)* |
| *Peptoniphilus spp.* | 30 | ADM | 0.25-2 | 1 | 2 | French | 2016-2020 | *(Guérin et al., 2021)* |
| *Peptoniphilus asaccharolyticus* | 20 | ADM | 0.25-4 | 0.5 | 1 | USA |  | *(Goldstein et al., 2006)* |
|  | 10 | ADM | 0.5-1 | 0.5 | 1 |  | 1996-2002 | *(Goldstein et al., 2005)* |
|  | 10 | ADM | 0.5-1 | 0.5 | 1 | USA | 1996-2002 | *(Goldstein et al., 2004)* |
| *Micromonas micros* | 11 | ADM | 0.05-1 | 0.5 | 0.5 |  | 1996-2002 | *(Goldstein et al., 2005)* |
|  | 10 | ADM | 0.5-1 | 0.5 | 1 | USA | 1996-2002 | *(Goldstein et al., 2004)* |
| *Parvimonas micra* | 10 | ADM | 0.5-1 | 0.5 | 1 | USA | 2015-2017 | *(Goldstein et al., 2020)* |
|  | 33 | ADM | 0.5-2 | 1 | 1 | French | 2016-2020 | *(Guérin et al., 2021)* |
| *Eggerthella lenta* | 10 | ADM | 1-2 | 1 | 2 | USA | 2015-2017 | *(Goldstein et al., 2020)* |
| *Brevibacterium casei* | 20 | ADM | 0.5-2 | 1 | 2 |  | 2001-2004 | *(Gómez-Garcés et al., 2007)* |
| *Dermabacter hominis* | 20 | ADM | 0.5-2 | 0.5 | 1 |  | 2001-2004 | *(Gómez-Garcés et al., 2007)* |
| *Peptostreptococcus anaerobius* | 11 | ADM | 0.5-2 | 0.5 | 0.5 | French | 2016-2020 | *(Guérin et al., 2021)* |
| *Peptostreptococcus magnus-Peptostreptococcus-microsgroup* | 14 | ADM | 0.5-4 | 1 | 2 |  | 2000-2003 | *(Citron et al., 2003)* |
| *Bacteroides spp.* | 30 | ADM | 2-16 | 4 | 8 | India |  | *(Mathur et al., 2011)* |
| *Bacteroides fragilis* | 455 | ADM | ≤0.5-8 | 2 | 4 | USA | 2010-2012 | *(Snydman et al., 2017)* |
|  | 69 | E-test | 1-16 | 2 | 4 | Belgian | 2011-2012 | *(Wybo et al., 2014)* |
|  | 46 | ADM | 2-16 | 4 | 4 | USA | 2015-2017 | *(Goldstein et al., 2020)* |
|  | 57 |  | 2-16 | 4 | 4 |  |  | *(Goldstein et al., 2017)* |
|  | 57 |  | 2-4 | 4 | 4 | Kuwait |  | *(Phillips et al., 2003)* |
|  | 52 | ADM | 2-4 | 4 | 4 | France | 1999-2000 | *(Behra-Miellet et al., 2003)* |
|  | 30 | ADM | 2-4 | 4 | 4 | Korea | 2002-2004 | *(Yum et al., 2010)* |
|  | 41 |  | 2-8 | 4 | 4 | USA |  | *(Molitoris et al., 2006)* |
|  | 34 |  | 4 | 4 | 4 | Korea | 2000-2001 | *(Yong et al., 2004)* |
|  | 10 | ADM | 4-8 | 4 | 4 | USA | 1999-2002 | *(Ednie et al., 2002)* |
| *Bacteroides fragilis group* | 17 | ADM | 2-4 | 4 | 4 |  | 2000-2003 | *(Citron et al., 2003)* |
| *Bacteroides fragilis group species* | 10 |  | 1-8 | 2 | 4 | USA |  | *(Molitoris et al., 2006)* |
| *B. fragilis group non-B. fragilis* | 15 | ADM | 2-8 | 4 | 8 | USA | 1999-2002 | *(Ednie et al., 2002)* |
| *B. fragilis group other than B. fragilis* | 32 | ADM | 0.5-4 | 4 | 4 | France | 1999-2000 | *(Behra-Miellet et al., 2003)* |
| *Bacteroides and Parabacteroides spp.* | 180 | E-test | 0.5-16 | 2 | 4 | Belgian | 2011-2012 | *(Wybo et al., 2014)* |
| *Bacteroides and Parabacteroides spp. without B. fragilis* | 111 | E-test | 0.5-16 | 2 | 4 | Belgian | 2011-2012 | *(Wybo et al., 2014)* |
| *Bacteroides caccae* | 11 | ADM | 1-4 | 2 | 4 | USA | 2015-2017 | *(Goldstein et al., 2020)* |
|  | 10 |  | 2-8 | 4 | 4 | USA |  | *(Molitoris et al., 2006)* |
| *Bacteroides distasonis* | 1 |  | 4 |  |  |  |  | *(Goldstein et al., 2017)* |
| *Bacteroides distasonis/merdae* | 11 |  | 4-8 | 4 | 8 | USA |  | *(Molitoris et al., 2006)* |
| *Bacteroides ovatus* | 53 | ADM | ≤0.5-4 | 2 | 4 | USA | 2010-2012 | *(Snydman et al., 2017)* |
|  | 10 |  | 2-4 | 4 | 4 | USA |  | *(Molitoris et al., 2006)* |
|  | 11 | ADM | 2-4 | 2 | 2 | USA | 2015-2017 | *(Goldstein et al., 2020)* |
|  | 19 |  | 2-16 | 4 | 4 |  |  | *(Goldstein et al., 2017)* |
| *Bacteroides pyogenes* | 15 | ADM | 2-4 | 2 | 2 |  |  | *(Goldstein et al., 2017)* |
| *Bacteroides stercoris* | 10 |  | 1-16 | 4 | 8 | USA |  | *(Molitoris et al., 2006)* |
| *Bacteroides tectum* | 21 |  | 1-4 | 2 | 2 | USA | 1990-1997 | *(Goldstein et al., 1999)* |
| *Bacteroides thetaiotaomicron* | 135 | ADM | ≤0.5-16 | 2 | 4 | USA | 2010-2012 | *(Snydman et al., 2017)* |
|  | 14 | ADM | 2-4 | 2 | 4 | USA | 2015-2017 | *(Goldstein et al., 2020)* |
|  | 27 |  | 2-8 | 4 | 8 |  |  | *(Goldstein et al., 2017)* |
|  | 15 | ADM | 4 | 4 | 4 | Korea | 2002-2004 | *(Yum et al., 2010)* |
|  | 39 |  | 4-16 | 4 | 8 | USA |  | *(Molitoris et al., 2006)* |
|  | 15 |  | 4-8 | 4 | 8 | Korea | 2000-2001 | *(Yong et al., 2004)* |
| *Bacteroides uniformis* | 26 | ADM | ≤0.5-4 | 2 | 4 | USA | 2010-2012 | *(Snydman et al., 2017)* |
|  | 11 | ADM | 1-2 | 2 | 2 | USA | 2015-2017 | *(Goldstein et al., 2020)* |
|  | 12 |  | 2-4 | 2 | 4 | USA |  | *(Molitoris et al., 2006)* |
| *Bacteroides vulgatus* | 43 | ADM | ≤0.5-16 | 2 | 4 | USA | 2010-2012 | *(Snydman et al., 2017)* |
|  | 15 | ADM | 1-2 | 2 | 2 | USA | 2015-2017 | *(Goldstein et al., 2020)* |
|  | 11 |  | 1-8 | 2 | 4 | USA |  | *(Molitoris et al., 2006)* |
|  | 6 |  | 2-4 | 4 |  |  |  | *(Goldstein et al., 2017)* |
| *Bacteroides caccae* | 14 | ADM | 1-4 | 2 | 4 | USA | 2010-2012 | *(Snydman et al., 2017)* |
|  | 7 |  | 2-8 | 4 |  |  |  | *(Goldstein et al., 2017)* |
| *Bacteroides xylanisolvens* | 7 |  | 2-4 | 4 |  |  |  | *(Goldstein et al., 2017)* |
| *Bacteroides, Porphyromonas,Preiatella* | 50 | ADM | 0.25-8 | 2 | 4 | Swedenwere |  | *(Edlund et al., 1999)* |
| *Prevotella spp.* | 44 | ADM | ≤0.06-8 | 2 | 4 | France | 1999-2000 | *(Behra-Miellet et al., 2003)* |
|  | 12 | ADM | 0.25-2 | 0.5 | 1 |  | 2000-2003 | *(Citron et al., 2003)* |
|  | 10 | ADM | 1-8 | 4 | 8 | India |  | *(Mathur et al., 2011)* |
|  | 28 |  | 0.25-4 | 1 | 4 | USA |  | *(Molitoris et al., 2006)* |
|  | 15 |  | 1-2 | 2 | 2 | USA | 1990-1997 | *(Goldstein et al., 1999)* |
| *Prevotella bivia* | 17 |  | 1-4 | 2 | 4 | Kuwait |  | *(Phillips et al., 2003)* |
|  | 10 | ADM | 2-8 | 4 | 4 | USA | 2015-2017 | *(Goldstein et al., 2020)* |
| *Prevotella buccae* | 10 | ADM | 2-2 | 2 | 2 | USA | 2015-2017 | *(Goldstein et al., 2020)* |
| *Prevotella heparinolytica* | 16 | ADM | 0.25-2 | 2 | 2 | U.S. European and Canadian |  | *(Goldstein et al., 2017)* |
|  | 13 |  | 2 | 2 | 2 | USA | 1990-1997 | *(Goldstein et al., 1999)* |
| *Prevotella melaninogenica* | 10 | ADM | 2-4 | 2 | 4 | USA | 2015-2017 | *(Goldstein et al., 2020)* |
| *Prevotella oralis* | 10 | ADM | 0.5-4 | 2 | 2 | USA | 2015-2017 | *(Goldstein et al., 2020)* |
| *Prevotella spp. and other Gram-negative bacilli* | 52 | E-test | 0.016-＞256 | 1 | 2 | Belgian | 2011-2012 | *(Wybo et al., 2014)* |
| *Prevotella/Porphyromonasspp.* | 50 | ADM | 0.5-4 | 1 | 2 | USA | 1999-2002 | *(Ednie et al., 2002)* |
| *Porphyromonas spp.* | 6 | ADM | ≤0.06-1 |  |  | France | 1999-2000 | *(Behra-Miellet et al., 2003)* |
|  | 10 | ADM | 0.5-0.4 | 1 | 2 | USA | 2015-2017 | *(Goldstein et al., 2020)* |
|  | 25 |  | 0.25-2 | 1 | 2 | USA | 1990-1997 | *(Goldstein et al., 1999)* |
|  | 23 |  | 0.5-2 | 1 | 2 | USA |  | *(Molitoris et al., 2006)* |
| *Porphyromonas asaccharolytica* | 10 | ADM | 2-2 | 2 | 2 |  | 2000-2003 | *(Citron et al., 2003)* |
| *Fusobacterium spp.* | 21 | ADM | ≤0.06-2 | 0.5 | 1 | France | 1999-2000 | *(Behra-Miellet et al., 2003)* |
|  | 21 | E-test | 0.064-8 | 0.25 | 1 | Belgian | 2011-2012 | *(Wybo et al., 2014)* |
|  | 10 | ADM | 0.25-0.5 | 0.5 | 0.5 | USA | 2015-2017 | *(Goldstein et al., 2020)* |
|  | 24 | ADM | 0.25-2 | 0.5 | 2 | USA | 1999-2002 | *(Ednie et al., 2002)* |
|  | 6 |  | 2 | 2 | 2 | Kuwait |  | *(Phillips et al., 2003)* |
|  | 10 |  | 0.125-1 | 0.5 | 0.5 | USA | 1990-1997 | *(Goldstein et al., 1999)* |
|  | 35 |  | 0.25-2 | 0.5 | 1 | USA |  | *(Molitoris et al., 2006)* |
| *Fusobacterium necrophorum* | 34 | E-test | 0.047-0.25 | 0.125 | 0.25 | Germany |  | *(Daeschlein et al., 2006)* |
|  | 10 | ADM | 0.5-1 | 0.5 | 1 | USA | 2015-2017 | *(Goldstein et al., 2020)* |
| *Fusobacterium nucleatum* | 18 |  | ≤0.03-1 | 0.5 | 1 | USA | 1990-1997 | *(Goldstein et al., 1999)* |
|  | 20 | E-test | 0.064-0.38 | 0.19 | 0.25 | Germany |  | *(Daeschlein et al., 2006)* |
| *Fusobacterium* | 30 | ADM | 0.25-8 | 0.5 | 8 | Swedenwere |  | *(Edlund et al., 1999)* |
| *Fusobacterium canifelinum* | 10 | ADM | 0.5-1 | 1 | 1 |  |  | *(Goldstein et al., 2017)* |
| *Fusobacterium. russii* | 10 | ADM | 1-1 | 1 | 1 |  |  | *(Goldstein et al., 2017)* |
| *Fusobacterium varium* | 18 | E-test | 0.125-1.0 | 0.5 | 0.75 | Germany |  | *(Daeschlein et al., 2006)* |
|  | 10 | ADM | 0.25-2 | 0.5 | 0.5 | USA | 2015-2017 | *(Goldstein et al., 2020)* |
| *Fusobacterium. mortiferum* | 8 | E-test | 0.016-0.25 | 0.094 | 0.19 | Germany |  | *(Daeschlein et al., 2006)* |
| *Fusobacterium-Veillonella spp.* | 15 | ADM | 0.25-2 | 0.5 | 1 |  | 2000-2003 | *(Citron et al., 2003)* |
| *Veillonella spp.* | 5 | ADM | ≤0.06-0.5 |  |  | France | 1999-2000 | *(Behra-Miellet et al., 2003)* |
|  | 10 | ADM | 2-8 | 2 | 4 | USA | 2015-2017 | *(Goldstein et al., 2020)* |
| *Pasteurella species* | 17 |  | 1-＞32 | 2 | 32 | USA | 1990-1997 | *(Goldstein et al., 1999)* |
| *Pasteurella dagmatis* | 11 |  | 0.5-4 | 2 | 4 | USA | 1990-1997 | *(Goldstein et al., 1999)* |
| *Pasteurella canis* | 21 |  | 1-8 | 2 | 2 | USA | 1990-1997 | *(Goldstein et al., 1999)* |
| *Pasteurella haemolytica* | 7 |  | ＞32 | ＞32 |  | USA | 1990-1997 | *(Goldstein et al., 1999)* |
| *Pasteurella multocida subsp. multocida* | 30 |  | 1-2 | 2 | 2 | USA | 1990-1997 | *(Goldstein et al., 1999)* |
| *Pasteurella multocida subsp. septica* | 43 |  | 1-2 | 2 | 2 | USA | 1990-1997 | *(Goldstein et al., 1999)* |
| *Pasteurella stomatis* | 19 |  | 1-16 | 4 | 8 | USA | 1990-1997 | *(Goldstein et al., 1999)* |
| *Anaerococcus spp.* | 10 | ADM | 0.5-2 | 1 | 1 | French | 2016-2020 | *(Guérin et al., 2021)* |
| *Anaerococcus prevotii* | 11 | ADM | ≤0.03-0.12 | ≤0.03 | 0.06 |  | 1996-2002 | *(Goldstein et al., 2005)* |
|  | 11 | ADM | ≤0.03-2 | 0.5 | 1 | USA | 1996-2002 | *(Goldstein et al., 2004)* |
|  | 20 | ADM | 0.5-2 | 1 | 1 | USA |  | *(Goldstein et al., 2006)* |
| *Campylobacter gracilis* | 11 |  | 4-64 | 16 | 32 | USA |  | *(Molitoris et al., 2006)* |
| *Parabacteroides distasonis* | 34 | ADM | 1-4 | 2 | 4 | USA | 2010-2012 | *(Snydman et al., 2017)* |
|  | 11 | ADM | 2-4 | 4 | 4 | USA | 2015-2017 | *(Goldstein et al., 2020)* |
| *Parabacteroides goldsteinii* | 10 | ADM | 1-4 | 4 | 4 | USA | 2015-2017 | *(Goldstein et al., 2020)* |
| *Parabacteroides merdae* | 10 | ADM | 1-4 | 4 | 4 | USA | 2015-2017 | *(Goldstein et al., 2020)* |
| *Weeksella zoohelcum* | 10 |  | 0.25-2 | 1 | 2 | USA | 1990-1997 | *(Goldstein et al., 1999)* |
| *Moraxella catarrhalis* | 27 | ADM | 2-8 | 4 | 4 | Korea | 2002-2004 | *(Yum et al., 2010)* |
| *Moraxella species* | 12 |  | 4-16 | 4 | 8 | USA | 1990-1997 | *(Goldstein et al., 1999)* |
| *Eikenella corrodens* | 20 |  | 4-16 | 8 | 16 | USA | 1990-1997 | *(Goldstein et al., 1999)* |
| *Bactrroides jagilis* | 100 | ADM | 2-4 | 4 | 4 | Swedenwere |  | *(Edlund et al., 1999)* |
| *Bilophila wadsworthia* | 11 | ADM | 4-16 | 16 | 16 | USA | 2015-2017 | *(Goldstein et al., 2020)* |
|  | 16 |  | 8-32 | 16 | 32 | USA |  | *(Molitoris et al., 2006)* |
| *Miscellaneous clostridia* | 43 | ADM | 0.5-8 | 2 |  | USA | 1999-2002 | *(Ednie et al., 2002)* |
| *Actinobacillus-Haemophilus species* | 9 |  | ≤0.03-＞32 | 4 |  | USA | 1990-1997 | *(Goldstein et al., 1999)* |
| *Sutterella wadsworthensis* | 11 |  | 32-＞128 | 128 | ＞128 | USA |  | *(Molitoris et al., 2006)* |
| *Other anaerobic gram-positive cocci* | 31 | ADM | 0.5-2 | 1 | 1 | USA |  | *(Goldstein et al., 2006)* |
| **Others** *Listeria monocytogenes* | 6 | BMD | 0.5-2 | 1 | 2 | China |  | *(Yu et al., 2021)* |
|  | 60 | E-test | 0.75-1.5 |  | 1 | Germany |  | *(Callapina et al., 2001)* |
| *Listeria spp.* | 27 | BMD | 2 | 2 | 2 | USA |  | *(Jones et al., 2002)* |
|  | 24 | BMD |  | 1 | 2 | whole world | 2011-2013 | *(Mendes et al., 2015)* |
| *Rhodococcus equi* | 103 | BMD | 0.5-2 | 2 | 2 |  |  | *(Bowersock et al., 2000)* |
|  | 12 | BMD | 0.5-2 | 0.5 |  | Italy | 1990-2003 | *(Giacometti et al., 2005)* |
|  | 70 | BMD | ≤1 | ≤1 | ≤1 | USA | 1989-2019 | *(Erol et al., 2021)* |
| *Micrococcus spp.* | 11 | BMD |  | 0.5 | 0.5 | whole world | 2011-2013 | *(Mendes et al., 2015)* |
|  | 11 | BMD | 1 | 1 | 0.5-1 | USA |  | *(Jones et al., 2002)* |
| *Stomatococcus spp.* | 6 | BMD | 1 |  | 0.5-1 | USA |  | *(Jones et al., 2002)* |
| *Bacillus spp.* | 21 | BMD | 0.25-2 | 1 | 2 | USA | 2014-2016 | *(Rolston et al., 2018)* |
|  | 23 | BMD | 1 | 1 | 0.5-1 | USA |  | *(Jones et al., 2002)* |
| *Bacillus anthracis* | 110 | BMD | 0.06-64 | 1 | 2 | Italy | 1984-2017 | *(Manzulli et al., 2019)* |
| *Turicella otitidis* | 20 | ADM | ≤0.12-0.5 | 0.5 | 0.5 |  | 2001-2004 | *(Gómez-Garcés et al., 2007)* |
| *Haemophilus influenzae* | 24 |  | 2-8 | 8 | 8 | Korea | 2000-2001 | *(Yong et al., 2004)* |
|  | 25 | ADM | 4-16 | 8 | 16 | Korea | 2002-2004 | *(Yum et al., 2010)* |
| *Moraxella catarrhalis* | 24 |  | 2-32 | 4 | 4 | Korea | 2000-2001 | *(Yong et al., 2004)* |
|  | 11 |  | 4-32 | 8 | 8 | USA | 1990-1997 | *(Goldstein et al., 1999)* |
| *Neisseria weaveri* | 13 |  | 4-16 | 8 | 16 | USA | 1990-1997 | *(Goldstein et al., 1999)* |
| *Neisseria species* | 10 |  | 8-32 | 16 | 16 | USA | 1990-1997 | *(Goldstein et al., 1999)* |

Abbreviations: N, number of strains; MIC, minimum inhibitory concentration; BMD; broth microdilution; ADM, agar dilution method.

**REFERENCES**

**Abe M, Kimura M, Maruyama H, Watari T, Ogura S, Takagi S, Uchida N, Otsuka Y, Taniguchi S, Araoka H**. **2021**. Clinical characteristics and drug susceptibility patterns of Corynebacterium species in bacteremic patients with hematological disorders. *European journal of clinical microbiology & infectious diseases : official publication of the European Society of Clinical Microbiology* **40(10)**:2095-2104 DOI 10.1007/s10096-021-04257-8.

**Ahmed I, Jabeen K, Inayat R, Hasan R**. **2013**. Susceptibility testing of extensively drug-resistant and pre-extensively drug-resistant Mycobacterium tuberculosis against levofloxacin, linezolid, and amoxicillin-clavulanate. *Antimicrobial agents and chemotherapy* **57(6)**:2522-2525 DOI 10.1128/AAC.02020-12.

**Alcalá L, Ruiz-Serrano MJ, Pérez-Fernández Turégano C, García De Viedma D, Díaz-Infantes M, Marín-Arriaza M, Bouza E**. **2003**. In vitro activities of linezolid against clinical isolates of Mycobacterium tuberculosis that are susceptible or resistant to first-line antituberculous drugs. *Antimicrobial agents and chemotherapy* **47(1)**:416-417 DOI 10.1128/AAC.47.1.416-417.2003.

**An H, Sun W, Liu X, Wang T, Qiao J, Liang J**. **2023**. In vitro activities of contezolid (MRX-I) against drug-sensitive and drug-resistant Mycobacterium tuberculosis. *Microbiology spectrum* **11(5)**:e0462722 DOI 10.1128/spectrum.04627-22.

**Aono A, Murase Y, Chikamatsu K, Igarashi Y, Shimomura Y, Hosoya M, Osugi A, Morishige Y, Takaki A, Yamada H, Mitarai S**. **2022**. In vitro activity of tedizolid and linezolid against multidrug-resistant Mycobacterium tuberculosis: a comparative study using microdilution broth assay and genomics. *Diagnostic microbiology and infectious disease* **103(3)**:115714 DOI 10.1016/j.diagmicrobio.2022.115714.

**Araj GF, Baba OZ, Itani LY, Avedissian AZ, Sobh GM**. **2019**. Non-tuberculous mycobacteria profiles and their anti-mycobacterial resistance at a major medical center in Lebanon. *Journal of infection in developing countries* **13(7)**:612-618 DOI 10.3855/jidc.11028.

**Barberis CM, Sandoval E, Rodriguez CH, Ramírez MS, Famiglietti A, Almuzara M, Vay C**. **2018**. Comparison between disk diffusion and agar dilution methods to determine in vitro susceptibility of Corynebacterium spp. clinical isolates and update of their susceptibility. *Journal of global antimicrobial resistance* **14**:246-252 DOI 10.1016/j.jgar.2018.05.009.

**Behra-Miellet J, Calvet L, Dubreuil L**. **2003**. Activity of linezolid against anaerobic bacteria. *International journal of antimicrobial agents* **22(1)**:28-34 DOI 10.1016/s0924-8579(03)00087-6.

**Bowersock TL, Salmon SA, Portis ES, Prescott JF, Robison DA, Ford CW, Watts JL**. **2000**. MICs of oxazolidinones for Rhodococcus equi strains isolated from humans and animals. *Antimicrobial agents and chemotherapy* **44(5)**:1367-1369 DOI 10.1128/AAC.44.5.1367-1369.2000.

**Brown-Elliott BA, Crist CJ, Mann LB, Wilson RW, Wallace RJ Jr**. **2003**. In vitro activity of linezolid against slowly growing nontuberculous Mycobacteria. *Antimicrobial agents and chemotherapy* **47(5)**:1736-1738 DOI 10.1128/AAC.47.5.1736-1738.2003.

**Brown-Elliott BA, Rubio A, Wallace RJ Jr**. **2018**. In Vitro Susceptibility Testing of a Novel Benzimidazole, SPR719, against Nontuberculous Mycobacteria. *Antimicrobial agents and chemotherapy* **62(11)**:e01503-01518 DOI 10.1128/AAC.01503-18.

**Brown-Elliott BA, Wallace RJ Jr**. **2017**. In Vitro Susceptibility Testing of Tedizolid against Isolates of Nocardia. *Antimicrobial agents and chemotherapy* **61(12)**:e01537-01517 DOI 10.1128/AAC.01537-17.

**Brown-Elliott BA, Wallace RJ Jr**. **2017**. In Vitro Susceptibility Testing of Tedizolid against Nontuberculous Mycobacteria. *Journal of clinical microbiology* **55(6)**:1747-1754 DOI 10.1128/JCM.00274-17.

**Callapina M, Kretschmar M, Dietz A, Mosbach C, Hof H, Nichterlein T**. **2001**. Systemic and intracerebral infections of mice with Listeria monocytogenes successfully treated with linezolid. *Journal of chemotherapy* **13(3)**:265-269 DOI 10.1179/joc.2001.13.3.265.

**Cavanaugh JS, Jou R, Wu MH, Dalton T, Kurbatova E, Ershova J, Cegielski JP**. **2017**. Susceptibilities of MDR Mycobacterium tuberculosis isolates to unconventional drugs compared with their reported pharmacokinetic/pharmacodynamic parameters. *The Journal of antimicrobial chemotherapy* **72(6)**:1678-1687 DOI 10.1093/jac/dkx022.

**Chapartegui-González I, Fernández-Martínez M, Rodríguez-Fernández A, Rocha D, Aguiar E, Pacheco L, Ramos-Vivas J, Calvo J, Martínez-Martínez L, Navas J**. **2020**. Antimicrobial Susceptibility and Characterization of Resistance Mechanisms of Corynebacterium urealyticum Clinical Isolates. *Antibiotics (Basel, Switzerland)* **9(7)**:404 DOI 10.3390/antibiotics9070404.

**Cho EH, Huh HJ, Song DJ, Moon SM, Lee SH, Shin SY, Kim CK, Ki CS, Koh WJ, Lee NY**. **2018**. Differences in drug susceptibility pattern between Mycobacterium avium and Mycobacterium intracellulare isolated in respiratory specimens. *Journal of infection and chemotherapy : official journal of the Japan Society of Chemotherapy* **24(4)**:315-318 DOI 10.1016/j.jiac.2017.10.022.

**Citron DM, Merriam CV, Tyrrell KL, Warren YA, Fernandez H, Goldstein EJ**. **2003**. In vitro activities of ramoplanin, teicoplanin, vancomycin, linezolid, bacitracin, and four other antimicrobials against intestinal anaerobic bacteria. *Antimicrobial agents and chemotherapy* **47(7)**:2334-2338 DOI 10.1128/AAC.47.7.2334-2338.2003.

**Daeschlein G, Hoehne C, Assadian O, Daxboeck F, Meinl C, Kramer A, Kekulé AS**. **2006**. In vitro activity of linezolid against clinical isolates of Fusobacterium spp. *The Journal of antimicrobial chemotherapy* **58(4)**:789-793 DOI 10.1093/jac/dkl338.

**Daneshfar S, Khosravi AD, Hashemzadeh M**. **2022**. Drug susceptibility profiling and genetic determinants of drug resistance in Mycobacterium simiae isolates obtained from regional tuberculosis reference laboratories of Iran. *PloS one* **17(8)**:e0267320 DOI 10.1371/journal.pone.0267320.

**Davidson N, Grigg MJ, Mcguinness SL, Baird RJ, Anstey NM**. **2020**. Safety and Outcomes of Linezolid Use for Nocardiosis. *Open forum infectious diseases* **7(4)**:ofaa090 DOI 10.1093/ofid/ofaa090.

**Edlund C, Oh H, Nord CE**. **1999**. In vitro activity of linezolid and eperezolid against anaerobic bacteria. *Clinical microbiology and infection : the official publication of the European Society of Clinical Microbiology and Infectious Diseases* **5(1)**:51-53 DOI 10.1111/j.1469-0691.1999.tb00099.x.

**Ednie LM, Jacobs MR, Appelbaum PC**. **2002**. Anti-anaerobic activity of AZD2563, a new oxazolidinone, compared with eight other agents. *The Journal of antimicrobial chemotherapy* **50(1)**:101-105 DOI 10.1093/jac/dkf088.

**Ermertcan S, Hosgor-Limoncu M, Erac B, Tasli H, Cavusoglu C, Bozkurt H**. **2009**. In vitro activity of linezolid against Mycobacterium tuberculosis strains isoalted from Western Turkey. *Japanese journal of infectious diseases* **62(5)**:384-385

**Erol E, Scortti M, Fortner J, Patel M, Vázquez-Boland JA**. **2021**. Antimicrobial Resistance Spectrum Conferred by pRErm46 of Emerging Macrolide (Multidrug)-Resistant Rhodococcus equi. *Journal of clinical microbiology* **59(10)**:e0114921 DOI 10.1128/JCM.01149-21.

**Erturan Z, Uzun M**. **2005**. In vitro activity of linezolid against multidrug-resistant Mycobacterium tuberculosis isolates. *International journal of antimicrobial agents* **26(1)**:78-80 DOI 10.1016/j.ijantimicag.2005.03.006.

**Fernandez-Roblas R, Adames H, Martín-de-Hijas NZ, Almeida DG, Gadea I, Esteban J**. **2009**. In vitro activity of tigecycline and 10 other antimicrobials against clinical isolates of the genus Corynebacterium. *International journal of antimicrobial agents* **33(5)**:453-455 DOI 10.1016/j.ijantimicag.2008.11.001.

**Galar A, Martín-Rabadán P, Marín M, Cercenado E, Sánchez-Carrillo C, Valerio M, Bouza E, Muñoz P**. **2021**. Revisiting nocardiosis at a tertiary care institution: Any change in recent years. *International journal of infectious diseases : IJID : official publication of the International Society for Infectious Diseases* **102**:446-454 DOI 10.1016/j.ijid.2020.10.087.

**Gao T, Yao C, Shang Y, Su R, Zhang X, Ren W, Li S, Shu W, Pang Y, Li Q**. **2023**. Antimicrobial Effect of Oxazolidinones and Its Synergistic Effect with Bedaquiline Against Mycobacterium abscessus Complex. *Infection and drug resistance* **16**:279-287 DOI 10.2147/IDR.S395750.

**Giacometti A, Cirioni O, Kamysz W, Silvestri C, Del Prete MS, Licci A, D'Amato G, Lukasiak J, Scalise G**. **2005**. In vitro activity of citropin 1.1 alone and in combination with clinically used antimicrobial agents against Rhodococcus equi. *The Journal of antimicrobial chemotherapy* **56(2)**:410-412 DOI 10.1093/jac/dki236.

**Gitti Z, Mantadakis E, Maraki S, Samonis G**. **2011**. Clinical significance and antibiotic susceptibilities of nontuberculous mycobacteria from patients in Crete, Greece. *Future microbiology* **6(9)**:1099-1109 DOI 10.2217/fmb.11.91.

**Go JR, Wengenack NL, Abu Saleh OM, Corsini Campioli C, Deml SM, Wilson JW**. **2020**. Mycobacterium septicum: a 6-Year Clinical Experience from a Tertiary Hospital and Reference Laboratory. *Journal of clinical microbiology* **58(12)**:e02091-02020 DOI 10.1128/JCM.02091-20.

**Goldstein E, Citron DM, Tyrrell KL, Leoncio ES**. **2017**. In Vitro Activity of Pexiganan and 10 Comparator Antimicrobials against 234 Isolates, Including 93 Pasteurella Species and 50 Anaerobic Bacterial Isolates Recovered from Animal Bite Wounds. *Antimicrobial agents and chemotherapy* **61(6)**:e00246-00217 DOI 10.1128/AAC.00246-17.

**Goldstein E, Merriam CV, Citron DM**. **2020**. In Vitro Activity of Tedizolid Compared to Linezolid and Five Other Antimicrobial Agents against 332 Anaerobic Isolates, Including Bacteroides fragilis Group, Prevotella, Porphyromonas, and Veillonella Species. *Antimicrobial agents and chemotherapy* **64(9)**:e01088-01020 DOI 10.1128/AAC.01088-20.

**Goldstein EJ, Citron DM, Merriam CV**. **1999**. Linezolid activity compared to those of selected macrolides and other agents against aerobic and anaerobic pathogens isolated from soft tissue bite infections in humans. *Antimicrobial agents and chemotherapy* **43(6)**:1469-1474 DOI 10.1128/AAC.43.6.1469.

**Goldstein EJ, Citron DM, Merriam CV, Warren YA, Tyrrell KL, Fernandez HT**. **2004**. In vitro activities of the new semisynthetic glycopeptide telavancin (TD-6424), vancomycin, daptomycin, linezolid, and four comparator agents against anaerobic gram-positive species and Corynebacterium spp. *Antimicrobial agents and chemotherapy* **48(6)**:2149-2152 DOI 10.1128/AAC.48.6.2149-2152.2004.

**Goldstein EJ, Citron DM, Merriam CV, Warren YA, Tyrrell KL, Fernandez HT, Bryskier A**. **2005**. Comparative in vitro activities of XRP 2868, pristinamycin, quinupristin-dalfopristin, vancomycin, daptomycin, linezolid, clarithromycin, telithromycin, clindamycin, and ampicillin against anaerobic gram-positive species, actinomycetes, and lactobacilli. *Antimicrobial agents and chemotherapy* **49(1)**:408-413 DOI 10.1128/AAC.49.1.408-413.2005.

**Goldstein EJ, Citron DM, Tyrrell KL, Leoncio ES, Merriam CV**. **2017**. The underappreciated in vitro activity of tedizolid against Bacteroides fragilis species, including strains resistant to metronidazole and carbapenems. *Anaerobe* **43**:1-3 DOI 10.1016/j.anaerobe.2016.09.008.

**Goldstein EJ, Citron DM, Warren YA, Tyrrell KL, Merriam CV, Fernandez HT**. **2006**. In vitro activities of dalbavancin and 12 other agents against 329 aerobic and anaerobic gram-positive isolates recovered from diabetic foot infections. *Antimicrobial agents and chemotherapy* **50(8)**:2875-2879 DOI 10.1128/AAC.00286-06.

**Goodlet KJ, Tokman S, Nasar A, Cherrier L, Walia R, Nailor MD**. **2021**. Nocardia prophylaxis, treatment, and outcomes of infection in lung transplant recipients: A matched case-control study. *Transplant Infectious Disease* **23(2)**:e13478 DOI 10.1111/tid.13478.

**Guo Q, Xu L, Tan F, Zhang Y, Fan J, Wang X, Zhang Z, Li B, Chu H**. **2021**. A Novel Oxazolidinone, Contezolid (MRX-I), Expresses Anti-Mycobacterium abscessus Activity In Vitro. *Antimicrobial agents and chemotherapy* **65(11)**:e0088921 DOI 10.1128/AAC.00889-21.

**Guo S, Wang B, Fu L, Chen X, Zhang W, Huang H, Lu Y**. **2021**. In Vitro and In Vivo Activity of Oxazolidinone Candidate OTB-658 against Mycobacterium tuberculosis. *Antimicrobial agents and chemotherapy* **65(11)**:e0097421 DOI 10.1128/AAC.00974-21.

**Guo Y, Yang J, Wang W, Wu X, Wan B, Wang H, Sha W, Yu F**. **2023**. Bedaquiline, Delamanid, Linezolid, Clofazimine, and Capreomycin MIC Distributions for Drug Resistance Mycobacterium tuberculosis in Shanghai, China. *Infection and drug resistance* **16**:7587-7595 DOI 10.2147/IDR.S440711.

**Guérin F, Dejoies L, Degand N, Guet-Revillet H, Janvier F, Corvec S, Barraud O, Guillard T, Walewski V, Gallois E, Cattoir V, Group OBOTGS**. **2021**. In Vitro Antimicrobial Susceptibility Profiles of Gram-Positive Anaerobic Cocci Responsible for Human Invasive Infections. *Microorganisms* **9(8)**:1665 DOI 10.3390/microorganisms9081665.

**Gómez-Garcés JL, Alos JI, Tamayo J**. **2007**. In vitro activity of linezolid and 12 other antimicrobials against coryneform bacteria. *International journal of antimicrobial agents* **29(6)**:688-692 DOI 10.1016/j.ijantimicag.2006.11.032.

**Harris DM, Dumitrascu AG, Chirila RM, Omer M, Stancampiano FF, Hata DJ, Meza Villegas DM, Heckman MG, Cochuyt JJ, Alvarez S**. **2021**. Invasive Nocardiosis in Transplant and Nontransplant Patients: 20-Year Experience in a Tertiary Care Center. *Mayo Clinic proceedings. Innovations, quality & outcomes* **5(2)**:298-307 DOI 10.1016/j.mayocpiqo.2020.10.009.

**He G, Wu L, Zheng Q, Jiang X**. **2022**. Antimicrobial susceptibility and minimum inhibitory concentration distribution of common clinically relevant non-tuberculous mycobacterial isolates from the respiratory tract. *Annals of medicine* **54(1)**:2500-2510 DOI 10.1080/07853890.2022.2121984.

**Heidarieh P, Mirsaeidi M, Hashemzadeh M, Feizabadi MM, Bostanabad SZ, Nobar MG, Hashemi Shahraki A**. **2016**. In Vitro Antimicrobial Susceptibility of Nontuberculous Mycobacteria in Iran. *Microbial drug resistance (Larchmont, N.Y.)* **22(2)**:172-178 DOI 10.1089/mdr.2015.0134.

**Huang CC, Wu MF, Chen HC, Huang WC**. **2018**. In vitro activity of aminoglycosides, clofazimine, d-cycloserine and dapsone against 83 Mycobacterium avium complex clinical isolates. *Journal of microbiology, immunology, and infection = Wei mian yu gan ran za zhi* **51(5)**:636-643 DOI 10.1016/j.jmii.2017.05.001.

**Huang TS, Liu YC, Sy CL, Chen YS, Tu HZ, Chen BC**. **2008**. In vitro activities of linezolid against clinical isolates of Mycobacterium tuberculosis complex isolated in Taiwan over 10 years. *Antimicrobial agents and chemotherapy* **52(6)**:2226-2227 DOI 10.1128/AAC.00414-07.

**Hunkins JJ, de-Moura VC, Eddy JJ, Daley CL, Khare R**. **2023**. In vitro susceptibility patterns for rapidly growing nontuberculous mycobacteria in the United States. *Diagnostic microbiology and infectious disease* **105(3)**:115882 DOI 10.1016/j.diagmicrobio.2022.115882.

**Johnson AP, Warner M, Malnick H, Livermore DM**. **2003**. Activity of the oxazolidinones AZD2563 and linezolid against Corynebacterium jeikeium and other Corynebacterium spp. *The Journal of antimicrobial chemotherapy* **51(3)**:745-747 DOI 10.1093/jac/dkg129.

**Jones RN, Biedenbach DJ, Anderegg TR**. **2002**. In vitro evaluation of AZD2563, a new oxazolidinone, tested against unusual gram-positive species. *Diagnostic microbiology and infectious disease* **42(2)**:119-122 DOI 10.1016/s0732-8893(01)00329-7.

**Kardan-Yamchi J, Kazemian H, Battaglia S, Abtahi H, Foroushani AR, Hamzelou G, Cirillo DM, Ghodousi A, Feizabadi MM**. **2020**. Whole Genome Sequencing Results Associated with Minimum Inhibitory Concentrations of 14 Anti-Tuberculosis Drugs among Rifampicin-Resistant Isolates of Mycobacterium Tuberculosis from Iran. *Journal of clinical medicine* **9(2)**:465 DOI 10.3390/jcm9020465.

**Kazemian H, Haeili M, Kardan Yamchi J, Rezaei F, Gizaw Feyisa S, Zahednamazi F, Mohajeri P, Zaker Bostanabd S, Hashemi Shahraki A, Imani Fooladi AA, Feizabadi MM**. **2015**. Antimycobacterial activity of linezolid against multidrug-resistant and extensively drug-resistant strains of Mycobacterium tuberculosis in Iran. *International journal of antimicrobial agents* **45(6)**:668-670 DOI 10.1016/j.ijantimicag.2015.02.004.

**Kim DH, Kim SY, Koh WJ, Jhun BW**. **2021**. In Vitro Activity of Oxazolidinone against Nontuberculous Mycobacteria, Including Macrolide-Resistant Clinical Isolates. *Antimicrobial agents and chemotherapy* **65(7)**:e0230620 DOI 10.1128/AAC.02306-20.

**Kuo SF, Chen FJ, Lan IC, Chien CC, Lee CH**. **2022**. Epidemiology of Nocardia Species at a Tertiary Hospital in Southern Taiwan, 2012 to 2020: MLSA Phylogeny and Antimicrobial Susceptibility. *Antibiotics (Basel, Switzerland)* **11(10)**:1438 DOI 10.3390/antibiotics11101438.

**Lao CK, Tseng MC, Chiu CH, Chen NY, Chen CH, Chung WH, Liu TP, Lu JJ, Lai HC, Yang LY, Lee CH, Wu TS**. **2022**. Clinical manifestations and antimicrobial susceptibility of Nocardia species at a tertiary hospital in Taiwan, 2011-2020. *Journal of the Formosan Medical Association = Taiwan yi zhi* **121(10)**:2109-2122 DOI 10.1016/j.jfma.2022.06.011.

**Lebeaux D, Bergeron E, Berthet J, Djadi-Prat J, Mouniée D, Boiron P, Lortholary O, Rodriguez-Nava V**. **2019**. Antibiotic susceptibility testing and species identification of Nocardia isolates: a retrospective analysis of data from a French expert laboratory, 2010-2015. *Clinical microbiology and infection : the official publication of the European Society of Clinical Microbiology and Infectious Diseases* **25(4)**:489-495 DOI 10.1016/j.cmi.2018.06.013.

**Lee EH, Chin B, Kim YK, Yoo JS, Choi YH, Kim S, Lee KH, Lee SJ, Kim J, Baek YJ, Kim JH, Ahn JY, Jeong SJ, Ku NS, Yeom JS, Choi JY**. **2022**. Clinical characteristics of nontuberculous mycobacterial disease in people living with HIV/AIDS in South Korea: A multi-center, retrospective study. *PloS one* **17(11)**:e0276484 DOI 10.1371/journal.pone.0276484.

**Lee MC, Sun PL, Wu TL, Wang LH, Yang CH, Chung WH, Kuo AJ, Liu TP, Lu JJ, Chiu CH, Lai HC, Chen NY, Yang JH, Wu TS**. **2017**. Antimicrobial resistance in Mycobacterium abscessus complex isolated from patients with skin and soft tissue infections at a tertiary teaching hospital in Taiwan. *The Journal of antimicrobial chemotherapy* **72(10)**:2782-2786 DOI 10.1093/jac/dkx212.

**Lee Y, Hong SK, Choi S, Im W, Yong D, Lee K**. **2015**. In vitro activity of tedizolid against gram-positive bacteria in patients with skin and skin structure infections and hospital-acquired pneumonia: a Korean multicenter study. *Annals of laboratory medicine* **35(5)**:523-530 DOI 10.3343/alm.2015.35.5.523.

**Lefèvre CR, Pelletier R, Le Monnier A, Corvec S, Bille E, Potron A, Fihman V, Farfour E, Amara M, Degand N, Barraud O, Cattoir V, Group FTGS**. **2021**. Clinical relevance and antimicrobial susceptibility profile of the unknown human pathogen Corynebacterium aurimucosum. *Journal of medical microbiology* **70(3)**DOI 10.1099/jmm.0.001334.

**Li G, Pang H, Guo Q, Huang M, Tan Y, Li C, Wei J, Xia Y, Jiang Y, Zhao X, Liu H, Zhao LL, Liu Z, Xu D, Wan K**. **2017**. Antimicrobial susceptibility and MIC distribution of 41 drugs against clinical isolates from China and reference strains of nontuberculous mycobacteria. *International journal of antimicrobial agents* **49(3)**:364-374 DOI 10.1016/j.ijantimicag.2016.10.024.

**Li J, Shen H, Yu T, Tao XY, Hu YM, Wang HC, Zou MX**. **2022**. Isolation and Characterization of Nocardia Species from Pulmonary Nocardiosis in a Tertiary Hospital in China. *Japanese journal of infectious diseases* **75(1)**:31-35 DOI 10.7883/yoken.JJID.2020.1096.

**Litvinov V, Makarova M, Galkina K, Khachaturiants E, Krasnova M, Guntupova L, Safonova S**. **2018**. Drug susceptibility testing of slowly growing non-tuberculous mycobacteria using slomyco test-system. *PloS one* **13(9)**:e0203108 DOI 10.1371/journal.pone.0203108.

**Liu CF, Song YM, He WC, Liu DX, He P, Bao JJ, Wang XY, Li YM, Zhao YL**. **2021**. Nontuberculous mycobacteria in China: incidence and antimicrobial resistance spectrum from a nationwide survey. *Infectious diseases of poverty* **10(1)**:59 DOI 10.1186/s40249-021-00844-1.

**Lu SH, Qian ZW, Mou PP, Xie L**. **2020**. Clinical Nocardia species: Identification, clinical characteristics, and antimicrobial susceptibility in Shandong, China. *Bosnian journal of basic medical sciences* **20(4)**:531-538 DOI 10.17305/bjbms.2020.4764.

**Manzulli V, Fasanella A, Parisi A, Serrecchia L, Donatiello A, Rondinone V, Caruso M, Zange S, Tscherne A, Decaro N, Pedarra C, Galante D**. **2019**. Evaluation of in vitro antimicrobial susceptibility of Bacillus anthracis strains isolated during anthrax outbreaks in Italy from 1984 to 2017. *Journal of veterinary science* **20(1)**:58-62 DOI 10.4142/jvs.2019.20.1.58.

**Marfil E, Ruiz P, Martínez-Martínez L, Causse M**. **2022**. Comparative study of in vitro activity of tedizolid and linezolid against Mycobacterium avium complex. *Journal of global antimicrobial resistance* **30**:395-398 DOI 10.1016/j.jgar.2022.07.012.

**Mathur T, Kumar M, Barman TK, Kumar GR, Kalia V, Singhal S, Raj VS, Upadhyay DJ, Das B, Bhatnagar PK**. **2011**. Activity of RBx 11760, a novel biaryl oxazolidinone, against Clostridium difficile. *The Journal of antimicrobial chemotherapy* **66(5)**:1087-1095 DOI 10.1093/jac/dkr033.

**Mazzaferri F, Cordioli M, Segato E, Adami I, Maccacaro L, Sette P, Cazzadori A, Concia E, Azzini AM**. **2018**. Nocardia infection over 5 years (2011-2015) in an Italian tertiary care hospital. *The new microbiologica* **41(2)**:136-140

**Mendes RE, Sader HS, Flamm RK, Farrell DJ, Jones RN**. **2015**. Telavancin activity when tested by a revised susceptibility testing method against uncommonly isolated Gram-positive pathogens responsible for documented infections in hospitals worldwide (2011-2013). *Journal of global antimicrobial resistance* **3(1)**:36-39 DOI 10.1016/j.jgar.2014.12.003.

**Molitoris D, Väisänen ML, Bolaños M, Finegold SM**. **2006**. In vitro activities of DX-619 and four comparator agents against 376 anaerobic bacterial isolates. *Antimicrobial agents and chemotherapy* **50(5)**:1887-1889 DOI 10.1128/AAC.50.5.1887-1889.2006.

**Neemuchwala A, Soares D, Ravirajan V, Marchand-Austin A, Kus JV, Patel SN**. **2018**. In Vitro Antibiotic Susceptibility Pattern of Non-diphtheriae Corynebacterium Isolates in Ontario, Canada, from 2011 to 2016. *Antimicrobial agents and chemotherapy* **62(4)**:e01776-01717 DOI 10.1128/AAC.01776-17.

**Nhan TX, Parienti JJ, Badiou G, Leclercq R, Cattoir V**. **2012**. Microbiological investigation and clinical significance of Corynebacterium spp. in respiratory specimens. *Diagnostic microbiology and infectious disease* **74(3)**:236-241 DOI 10.1016/j.diagmicrobio.2012.07.001.

**Nie W, Duan H, Huang H, Lu Y, Bi D, Chu N**. **2014**. Species identification of Mycobacterium abscessus subsp. abscessus and Mycobacterium abscessus subsp. bolletii using rpoB and hsp65, and susceptibility testing to eight antibiotics. *International journal of infectious diseases : IJID : official publication of the International Society for Infectious Diseases* **25**:170-174 DOI 10.1016/j.ijid.2014.02.014.

**Oprica C, Nord CE**. **2005**. European surveillance study on the antibiotic susceptibility of Propionibacterium acnes. *Clinical microbiology and infection : the official publication of the European Society of Clinical Microbiology and Infectious Diseases* **11(3)**:204-213 DOI 10.1111/j.1469-0691.2004.01055.x.

**Pang Y, Zong Z, Huo F, Jing W, Ma Y, Dong L, Li Y, Zhao L, Fu Y, Huang H**. **2017**. In Vitro Drug Susceptibility of Bedaquiline, Delamanid, Linezolid, Clofazimine, Moxifloxacin, and Gatifloxacin against Extensively Drug-Resistant Tuberculosis in Beijing, China. *Antimicrobial agents and chemotherapy* **61(10)**:e00900-00917 DOI 10.1128/AAC.00900-17.

**Phillips OA, Rotimi VO, Jamal WY, Shahin M, Verghese TL**. **2003**. Comparative in vitro activity of PH-027 versus linezolid and other anti-anaerobic antimicrobials against clinical isolates of Clostridium difficile and other anaerobic bacteria. *Journal of chemotherapy* **15(2)**:113-117 DOI 10.1179/joc.2003.15.2.113.

**Ract P, Piau-Couapel C, Compain F, Auzou M, Michon J, Cattoir V**. **2017**. In vitro activity of tedizolid and comparator agents against Gram-positive pathogens responsible for bone and joint infections. *Journal of medical microbiology* **66(10)**:1374-1378 DOI 10.1099/jmm.0.000595.

**Rashid MU, Dalhoff A, Weintraub A, Nord CE**. **2014**. In vitro activity of MCB3681 against Clostridium difficile strains. *Anaerobe* **28**:216-219 DOI 10.1016/j.anaerobe.2014.07.001.

**Rolston K, Reitzel R, Vargas-Cruz N, Shelburne SA, Raad II, Prince RA**. **2018**. In vitro activity of tedizolid and comparator agents against clinical Gram-positive isolates recovered from patients with cancer. *Diagnostic microbiology and infectious disease* **91(4)**:351-353 DOI 10.1016/j.diagmicrobio.2018.03.009.

**Ruth MM, Koeken V, Pennings LJ, Svensson EM, Wertheim H, Hoefsloot W, van Ingen J**. **2020**. Is there a role for tedizolid in the treatment of non-tuberculous mycobacterial disease. *The Journal of antimicrobial chemotherapy* **75(3)**:609-617 DOI 10.1093/jac/dkz511.

**Senol G, Bicmen C, Gunduz A, Dereli S, Erbaycu A**. **2022**. Evaluation of antimicrobial susceptibilities of non-tuberculous mycobacteria against linezolid and tigecycline. *Indian journal of medical microbiology* **40(3)**:446-448 DOI 10.1016/j.ijmmb.2022.03.012.

**Shen Y, Wang X, Jin J, Wu J, Zhang X, Chen J, Zhang W**. **2018**. In Vitro Susceptibility of Mycobacterium abscessus and Mycobacterium fortuitum Isolates to 30 Antibiotics. *BioMed research international* **2018**:4902941 DOI 10.1155/2018/4902941.

**Shoen C, DeStefano M, Hafkin B, Cynamon M**. **2018**. In Vitro and In Vivo Activities of Contezolid (MRX-I) against Mycobacterium tuberculosis. *Antimicrobial agents and chemotherapy* **62(8)**:e00493-00418 DOI 10.1128/AAC.00493-18.

**Singh K, Sharma S, Banerjee T, Gupta A, Anupurba S**. **2022**. Mutation detection and minimum inhibitory concentration determination against linezolid and clofazimine in confirmed XDR-TB clinical isolates. *BMC microbiology* **22(1)**:236 DOI 10.1186/s12866-022-02622-x.

**Snydman DR, Jacobus NV, McDermott LA, Goldstein EJ, Harrell L, Jenkins SG, Newton D, Patel R, Hecht DW**. **2017**. Trends in antimicrobial resistance among Bacteroides species and Parabacteroides species in the United States from 2010-2012 with comparison to 2008-2009. *Anaerobe* **43**:21-26 DOI 10.1016/j.anaerobe.2016.11.003.

**Sood R, Rao M, Singhal S, Rattan A**. **2005**. Activity of RBx 7644 and RBx 8700, new investigational oxazolidinones, against Mycobacterium tuberculosis infected murine macrophages. *International journal of antimicrobial agents* **25(6)**:464-468 DOI 10.1016/j.ijantimicag.2005.01.021.

**Suh JW, Ju Y, Lee CK, Sohn JW, Kim MJ, Yoon YK**. **2019**. Molecular epidemiology and clinical significance of Corynebacterium striatum isolated from clinical specimens. *Infection and drug resistance* **12**:161-171 DOI 10.2147/IDR.S184518.

**Sun W, Ma L, Li Y, Xu Y, Wei J, Sa L, Chen X, Su J**. **2022**. In vitro Studies of Non-Diphtheriae Corynebacterium Isolates on Antimicrobial Susceptibilities, Drug Resistance Mechanisms, and Biofilm Formation Capabilities. *Infection and drug resistance* **15**:4347-4359 DOI 10.2147/IDR.S376328.

**Sánchez Hernández J, Mora Peris B, Yagüe Guirao G, Gutiérrez Zufiaurre N, Muñoz Bellido JL, Segovia Hernández M, García Rodríguez JA**. **2003**. In vitro activity of newer antibiotics against Corynebacterium jeikeium, Corynebacterium amycolatum and Corynebacterium urealyticum. *International journal of antimicrobial agents* **22(5)**:492-496 DOI 10.1016/s0924-8579(03)00121-3.

**Tang YW, Cheng B, Yeoh SF, Lin R, Teo J**. **2018**. Tedizolid Activity Against Clinical Mycobacterium abscessus Complex Isolates-An in vitro Characterization Study. *Frontiers in microbiology* **9**:2095 DOI 10.3389/fmicb.2018.02095.

**Tato M, de la Pedrosa EG, Cantón R, Gómez-García I, Fortún J, Martín-Davila P, Baquero F, Gomez-Mampaso E**. **2006**. In vitro activity of linezolid against Mycobacterium tuberculosis complex, including multidrug-resistant Mycobacterium bovis isolates. *International journal of antimicrobial agents* **28(1)**:75-78 DOI 10.1016/j.ijantimicag.2006.02.011.

**Toyokawa M, Ohana N, Ueda A, Imai M, Tanno D, Honda M, Takano Y, Ohashi K, Saito K, Shimura H**. **2021**. Identification and antimicrobial susceptibility profiles of Nocardia species clinically isolated in Japan. *Scientific reports* **11(1)**:16742 DOI 10.1038/s41598-021-95870-2.

**Vera-Cabrera L, Brown-Elliott BA, Wallace RJ Jr, Ocampo-Candiani J, Welsh O, Choi SH, Molina-Torres CA**. **2006**. In vitro activities of the novel oxazolidinones DA-7867 and DA-7157 against rapidly and slowly growing mycobacteria. *Antimicrobial agents and chemotherapy* **50(12)**:4027-4029 DOI 10.1128/AAC.00763-06.

**Vera-Cabrera L, Castro-Garza J, Rendon A, Ocampo-Candiani J, Welsh O, Choi SH, Blackwood K, Molina-Torres C**. **2005**. In vitro susceptibility of Mycobacterium tuberculosis clinical isolates to garenoxacin and DA-7867. *Antimicrobial agents and chemotherapy* **49(10)**:4351-4353 DOI 10.1128/AAC.49.10.4351-4353.2005.

**Vera-Cabrera L, Gonzalez E, Rendon A, Ocampo-Candiani J, Welsh O, Velazquez-Moreno VM, Choi SH, Molina-Torres C**. **2006**. In vitro activities of DA-7157 and DA-7218 against Mycobacterium tuberculosis and Nocardia brasiliensis. *Antimicrobial agents and chemotherapy* **50(9)**:3170-3172 DOI 10.1128/AAC.00571-06.

**Wang C, Wang G, Huo F, Xue Y, Jia J, Dong L, Zhao L, Wang F, Huang H, Duan H**. **2022**. Novel oxazolidinones harbor potent in vitro activity against the clinical isolates of multidrug-resistant Mycobacterium tuberculosis in China. *Frontiers in medicine* **9**:1067516 DOI 10.3389/fmed.2022.1067516.

**Wang H, Zhu Y, Cui Q, Wu W, Li G, Chen D, Xiang L, Qu J, Shi D, Lu B**. **2022**. Epidemiology and Antimicrobial Resistance Profiles of the Nocardia Species in China, 2009 to 2021. *Microbiology spectrum* **10(2)**:e0156021 DOI 10.1128/spectrum.01560-21.

**Wang Y, Shi X, Zhang J, Wang Y, Lv Y, Du X, ChaoLuMen Q, Wang J**. **2021**. Wide spread and diversity of mutation in the gyrA gene of quinolone-resistant Corynebacterium striatum strains isolated from three tertiary hospitals in China. *Annals of clinical microbiology and antimicrobials* **20(1)**:71 DOI 10.1186/s12941-021-00477-0.

**Wei M, Wang P, Qu J, Li R, Liu Y, Gu L, Yang C**. **2017**. Identification and antimicrobial susceptibility of clinical Nocardia species in a tertiary hospital in China. *Journal of global antimicrobial resistance* **11**:183-187 DOI 10.1016/j.jgar.2017.08.011.

**Wei M, Xu X, Yang J, Wang P, Liu Y, Wang S, Yang C, Gu L**. **2021**. MLSA phylogeny and antimicrobial susceptibility of clinical Nocardia isolates: a multicenter retrospective study in China. *BMC microbiology* **21(1)**:342 DOI 10.1186/s12866-021-02412-x.

**Wybo I, Van den Bossche D, Soetens O, Vekens E, Vandoorslaer K, Claeys G, Glupczynski Y, Ieven M, Melin P, Nonhoff C, Rodriguez-Villalobos H, Verhaegen J, Piérard D**. **2014**. Fourth Belgian multicentre survey of antibiotic susceptibility of anaerobic bacteria. *The Journal of antimicrobial chemotherapy* **69(1)**:155-161 DOI 10.1093/jac/dkt344.

**Yang C, Lei H, Wang D, Meng X, He J, Tong A, Zhu L, Jiang Y, Dong M**. **2012**. In vitro activity of linezolid against clinical isolates of Mycobacterium tuberculosis, including multidrug-resistant and extensively drug-resistant strains from Beijing, China. *Japanese journal of infectious diseases* **65(3)**:240-242 DOI 10.7883/yoken.65.240.

**Yang JS, Kim KJ, Choi H, Lee SH**. **2018**. Delamanid, Bedaquiline, and Linezolid Minimum Inhibitory Concentration Distributions and Resistance-related Gene Mutations in Multidrug-resistant and Extensively Drug-resistant Tuberculosis in Korea. *Annals of laboratory medicine* **38(6)**:563-568 DOI 10.3343/alm.2018.38.6.563.

**Yao C, Guo H, Li Q, Zhang X, Shang Y, Li T, Wang Y, Xue Z, Wang L, Li L, Pang Y**. **2021**. Prevalence of extensively drug-resistant tuberculosis in a Chinese multidrug-resistant TB cohort after redefinition. *Antimicrobial resistance and infection control* **10(1)**:126 DOI 10.1186/s13756-021-00995-8.

**Yi M, Wang L, Xu W, Sheng L, Jiang L, Yang F, Cao Q, Wu J**. **2019**. Species Distribution And Antibiotic Susceptibility Of Nocardia Isolates From Yantai, China. *Infection and drug resistance* **12**:3653-3661 DOI 10.2147/IDR.S232098.

**Yong D, Yum JH, Lee K, Chong Y, Choi SH, Rhee JK**. **2004**. In vitro activities of DA-7867, a novel oxazolidinone, against recent clinical isolates of aerobic and anaerobic bacteria. *Antimicrobial agents and chemotherapy* **48(1)**:352-357 DOI 10.1128/AAC.48.1.352-357.2004.

**Yu W, Huang Y, Ying C, Zhou Y, Zhang L, Zhang J, Chen Y, Qiu Y**. **2021**. Analysis of Genetic Diversity and Antibiotic Options for Clinical Listeria monocytogenes Infections in China. *Open forum infectious diseases* **8(6)**:ofab177 DOI 10.1093/ofid/ofab177.

**Yu X, Huo F, Wang F, Wen S, Jiang G, Xue Y, Dong L, Zhao L, Zhu R, Huang H**. **2021**. In vitro Antimicrobial Activity Comparison of Linezolid, Tedizolid, Sutezolid and Delpazolid Against Slowly Growing Mycobacteria Isolated in Beijing, China. *Infection and drug resistance* **14**:4689-4697 DOI 10.2147/IDR.S332835.

**Yum JH, Choi SH, Yong D, Chong Y, Im WB, Rhee DK, Lee K**. **2010**. Comparative in vitro activities of torezolid (DA-7157) against clinical isolates of aerobic and anaerobic bacteria in South Korea. *Antimicrobial agents and chemotherapy* **54(12)**:5381-5386 DOI 10.1128/AAC.00728-10.

**Zhang H, Hua W, Lin S, Zhang Y, Chen X, Wang S, Chen J, Zhang W**. **2022**. In vitro Susceptibility of Nontuberculous Mycobacteria to Tedizolid. *Infection and drug resistance* **15**:4845-4852 DOI 10.2147/IDR.S362583.

**Zhang H, Luo M, Zhang K, Yang X, Hu K, Fu Z, Zhang L, Wu P, Wan D, Han M, Wang X**. **2020**. Species identification and antimicrobial susceptibility testing of non-tuberculous mycobacteria isolated in Chongqing, Southwest China. *Epidemiology and infection* **149**:e7 DOI 10.1017/S0950268820003088.

**Zhang L, Pang Y, Yu X, Wang Y, Gao M, Huang H, Zhao Y**. **2014**. Linezolid in the treatment of extensively drug-resistant tuberculosis. *Infection* **42(4)**:705-711 DOI 10.1007/s15010-014-0632-2.

**Zhang Q, Wu S, Song P, Liu Y, Ding L, Shi Q, Zhu X, Zhang L, Zhang J, Wang Q, Xu J, Hu F**. **2023**. Antibiotic resistance and resistance mechanism of Corynebacterium kroppenstedtii isolated from patients with mastadenitis. *European journal of clinical microbiology & infectious diseases : official publication of the European Society of Clinical Microbiology* **42(4)**:525-528 DOI 10.1007/s10096-023-04558-0.

**Zhang Z, Lu J, Liu M, Wang Y, Zhao Y, Pang Y**. **2017**. In vitro activity of clarithromycin in combination with other antimicrobial agents against Mycobacterium abscessus and Mycobacterium massiliense. *International journal of antimicrobial agents* **49(3)**:383-386 DOI 10.1016/j.ijantimicag.2016.12.003.

**Zhang Z, Pang Y, Wang Y, Cohen C, Zhao Y, Liu C**. **2015**. Differences in risk factors and drug susceptibility between Mycobacterium avium and Mycobacterium intracellulare lung diseases in China. *International journal of antimicrobial agents* **45(5)**:491-495 DOI 10.1016/j.ijantimicag.2015.01.012.

**Zhang Z, Pang Y, Wang Y, Liu C, Zhao Y**. **2014**. Beijing genotype of Mycobacterium tuberculosis is significantly associated with linezolid resistance in multidrug-resistant and extensively drug-resistant tuberculosis in China. *International journal of antimicrobial agents* **43(3)**:231-235 DOI 10.1016/j.ijantimicag.2013.12.007.

**Zhao W, Jiang Y, Bao P, Li Y, Tang L, Zhou Y, Zhao Y**. **2015**. Evaluation of the Efficacy of Novel Oxazolidinone Analogues against Nontuberculous Mycobacteria In Vitro. *Japanese journal of infectious diseases* **68(6)**:520-522 DOI 10.7883/yoken.JJID.2014.498.

**Zheng H, He W, Jiao W, Xia H, Sun L, Wang S, Xiao J, Ou X, Zhao Y, Shen A**. **2021**. Molecular characterization of multidrug-resistant tuberculosis against levofloxacin, moxifloxacin, bedaquiline, linezolid, clofazimine, and delamanid in southwest of China. *BMC infectious diseases* **21(1)**:330 DOI 10.1186/s12879-021-06024-8.

**Zheng HW, Pang Y, He GX, Song YY, Zhao YL**. **2017**. Antimicrobial Susceptibility Testing and Molecular Characterization of Mycobacterium fortuitum Isolates in China. *Biomedical and environmental sciences : BES* **30(5)**:376-379 DOI 10.3967/bes2017.049.

**Zong Z, Jing W, Shi J, Wen S, Zhang T, Huo F, Shang Y, Liang Q, Huang H, Pang Y**. **2018**. Comparison of In Vitro Activity and MIC Distributions between the Novel Oxazolidinone Delpazolid and Linezolid against Multidrug-Resistant and Extensively Drug-Resistant Mycobacterium tuberculosis in China. *Antimicrobial agents and chemotherapy* **62(8)**:e00165-00118 DOI 10.1128/AAC.00165-18.
